# Cross-Disorder Analysis of De Novo Mutations in Neuropsychiatric Disorders

**DOI:** 10.1007/s10803-021-05031-7

**Published:** 2021-05-10

**Authors:** Kuokuo Li, Zhenghuan Fang, Guihu Zhao, Bin Li, Chao Chen, Lu Xia, Lin Wang, Tengfei Luo, Xiaomeng Wang, Zheng Wang, Yi Zhang, Yi Jiang, Qian Pan, Zhengmao Hu, Hui Guo, Beisha Tang, Chunyu Liu, Zhongsheng Sun, Kun Xia, Jinchen Li

**Affiliations:** 1 National Clinical Research Center for Geriatric Disorders, Department of Geriatrics, Xiangya Hospital, Central South University, Xiangya Road, Kaifu District, Changsha, 410013 Hunan China; 2 Center for Medical Genetics & Hunan Key Laboratory of Medical Genetics, School of Life Sciences, Central South University, Xiangya Road, Kaifu District, Changsha, 410013 Hunan China; 3 Department of Obstetrics and Gynecology, The First Affiliated Hospital of Anhui Medical University, No 218 Jixi Road, Hefei, 230022 Anhui China; 4 NHC Key Laboratory of Study on Abnormal Gametes and Reproductive Tract (Anhui Medical University), No 81 Meishan Road, Hefei, 230032 Anhui China; 5 Institute of Molecular Precision Medicine, Xiangya Hospital, Central South University, Xiangya Road, Kaifu District, Changsha, 410013 Hunan China; 6 Department of Psychiatry, SUNY Upstate Medical University, Syracuse, NY USA; 7 Beijing Institutes of Life Science, Chinese Academy of Sciences, Beijing, China; 8 Institute of Genomic Medicine, Wenzhou Medical University, Wenzhou, Zhejiang China; 9 School of Basic Medical Science, Central South University, Changsha, Hunan China; 10 CAS Center for Excellence in Brain Science and Intelligence Technology (CEBSIT), Shanghai, China

**Keywords:** Neuropsychiatric disorder, De novo mutation, Candidate gene, Expression pattern, Functional network

## Abstract

The clinical similarity among different neuropsychiatric disorders (NPDs) suggested a shared genetic basis. We catalogued 23,109 coding de novo mutations (DNMs) from 6511 patients with autism spectrum disorder (ASD), 4,293 undiagnosed developmental disorder (UDD), 933 epileptic encephalopathy (EE), 1022 intellectual disability (ID), 1094 schizophrenia (SCZ), and 3391 controls. We evaluated that putative functional DNMs contribute to 38.11%, 34.40%, 33.31%, 10.98% and 6.91% of patients with ID, EE, UDD, ASD and SCZ, respectively. Consistent with phenotype similarity and heterogeneity in different NPDs, they show different degree of genetic association. Cross-disorder analysis of DNMs prioritized 321 candidate genes (FDR < 0.05) and showed that genes shared in more disorders were more likely to exhibited specific expression pattern, functional pathway, genetic convergence, and genetic intolerance.

**Supplementary Information:**

The online version contains supplementary material available at 10.1007/s10803-021-05031-7.

## Introduction

Neuropsychiatric disorders (NPDs) are a group of disorders with brain dysfunction, leading to abnormal in cognition, behavior, mood and communication. The influence of NPDs to international public health is acknowledged, especially due to their high clinical complexity (Craddock & Owen, 2010; Willsey et al., 2018). The similarity and heterogeneity among different types of NPDs such as autism spectrum disorder (ASD), epileptic encephalopathy (EE), intellectual disability (ID), and schizophrenia (SCZ), undiagnosed developmental disorder (UDD) promote clinicians and researchers studying them. These disorders tend to strike before adolescence, in particular ASD, EE, ID, and UDD onset during the time of infant and child. (Forrest, Parnell, & Penzes, 2018). Their clinical phenotypes vary significantly among different patients from mild to severe impairment in many aspects of brain development and other organ system (Martin et al., 2018). NPDs are defined as distinct clinical classifications based on DSM-5 and ICD-11, whereas significant overlap of symptoms between different disorders (Moreno-De-Luca et al., 2013). For example, 20% of patients that meet the criteria of more than one disorder (Adam, 2013) and family history of one disorder increased the risk of another disorder (Sullivan et al., 2012). In addition, because of significant clinical similarity of ID and UDD, previous studies frequently combined individuals with these two disorders to explore genetic reasons (Coe et al., 2019; Satterstrom et al., 2020). Moreover, due to the dynamic nature of symptoms, patients often receive diagnoses of additional disorders, particular within the first year of the original diagnosis, and these pair comorbidities were bidirectional (Plana-Ripoll et al., 2019). All of these studies highlight the clinical similarity among NPDs, which implicated common etiological mechanisms.

Twins and adoption studies exhibited high heritability of NPDs, which provides the opportunity to understand their etiologies from genetic perspective (Polderman et al., 2015). The clinical similarity of NPDs might implicated by genetic. Recently, multiple studies employed whole-exome sequencing (WES) or whole-genome sequencing (WGS) to detect de novo mutations (DNMs) and successfully prioritized candidate genes with DNMs in ASD (An et al., 2018; Iossifov et al., 2014), UDD (Deciphering Developmental Disorders, 2017), EE (Epi et al., 2013), ID (Lelieveld et al., 2016), and SCZ (Fromer et al., 2014). Due to the strong functional effects of DNMs [16], some candidate genes have been established to be associated with specific clinical phenotypes, such as *CHD8* (Bernier et al., 2014) with ASD, ID, sleep problem, macrocephaly, and gastrointestinal symptoms, and *DYRK1A* (van Bon et al., 2016) with ID, microcephaly and febrile seizures infancy. Genotype–phenotype correlation analysis will strengthen the genetic evidence (Dong et al., 2014; Willsey et al., 2013); integration of DNMs that distributed in different publications was an effective method to increase sample size of patients carrying DNMs of specific disease gene (Li et al., 2016; Nguyen et al., 2017). In addition, consistent with SNP based genetic correlation, genes with DNMs also show of significant overlap between different classification of NPDs (Cross-Disorder Group of the Psychiatric Genomics, 2013; Schork et al., 2019). Moreover, genetic correlations among bipolar disorder, ASD and SCZ matched with clear similarity in transcriptomic features detected in the post-mortem brain (Gandal et al., 2018). These associations were replicated in our Chinese cohorts (Guo et al., 2018, 2019; Wang et al., 2016). Therefore, integrating data from multiple disorders with phenotypic similarity increased the statistical power of candidate gene discovery (Coe et al., 2019; Gonzalez-Mantilla et al., 2016; Li et al., 2016).

The functional analysis of candidate genes from both expression and functional networks provide clues to elucidate the molecular pathway related to these disorders, such as the brain-size-related genes (Li et al., 2017a), Vitamin D-related genes (Li et al., 2017b) and recessive genes (Wang et al., 2020) in ASD, as well as the genetic components related to three ASD subcategories (Li, Hu, et al., 2018), we previously reported. In addition, functional genomics of NPDs will reveal the characteristics of pleiotropic genes from disorder-specific genes, which may advance the diagnostic classification and treatment (Cross-Disorder Group of the Psychiatric Genomics Consortium. Electronic address and Cross-Disorder Group of the Psychiatric Genomics, 2019). By searching the scientific literature, we collected DNMs from 13,853 NPD cases and 3391 controls to perform a cross-disorder analysis of five types of NPDs: ASD, UDD, EE, ID and SCZ. We want to decipher perspectives of NPDs as follow: (1) the burden and contribution of DNMs in NPDs; (2) the prioritization of candidate genes; (3) the expression patterns and functional pathways of candidate genes.

## Materials and Methods

### Data Collection and Annotation

We collected DNMs detected by WGS or WES from 37 published studies and performed cross-disorder analysis (Table S1). We searched original articles in PubMed from 2010 to 2019 based on the terms of “*de novo* mutation”, “whole-exome sequencing” and “whole-genome sequencing”. The DNMs in ASD, UDD, EE, ID and SCZ were used for further analysis, because only these five NPDs have enough DNMs. Additionally, DNMs detected in unaffected individuals were also collected as negative control. ANNOVAR (Wang, Li, & Hakonarson, 2010) and our previously reported VarCards (Li, Shi, et al., 2018) were utilized to annotate DNMs based on a human reference genome (hg19). Based on the functional effects of variants, we classified DNMs into two classes as follow: (1) Coding region variant, including loss-of-function variant (LoF, including splicing (≤ 2 bp), stopgain, and stoploss SNVs, and frameshift indels), deleterious missense variant (Dmis), tolerant missense variant (Tmis), synonymous variant, (2) Noncoding region variant. The pathogenesis of missense variant was predicted by ReVe, recently developed by our group (Li, Zhao, et al., 2018). LoF and Dmis variant were combined as putative functional (Pfun) variants. We only focused on coding region variant in further analysis. All variants were available in our currently developed Gene4Denove database (Zhao et al., 2019).

### Burdens and Contributions Analysis of Different Types of DNMs

To test which classes of DNMs contribute to each disorder, two-tailed Fisher’s exact test was used to compare the burden of DNMs of each NPD with unaffected control. As DNM data was collected from different publications, we normalized DNMs by the number of de novo synonymous variants based on the hypothesis that synonymous variants would be unrelated to phenotype and could potentially remove batch effects of DNM detection rate. To perform burden analysis, we constructed four number as follow: the count of specific class of DNMs (Dmis, LoF, Pfun) in case and control, and the count of de novo synonymous in case and control. Additionally, we used “ascertainment differentials” as described in previous work (Iossifov et al., 2014) to estimate the contribution of each class of DNMs to different NPDs and synonymous variants also were used to remove bias between different studies.

### Gene Set Overlap Across Five Disorders Based on De Novo Mutations

We also used DNENRICH (Fromer et al., 2014; Shohat, Ben-David, & Shifman, 2017) to test whether genes that were detected to carry specific classes of DNMs (i.e., LoF, Dmis, and Pfun) in any two NPDs show of significant overlap. DNENRICH was a software that perform gene set enrichment based on gene size, structure, and local trinucleotide mutation rate. We performed 100,000 permutations which weight mutation number of each gene and compared observed and expected overlap of two gene set. The input of DNENRICH includes (1) gene carrying specific class of DNMs in one disorder, (2) genes carrying consistent class of DNMs in another disorder. The Benjamini and Hochberg false discovery rate procedure was used to adjust for multiple testing.

### Candidate Genes Prioritization Based on TADA

The transmitted and de novo association (TADA) tool was used to perform statistical analysis based on recurrent of variants in same gene (He et al., 2013). Here, we used TADA-Denovo which only take DNMs into consideration. To prioritized candidate genes, we used two strategies in our analysis. Firstly, we performed TADA analysis of DNMs in each disorder. Secondly, we performed TADA analysis of the DNMs in five NPDs based on the hypothesis that NPDs shared genetically components. Genes with FDR (q-value) < 0.05 in either of above two strategies were prioritized as candidate genes. Genes with Pfun DNMs in at least two disorders were classified as shared genes, and genes with DNMs in only one disorder were classified as unique genes.

### Expression Patterns and Functional Networks Analysis

As in our previous study (Li et al., 2017a), we performed weighted gene coexpression network package (WGCNA; Langfelder & Horvath, 2008) to RNA-seq data of 524 brain samples from the BrainSpan database with the power of six to depict the spatiotemporal expression patterns of candidate gene. In addition, we sourced transcriptome data of 526 prenatal neocortical samples from the BrainSpan database with the power of three to characterize neocortical expression profiles of candidate gene. The other consensus parameters of WGCNA for spatiotemporal expression pattern and prenatal neocortical samples are as follow: minModuleSize = 20, mergeCutHeight = 0.25, corType = “pearson”. In addition, to construct functional networks of candidate genes, we calculated the Pearson correlation coefficients between any two genes based on the RNA-seq data of 524 brain samples from the BrainSpan database mentioned above, and gene pairs with |R|> 0.8 were regarded as expressed. Protein–protein interaction (PPI) data with a combined score higher than 400 according to the version 10.5 of STRING (Szklarczyk et al., 2019) database were also incorporated to construct a functional network. Cytoscape v3.6.1 (https://cytoscape.org/) was used to visualize the functional network.

### Gene Functional Enrichment

To investigate the function of candidate gene, we used Metascape tool (http://metascape.org/) with default parameters to perform functional enrichment analysis in three gene ontology (GO) including molecular function, cellular component and biological process for all candidate genes (Zhou et al., 2019). In addition, we performed functional enrichment of candidate genes in each independent coexpression module identified by WGCNA to identify specific function.

## Results

### Pfun DNMs Involved in the Five NPDs with Burden and Contribution

In total, we collected 23,110 DNMs in coding regions from 17,244 trio-based WES/WGS studies, including 8175 DNMs from 6511 patients with ASD, 7696 DNMs from 4293 patients with UDD, 1165 DNMs from 933 patients with EE, 1393 DNMs from 1022 patients with ID, 1052 DNMs from 1094 patients with SCZ, and 3629 DNMs from 3391 unaffected controls (Table S1). The DNMs were available in Gene4Denove database, recently developed by our group (Zhao et al., 2019). After controlling for batch effects with synonymous variants, we found that all the five NPDs carried significantly more LoF and Dmis DNMs, as well as the combination of these two classes of DNMs (i.e., Pfun) but not Tmis DNMs, suggesting that Pfun DNMs are involved in all the five disorders (Table 1; Fig. S1). However, different degree of mutation burden for the five NPDs. First, we found that Pfun DNMs exhibited a high degree of mutation burden in patients with EE (odds ratio, OR = 2.45), ID (OR = 2.36), and UDD (OR = 1.77). Second, Pfun DNMs in ASD (OR = 1.33) were moderately higher than those in the control. Thirdly, SCZ (OR = 1.28) presented with the lowest difference of Pfun compared to the control.

**Table 1:** Burdens and contributions of different classes of DNMs in five disorders

Disorders (N)	Category	LoF	Dmis	Pfun	Tmis	Synonymous
ASD (6511)	DNMs	1228	1633	2861	3406	1864
	*p*	6.80E−09	6.00E−04	9.81E−08	0.20	
	*p*_adj_	**2.72E−08**	**8.00E−04**	**1.96E−07**	0.20	
	OR	1.49	1.23	1.33	1.07	
	95% Cl	1.30–1.72	1.09–1.39	1.20–1.48	0.97–1.18	
	Clinical implicated DNMs (%)	33.06	18.92	24.99		
	Contribute to patients (%)	6.24	4.75	10.98		
UDD (4293)	DNMs	1,382	1,898	3,280	2676	1607
	*p*	2.11E−22	6.66E−17	6.15E−26	0.60	
	*p*_adj_	**4.22E−22**	**8.88E−17**	**2.46E−25**	0.60	
	OR	1.95	1.66	1.77	0.97	
	95% Cl	1.70–2.24	1.47–1.88	1.59–1.97	0.88–1.08	
	Clinical implicated DNMs (%)	48.72	39.86	43.59		
	Contribute to patients (%)	15.68	17.62	33.31		
EE (933)	DNMs	192	350	542	414	192
	*p*	5.03E−12	1.66E−20	1.90E−22	0.018	
	*p*_adj_	**6.71E−12**	**3.32E−20**	**7.60E−22**	**0.018**	
	OR	2.27	2.57	2.45	1.26	
	95% Cl	1.79–2.88	2.09–3.16	2.03–2.97	1.04–1.53	
	Clinical implicated DNMs (%)	55.90	61.03	59.22		
	Contribute to patients (%)	11.50	22.90	34.40		
ID (1022)	DNMs	309	366	675	447	248
	*p*	7.94E−24	2.91E−14	2.23E−24	0.59	
	*p*_adj_	**1.59E−23**	**3.88E−14**	**8.92E−24**	0.59	
	OR	2.82	2.08	2.36	1.05	
	95% Cl	2.2–3.48	1.71–2.52	1.99–2.81	0.88–1.26	
	Clinical implicated DNMs (%)	64.61	51.87	57.70		
	Contribute to patients (%)	19.53	18.58	38.11		
SCZ (1094)	DNMs	136	217	353	450	241
	*p*	0.045	0.028	0.011	0.35	
	*p*_adj_	0.060	0.056	**0.044**	0.35	
	OR	1.28	1.27	1.27	1.09	
	95% Cl	1.00–1.64	1.02–1.57	1.05–1.54	0.91–1.31	
	Clinical implicated DNMs (%)	21.85	21.11	22.40		
	Contribute to patients (%)	2.72	4.19	6.91		
NPDs (13,853)	DNMs	3247	4464	7711	7393	4152
	*p*	3.53E−20	4.68E−14	1.85E−22	0.39	
	*p*_adj_	**7.06E−20**	**6.24E−14**	**7.40E−22**	0.39	
	OR	1.77	1.51	1.61	1.04	
	95% Cl	1.56–2.01	1.36–1.69	1.46–1.78	0.95–1.14	
	Clinical implicated DNMs (%)	43.61	33.93	38.01		
	Contribute to patients (%)	10.22	10.94	21.16		
Control (3391)	DNMs	411	662	1073	1595	932

“Clinical implicated DNMs” means the estimated proportion of DNMs in each disorder involved in the etiology of disorders. “Contribute to patients” means the proportion of patients can be interpreted by DNMs based on the clinical implicated DNMs. We performed a Fisher exact test, which normalizes by the number of de novo synonymous mutations in each condition to adjust the batch effects in different studies. The Benjamini and Hochberg false discovery rate (FDR) procedure was used to adjust for multiple testing. *p*_adj_ below 0.05 were highlighted in bold

*ASD* autism spectrum disorder, *UDD* undiagnosed developmental disorder, *EE* epileptic encephalopathy, *ID* intellectual disability, *SCZ* schizophrenia, *NPDs* neuropsychiatric disorders, integration of these five disorders, *DNMs* de novo mutations, *OR* odds ratio, *CI* confidence interval, *Dmis* deleterious missense variants as predicted by ReVe, *Tmis* tolerant missense variants, *LoF* loss-of-function variants including frameshift, stoploss and stopgain, splicing variants, *Pfun* putative functional variant including Dmis and LoF variants

We then evaluated the contribution of Pfun DNMs to NPDs based on “ascertainment differentials” of DNMs, a method based on the bias of DNMs events per child between patients and controls, as previous study (Iossifov et al., 2014). As a result, we found that 43.61% LoF and 33.93% Dmis DNMs contribute to 10.22% and 10.94% of overall NPD patients, respectively (Table 1). Specifically, 64.61%, 55.90%, 48.72%, 33.06% and 21.85% of LoF DNMs contribute in 19.53% of ID, 11.50% of EE, 15.68% of UDD, 6.24% of ASD, and 2.72% of SCZ patients, respectively. In addition, we noted that 61.03%, 51.87%, 39.86%, 18.92%, 21.11% of Dmis DNMs contribute in 22.90%, 18.58%, 17.62%, 4.75%, and 4.19% of EE, ID, UDD, ASD and SCZ patients, respectively. Together, Pfun DNMs presented a gradient of effect sizes and contributed in 38.11%, 34.40%, 33.31%, 10.98% and 6.91% of patients with ID, EE, UDD, ASD and SCZ, respectively. These results suggested that DNMs play strong roles in the etiology of ID, EE, and UDD, and moderate roles in ASD, compared to general roles in SCZ.

### Genetic Similarity Among the Five Disorders

**Fig. 1:**
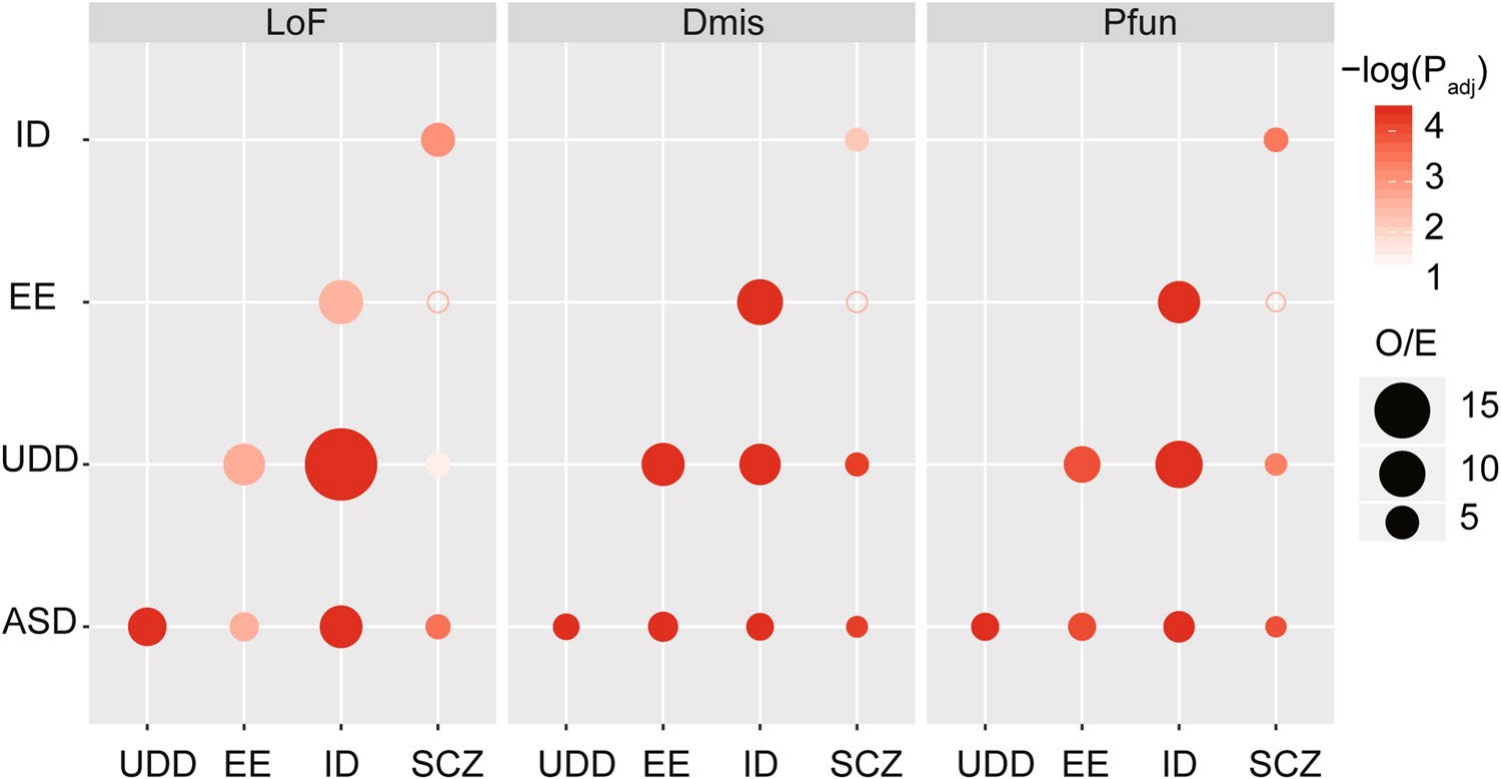
Overlap of genes across five NPDs based on de novo mutations. Overlap of genes among disorders were performed based on three classes of variants include LoF, Dmis and Pfun. *O*/*E* ratio of observed to expected numbers of shared genes, *Dmis* Deleterious missense variants, *Tmis* Tolerant missense variants, *LoF* loss of function. LoF include frameshift, stoploss and stopgain, splicing variants, *Pfun* Putative functional variants, including Dmis and LoF variants. The Benjamini and Hochberg false discovery rate (FDR) procedure was used to adjust for multiple testing

Due to the clinical similarity between NPDs, we explored genes overlap among the five disorders based on DNMs with different functional effects. We found that genes with LoF, Dmis, as well as Pfun DNMs were significantly shared among the five disorders. However, a compellingly divergent degree of genes overlap was also observed (Fig. 1; Table S2). Specifically, we found a high degree of overlapping Pfun genes between UDD and ID [p_adj_ = 3.85E−5, with an observed-to-expected ratio (O/E) = 10.51], EE and ID [p_adj_ = 3.85E−5, (O/E) = 8.21], and UDD and EE [p_adj_ = 1.11E−4, (O/E) = 6.03]. We also observed that ASD showed a secondary (lesser) degree of genetic overlap with ID [p_adj_ = 3.85E−5, (O/E) = 4.33], EE [p_adj_ = 9.73E−5, (O/E) = 3.36] and UDD [p_adj_ = 3.85E−5, (O/E) = 3.35]. Although SCZ and EE showed significant overlap with ASD, ID and UDD, but SCZ and EE [(O/E) = 1.59, p_adj_ = 0.097] were only very slightly genetically overlap with each other.

### Cross Disorder Analysis Prioritize Novel Candidate Gene

Based on the TADA model for Pfun DNMs in five disorder, we prioritized 279 candidate genes (FDR < 0.05), containing 59, 202, 43, and 66 genes in ASD, UDD, EE, and ID, respectively (Table S3). Due to the smaller number of sample size and less contribution of DNMs, we did not prioritize any candidate genes in SCZ by single-disorder analysis. Since these five disorders presented significant genetic similarity, we also integrated Pfun DNMs in these five disorders. As a result, we prioritized 238 candidate genes with FDR < 0.05, including 42 novel candidate genes that were not included in the above 279 candidate genes (Table S3). In addition, we also noted that 43 of the 238 candidate genes showed stronger statistical evidence by integrated analysis. After removing redundancy between the above two strategies, 321 candidate genes were finally prioritized (Table 2). We found that Pfun DNMs of these candidate genes account for 27.37%, 26.81%, 21.54%, 7.14%, and 3.20% patients with UDD, ID, EE, ASD, and SCZ, respectively (Table S4). For example, *SCN2A* carrying the largest number of Pfun (*n* = 48), accounts for 0.40% (*n* = 17), 0.88% (*n* = 9), 0.94% (*n* = 9), 0.18% (*n* = 12), 0.091% (*n* = 1) of patients with UDD, ID, EE, ASD and SCZ, respectively.

**Table 2:** Prioritized candidate genes with FDR < 0.05 in this study

Rank	Unique genes (26.48%, *n* = 85)	Shared genes in two disorders (32.71%, *n* = 105)	Shared genes in three disorders (27.10%, *n* = 87)	Shared genes in at least four disorders (13.71%, *n* = 44)
FDR ≤ 0.0001 (39.25%, *n* = 126)	HDAC8^u^, KANSL1^u^, ZBTB18^u^, CNKSR2^u^, BTF3^u^, MSL3^u^, PDHA1^u^	SATB2, GATAD2B, KAT6B, MEF2C, SMC1A, CDKL5, SMARCA2, KDM5B, NSD1, EHMT1, HNRNPU, PTPN11, CTCF, CNOT3, TBR1, NFIX, PPP1CB, KIF1A, GNAI1, CHAMP1, KCNH1, NAA15*, UPF3B, PIK3CA**, MAP4K4, UNC80*, KCNT1, KDM6A, ZC4H2, SMAD4, WDR26**, SOX11	SLC6A1, FOXP1, CTNNB1, TCF4, SETD5, PPP2R5D, ASXL3, MED13L, SCN1A, DYRK1A, ADNP, EP300, PURA, WDR45, CDK13, TBL1XR1, IRF2BPL, PTEN, KAT6A, DNM1, PACS1, SHANK3, TRIP12*, PPM1D, NAA10, TCF20, CLTC, SET*, BRAF, CACNA1A, BCL11A, CHD3, EFTUD2, SMARCA4*, SOX5, HECW2, KMT5B**, FBXO11**, USP9X, DLG4, PBX1***, MYT1L**, TCF7L2**, NR2F1**, ATP1A3***, SLC35A2**, NALCN, NSD2**, ANK2	CHD8, CHD2, POGZ, STXBP1, KCNQ2, KMT2A, ANKRD11, DDX3X, ARID1B, SCN8A, GRIN2B, CSNK2A1, WAC, FOXG1, CASK, IQSEC2, AHDC1, EEF1A2, TLK2*, DNMT3A*, GABRB2, CREBBP, COL4A3BP, PUF60, CACNA1E*, GABRB3**, KIAA2022***, CUL3**, SMC3*, PHIP, CHD4, ITPR1, KCNQ3*, AGO1***, MECP2, SYNGAP1, SCN2A, GNAO1
0.0001 < FDR ≤ 0.001 (10.59%, *n* = 34)	GFOD2^u^, MYO1E^a^, TAOK1^u^, PRKAR1A^u^, AKT3^u^, SLC12A2^u^, HNRNPK^u^, AGO2^i^	QRICH1*, EBF3, PPP2R1A, CAMK2A*, RAB11A, RAC1, SNAP25*, USP7, TNPO2, SYT1, SIN3A	FOXP2*, HIST1H1E*, GABBR2, MBD5**, SETBP1, ZMYND11*, SLC22A23*, KCNB1, CSNK1E*, SYNCRIP*, RFX3**, LOC400927-CSNK1E*, DHDDS*	GRIN2A, NACC1
0.001 < FDR ≤ 0.01 (16.82%, *n* = 54)	TAB2^u^, MORC2^u^, WDFY3^a^, TCF12^u^, PRKG1^u^, SIX3^u^, BRD7^a^, SOX4^u^, ASB14^a^, KCNA2^e^, C9orf142^u^, PHF5A^u^, C1orf123^u^, LMO2^u^, VAMP2^u^, PLAC8L1^u^, HIST1H4C^u^, SNX11^u^	CYP27C1, ARHGEF9, HK1, POU3F3, FGF12, CBL, PPP2CA, CSNK2B, SF1, TNPO3, ASXL1, PRKD1*, ERI1, HIST1H4E, CAMK2B, AUTS2, PHF7, NTRK2, FANCE, RNF146, LAMB1*, FAM200A, SSBP3, PRPF8, FIGN,	YWHAG*, GABRA1, SRCAP*, TUBA1A, BRPF1, GABRG2, CPSF7, SPAST, NRXN1*, DSCAM	DYNC1H1
0.01 < FDR ≤ 0.05 (33.33%, *n* = 107)	EBF2^e^, PDX1^u^, PLEKHB2^u^, HIST1H2AC^u^, TAF13^s^, FAM104A^u^, SMPD4^e^, MPPED2^u^, GOLPH3^u^, RAD51^u^, FOSL2^u^, GALNT18^a^, PI4K2B^u^, PRSS48^u^, PRKAR1B^a^, SIAH1^u^, LARP7^u^, EIF4A2^u^, COL23A1^u^, ATP8A1^i^, RAB11B^u^, GIGYF1^a^, MSI1^u^, SMPD2^u^, PISD^u^, ZBTB10^u^, DCAF7^u^, NONO^u^, RPUSD1^u^, GRIA2^a^, TMEM26^u^, FXYD5^a^, IL1RAPL2^u^, TGFB2^u^, NFE2L2^u^, ACHE^a^, FAM84A^u^, PAPOLG^a^, PNKD^u^, ERBB4^u^, CLDN5^u^, ATP1B1^a^, SGCE^u^, NUDT17^a^, SNRPB2^i^, ACTC1^u^, GABRP^u^, GNB1^u^, ILF2^a^, LRRC3C^i^, NR6A1^u^, STK33^a^	ANO3, G3BP1, CLCN4, DCX, MAP2K1, VEZF1, ASH1L, SMARCD1, KCNQ5, ENO1, STXBP3, LZTR1, AGO3, RPL26, GRIK1, CELF2, EYA1, PDK2, TFAP2C, COL4A1, LMTK3, PRR14L, TCTE3, NUDT4, GLRA2, ARIH1, GNAS, MEIS2, BIRC5, DOCK1, TIFA, DPF2, H2AFV, ABI2, MECOM, CFAP45, TFAP4	ARHGAP15, SMAD6, DEAF1, NR4A2, TANC2, GNB2, SMARCC2, TAF1, KIF5C, RPL4, PSD3, MARK2, PHF21A, UGT1A3, SPRED2	SON, GRIN1, RBM12

Candidate genes are split into four parts based on the number of disorders with putative functional DNMs in specific gene. Unique genes with superscripts letters including a, u, e, i, s represents genes only carry putative functional DNMs in ASD, UDD, EE, ID, SCZ respectively. We ranked all candidate genes into four tiers based on the strength of FDR. The number of asterisks on the genes indicate the increased ranks of candidate genes by cross disorders analysis compared to individual disorder analysis

Based on the strength of the statistical evidence (FDR), we ranked candidate genes into four tiers, namely: tier 1 (FDR ≤ 0.0001, *n* = 126), tier 2 (0.0001 < FDR ≤ 0.001, *n* = 34), tier 3 (0.001 < FDR ≤ 0.01, *n* = 54), and tier 4 (0.01 < FDR < 0.05, *n* = 107). In addition, 26.48% (85/321) of candidate genes carrying Pfun DNMs in only one disorder and 32.71% (105/321), 27.10% (87/321), 13.71% (44/321) of candidate genes carrying Pfun DNMs in two, three, and at least four disorders, respectively (Table 2). For example, *ATP1A3* (FDR = 2.61E−5), *PBX1* (FDR = 3.95E−6) carried Pfun DNMs in three disorders and *KIAA2022* (FDR = 6.34E−7), *AGO1* (FDR = 3.31E−6), *NACC1* (FDR = 4.38E−4) in four disorders, and increase significant genetic evidence from combined analysis. To test the disorder bias (one disorder harbor significant enrichment of DNMs in specific gene than other disorders) of specific candidate gene, we performed Poisson rate test as our previous studies (Li et al., 2016). For the 236 candidate genes carrying Pfun DNMs in at least two disorders, we found that 51 genes also presented disorder bias, including 33 genes in UDD, 11 genes in EE, seven genes in ID (Table S5).

In three recent studies(Coe et al., 2019; Gonzalez-Mantilla et al., 2016; Nguyen et al., 2017) which integrated rare variants and/or DNMs in multiple NPDs to identify risk genes, 64.49% (207/321) of candidate genes were identified, compared to 35.51% (114/321) genes being identified here (Fig. S2). Among these 114 novel candidate genes, 5, 9, 32, 68 genes were classified into tier 1 (*KIAA2022*, *KCNT1*, *ATP1A3*, *NALCN*, *SOX11*), tier 2 (*GFOD2*, *SLC22A23*, *SNAP25*, *NACC1*, *RFX3*, *AKT3*, *LOC400927-CSNK1E*, *DHDDS*), tier 3, and tier 4, respectively. We performed literature searches to find genetic or functional evidence of these novel candidate genes, and found 54.39% (62/114) of which were reported associated with human brain disorders. Moreover, 85.71% (12/14) of novel candidate genes in tier 1 and tier 2 associated with neurodevelopmental disorders (Table S6).

### Expression Patterns and Characteristics of Candidate Gene

**Fig. 2:**
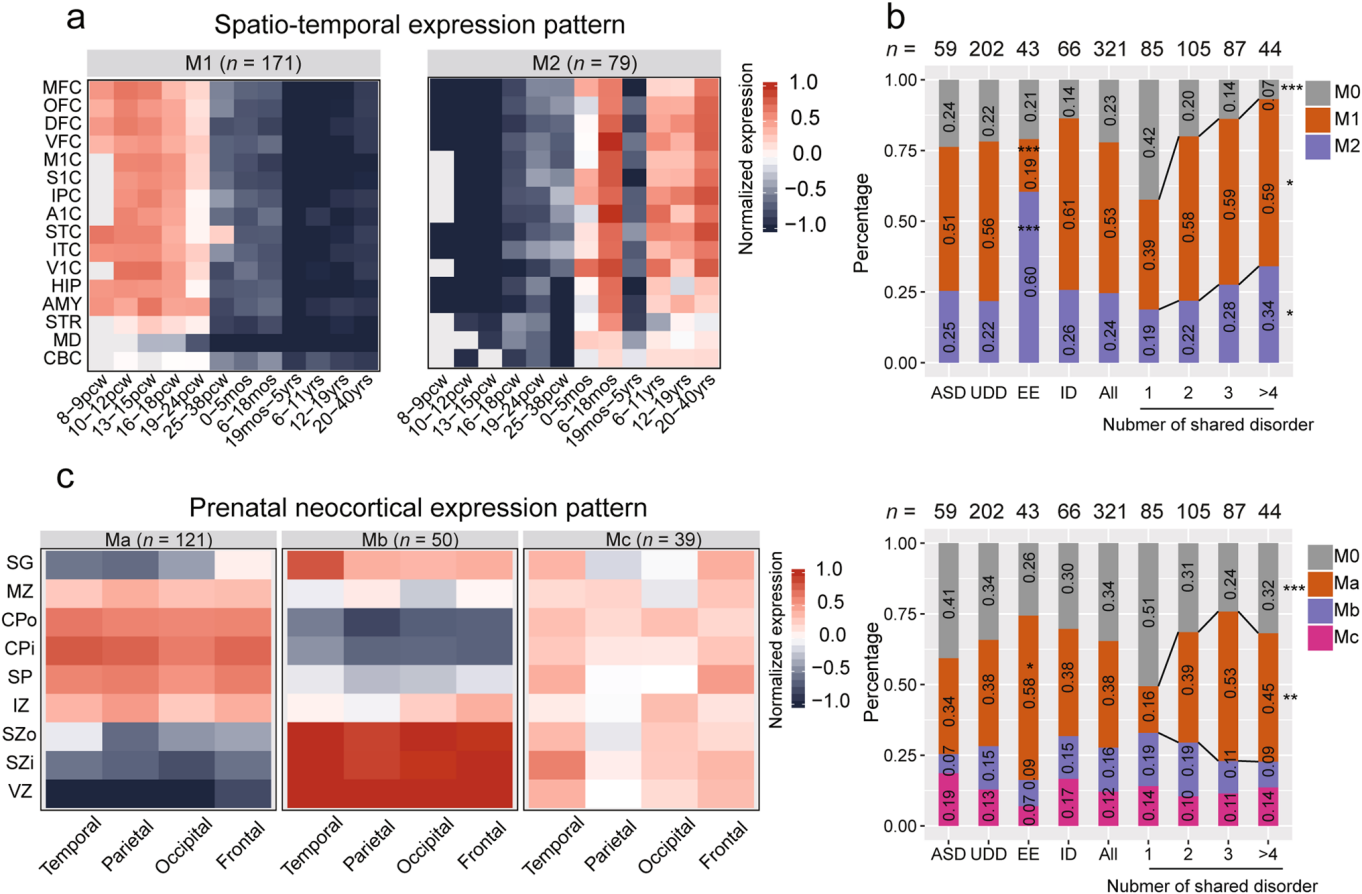
Expression characteristics of candidate genes in the human brain. **a** Spatiotemporal expression pattern of candidate genes based on RNA-seq data from BrainSpan. 250 of 321 Candidate genes could be classified into two co-expression modules: M1 (n = 171) and M2 (n = 79). *MFC* medial prefrontal cortex, *OFC* orbital frontal cortex, *DFC* dorsolateral prefrontal cortex, *VFC* ventrolateral prefrontal cortex, *M1C* primary motor cortex, *S1C* primary somatosensory cortex, *IPC* inferior parietal cortex, *A1C* primary auditory cortex, *STC* superior temporal cortex, *ITC* inferior temporal cortex, *V1C* primary visual cortex, *HIP* hippocampus, *AMY* amygdala, *STR* striatum, *MD* mediodorsal nucleus of thalamus, *CBC* cerebellar cortex. **b** Distribution of candidate genes at different conditions in the spatiotemporal coexpression modules. Candidate genes per disorder refers to genes with FDR < 0.05 based on putative functional DNMs in each individual disorder (ASD, UDD, EE, ID). Number of shared disorders refers to candidate genes in modules carrying Pfun in one, two, three or more than four disorders (1, 2, 3, > 4). We used the number of all candidate genes with expression in RNA-seq data of BrainSpan to set the background (All). **c** Neocortical expression pattern of candidate genes based on microarray data from micro-dissected human prenatal neocortex. A total of 210 candidate genes could be classified into three co-expression modules: Ma (n = 121), Mb (n = 50) and Mc (n = 39). *SG* subpial granular zone, *MZ* marginal zone, *CPo* outer cortical plate, *CPi* inner cortical plate, *SP* subplate zone, *IZ* intermediate zone, *SZo* outer subventricular zone, *SZi* inner subventricular zone, *VZ* ventricular zone. **d** Distribution of candidate genes in different conditions in the modules. Candidate genes per disorder refers to genes with FDR < 0.05 based on putative functional DNMs in individual disorders (ASD, UDD, EE, ID). Number of shared disorders refers to candidate genes carry Pfun in one, two, three or more than four disorders (1, 2, 3, > 4). We used the number of all candidate genes with expression in microarray data to set background levels (All). *M0* candidate genes not be included into coexpression modules. *p < 0.05; **p < 0.01; ***p < 0.001

The spatiotemporal expression patterns of candidate genes represent a quantitative phenotype that provides an in-depth view of the molecular pathways disrupted in NPDs. Based on transcriptomic data of brain samples from the BrainSpan database, we found that 250 of 321 genes could be classified into two independent modules (M1–M2) with highly similar expression patterns during development (Fig. 2a; Table S7). Genes in M1 (68.4%, 171/250) exhibited high expression in embryonic and early-to-middle fetal periods [8–24 post-conceptual weeks (pcw)] and then gradually decreased. In contrast, candidate genes in M2 (31.6%, 79/250) showed low expression during prenatal periods. We then tested whether candidate genes with FDR < 0.05 in each disorder were significantly enriched in specific modules compared to all candidate genes in the coexpression modules and found that 26 of 43 (60.47%) EE genes belonged to M2 (Fisher’s exact test, p = 7.07E−6, OR = 4.66, 95% CI 2.30–7.67) and only 8 of 43 (18.60%) EE genes in M1 (Fisher’s exact test, p = 1.55E−5, OR = 4.97, 95% CI 2.18–12.79) (Fig. 2b). In addition, genes with Pfun DNMs existing in several disorders were more likely to belong to coexpression modules (Ordered logistic regression, p = 2.07E−7) including chromatin related M1 (Ordered logistic regression, p = 1.08E−2) and synapse related M2 (Ordered logistic regression, p = 3.81E−2), suggesting the profound effect of chromatin and synapse on the etiology of multiple disorders.

Since the expression levels of candidate genes changed dramatically in the human brain during the fetal period (described above), we characterized the profiles of candidate genes based on prenatal neocortical samples from the BrainSpan database. Here, we identified three modules (Ma–Mc) involved in 210 of 321 candidate genes with distinct laminar neocortical expression patterns (Fig. 2c; Table S7). Genes in Ma (57.62%, 121/210) were high expressed in the middle to upper layers. However, genes in Mb (23.81%, 50/210) presented a relatively reversed expression pattern compared to Ma. Genes in Mc (18.57%, 39/210) showed a relatively stable expression level in different layers. Similar to spatio-temporal expression patterns described above, convergent and divergent features were observed in different disorders (Fig. 2d). Specifically, 58.14% (25/43) of EE genes belonged to Ma (Fisher’s exact test, p = 0.013, OR = 2.29, 95% CI 1.15–4.66). In addition, genes carrying Pfun DNMs in a higher number of disorders were more likely to be enriched coexpression modules (Ordered logistic regression, p = 1.60E−3), particularly in Ma (Ordered logistic regression, p = 6.59E−6), along with a concurrent enrichment in synaptic related function.

### Functional Network and Characteristics of Candidate Gene

**Fig. 3:**
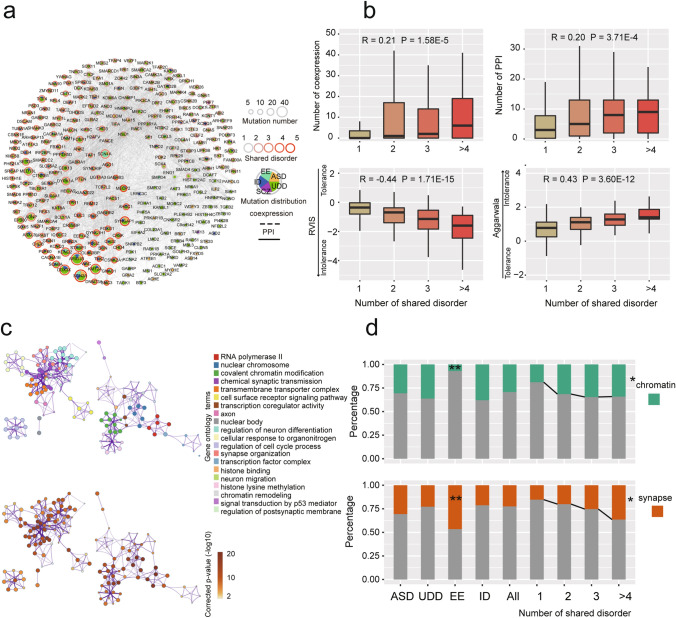
Functional network of candidate genes. **a** A network representation to show connectivity between candidate genes based on co-expression and protein–protein interactions (PPI). Dotted lines and full lines between nodes represent co-expression and PPI, respectively. The node size and color of the node boundary represent the number of putative functional variants and shared disorders for specific genes. Colors within nodes indicate the distribution of putative functional variant in each disorder. **b** Box plots for the relationship between the number of candidate genes connected with others, and the gene intolerance score, and the number of shared disorders. Top, co-expression and protein–protein interactions; bottom, residual variation intolerance score (RVIS) and substitution intolerance scores from Aggarwala et al., Nature Genetics 2016. **c** Top 20 clusters of functional enrichment for candidate gene (gene ontology terms with a similarity > 0.3 were merged into one cluster). **d** Distribution of genes in different conditions in the clusters related to their chromatin and synaptic function. Candidate genes per disorder refers to genes with FDR < 0.05 based on putative functional DNMs for individual disorders (ASD, UDD, EE, ID). Number of shared disorders refers to all candidate genes carrying Pfun in one, three or more than four disorders (1, 2, 3, > 4). We used the number of all candidate genes to set the background (All). *p < 0.05; **p < 0.01

We next constructed a network which contained 84.11% (270/321) of the candidate genes that were co-expressed at mRNA level and/or displayed interactions at the protein level (Fig. 3a). We noted that 208 of 270 genes (77.04%) in the functional network were shared genes, compared to 28 of 51 genes (54.90%) that were not included in the network (Fisher’s exact test p = 7.18E−3, OR = 2.51, 95% CI 1.26–4.94). In addition, genes in the network carrying Pfun DNMs in a higher number of disorders showed more interconnectedness with others based on both coexpression data (Spearman’s rank correlation = 0.21, p = 1.58E−5, Fig. 3b) and protein–protein interaction data (Spearman’s rank correlation = 0.20, p = 3.71E−4, Fig. 3b) suggesting that shared genes were more likely to be hub gene. Moreover, we found that genes shared by more disorders were more likely to be intolerant to mutation based on two different methods, residual variation intolerance score (RVIS; Petrovski et al., 2013) (Spearman’s rank correlation =  − 0.44, p = 1.71E−15, Fig. 3b) and Aggarwala constraint metrics (Aggarwala & Voight, 2016) (Spearman’s rank correlation = 0.43, p = 3.60E−12, Fig. 3b). These results suggested that genetic variants of shared genes may presented higher penetrance by regulating more genes.

Subsequently, we performed functional annotation and identified several gene ontology (GO) terms involved in NPDs based on the 321 candidate genes, including chromatin, chromosome, synapse and neuron related function (Fig. 3c). We found that EE genes were more likely to be involved in synapse relation function (20/43, Fisher’s exact test, p = 1.28E−3, OR = 3.08, 95% CI 1.48–6.37), but not chromatin (3/43, Fisher’s exact test, p = 1.43E−3, OR = 5.50, 95% CI 1.69–28.47) (Fig. 3d). In addition, candidate genes with Pfun DNMs in multiple disorders were more likely to have enriched chromatin function (Ordered logistic regression, p = 0.023) as well as synaptic function (Ordered logistic regression, p = 0.014), suggesting essential roles for these two pathways in the common etiology in NPDs.

## Discussion

The high heritability of NPDs provides an opportunity to understand their etiologies from a genetic perspective. DNMs with strong functional effects have been widely demonstrated to significantly contribute to the etiology of NPDs. Therefore, we try to integrate DNMs data from five NPDs with phenotypic similarity to identify novel candidate gene and decipher the association among NPDs from new perspectives.

Our data showed that Pfun DNMs rather than Tmis and synonymous DNMs play broad roles in the five NPDs, along with compellingly different degrees of DNM burden and contribution to phenotypes. First, ID, EE and UDD presented a high degree of DNMs burden and we estimated that Pfun DNMs contributed to these NPDs in more than 30% of patients. Our results strengthen previous studies which showed that DNMs result in a diagnostic yield of 42%, 40% and 26.9% for UDD (Deciphering Developmental Disorders, 2017), ID (Gilissen et al., 2014), and EE (Hamdan et al., 2017), respectively. Second, ASD presented a moderate degree of DNMs burden, and Pfun DNMs contributing in about 10% of patients with ASD. Other genetic variants might be involved in the etiology of ASD, such as de novo promoter variants (An et al., 2018) and common variants (Grove et al., 2019). Additionally, patients with ASD also present phenotype of developmental delay and ID (Iossifov et al., 2014; Martin et al., 2018). Currently study found that patients carrying DNMs in ASD-NDD genes (gene carrying more DNMs in other neurodevelopmental disorder than ASD) exhibited later walk and lower IQ than patients carrying DNMs in ASD-predominant genes (gene carrying more DNMs in ASD than other neurodevelopmental disorder) (Satterstrom et al., 2020). However, DNMs also significant contribute to ASD when removed the influence of IQ (Satterstrom et al., 2020). Third, SCZ showed the lowest degree of DNMs burden and contribution, indicating that DNMs are probably not the major genetic factor for the etiology of SCZ. Our results expand on the highly polygenic nature of previous studies which reported that common variants could account for over 30% of SCZ (Li et al., 2017c; Ripke et al., 2013), along with more than one hundred of SCZ-associated loci having been identified by GWAS (Li et al., 2017c).

We then estimated genetic association and observed significant overlaps in genes with LoF, Dmis, and Pfun DNMs among the five disorders. We found that UDD, ID and EE showed the strongest genetic similarity [(O/E) > 6], providing genetic evidence for the strong comorbidity observed among these three disorders. For example, a previous clinical investigation showed that 41.6% of patients with severe ID had symptoms of epilepsy (Robertson et al., 2015). In addition, ASD also showed relatively moderate genetic overlap with UDD, EE and ID [3 < (O/E) < 4]. Furthermore, SCZ was slightly but significantly correlated with ID, UDD and ASD [1 < (O/E) < 3]. Although we did not observe significant overlap of genes between SCZ and EE [(O/E) = 1.59, p_adj_ = 0.097], the trends suggest that they are genetically associated. We encourage further studies could investigate the associations between SCZ and EE based on larger sample size.

We totally prioritized 321 candidate genes including 35.51% (114/321) candidate genes that were not reported in previous cross-disorder studies (Coe et al., 2019; Gonzalez-Mantilla et al., 2016; Nguyen et al., 2017), which might be attributed to the integration of a larger sample size of disorders than previously and increased the statistical power. Based on literature search 54.39% (62/114) these novel candidate genes were reported associated with human brain disorders and 85.71% (12/14) of high confidence genes in tier 1 and tier 2 associated with neurodevelopmental disorders. These results highlight the advantages of integration of genetic data from different disorders with similar phenotypes. We noted that 73.52% (236/321) of candidate gene carry Pfun DNMs in at least two disorders, suggesting that DNMs with different functional effects in certain genes contributed to the differential etiology of the disorders. For example, missense mutations in *SCN2A* (Ben-Shalom et al., 2017; Wolff et al., 2017) presented as gain-of-function in early onset EE and loss-of-function in late onset EE and ASD, respectively. The mutations in *GABRB3* (Shi et al., 2019) and *GRIN2D* (XiangWei et al., 2019) also exhibited complexity of the pathological mechanisms. In addition, patients with DNMs in shared genes may exhibit wide range of phenotypes, but original studies only give priority to a certain disorder. For example, a large sequencing study used ASD as the inclusion criteria but took other comorbidities (ID/EE) into secondary consideration (Iossifov et al., 2014). It is important to recheck clinical phenotype, once a patient that was diagnosed with one NPD carrying Pfun variant in candidate gene of another NPD. It should also be noted that 21.61% (51/236) of shared genes carrying Pfun DNMs were biased to a specific disorder including 33 genes in UDD, 11 genes in EE and seven genes in ID, which consistent with recently findings (Coe et al., 2019). A recent study employed large-scale exome sequencing in ASD and identified 102 ASD-related risk genes (Satterstrom et al., 2020). The disorder bias of gene in our findings are also in agreement with this study. For example, 79.31% (23/29) of the Pfun DNMs were detected in patients with EE and was defined as one of the “seed” gene to discover the gene group that related with EE in current study (Chow et al., 2019). We observed that 26.48% (85/321) candidate genes carrying Pfun DNMs were present in only one disorder. Unique genes and biased genes suggest that a definition of “molecular endophenotypes” should be used in genetic counselling and genetic diagnosis, as “functional cellular endophenotypes” (Lago et al., 2018). Molecular endophenotypes represent a plausible strategy to summarize complex NPDs with high clinical heterogeneity, by separating them into different subtypes with similar pathogenic mechanisms based on DNMs, such as *MECP2* variants in patients with Rett syndrome (Amir et al., 1999; Tillotson et al., 2017).

The expression patterns and functional networks of candidate genes represent quantitative phenotypes and provide an in-depth view of NPDs (Li, Santpere, et al., 2018; Parikshak et al., 2013). Our results showed that candidate genes associated with NPDs presented specific spatiotemporal expression patterns in the human brain and prenatal neocortex, reflecting the wax and wane function of candidate genes at different times and in regions of the brain. We identified two spatiotemporal expression patterns of candidate genes (Fig. 2a). Genes in M1 exhibited high expression in embryonic and early-to-middle fetal periods and related to chromatin modification which associated with expression regulation. In contrast, candidate genes in M2 showed high expression during postnatal periods and related to synaptic communication (Fig. 2b). These expression pattern consistent with current study which found that genes involved in expression regulation high express during prenatal time and in neuronal communication genes high express during postnatal time (Satterstrom et al., 2020). Disruptive DNMs in synaptic communication related gene might impact on neurotransmission in ASD and other neurodevelopmental disorders, whereas chromatin modification related gene might regulate functional pathway that associate with these disorders. An example is that patients carrying disruptive mutations in chromodomain related gene *CHD8* and its target genes exhibited similarly clinical phenotypes (Beighley et al., 2020). Candidate genes for EE exhibited more specific features related to their expression pattern and functional pathways comparing to ASD, ID and UDD. More importantly, genes with Pfun DNMs shared by more disorders were more intolerant to genetic variants and were more likely to participate in chromatin and synaptic function, highlighting the core roles of shared genes and these two functional pathways in maintaining the normal function of human brain. Dysfunction of shared genes may cause the cascading paralysis of biological processes and contribute significantly to the occurrence of multiple disorders.

We acknowledge several limitations in this study. First, integrated DNMs derived from several different cohorts would increase the statistical power of candidate gene detection, but due attention should be paid to the lack of uniform quality control among different studies. Although we used synonymous DNMs as standardizations to remove confounding factors, bias maybe still be present. Second, an unbalanced sample size among NPDs might also influence the power of discoveries for each disorder. Third, we prioritized several candidate genes with strong statistical support but lack functional experiments. Fourth, both genetic and environmental factors are involved in the etiology of NPDs, and the combination of these factors should be the topic of further research to fully understand the etiology of NPDs.

This study demonstrated that DNMs play relatively strong roles in NPDs. In addition, significant genes overlap among the five NPDs has provided genetic evidence for clinical comorbidity, which consistent with previous proposed to encompass genetic data of a wide spectrum of neuropsychiatric disorders to identify candidate gene (Moreno-De-Luca et al., 2013). Genotype–phenotype correlation analysis of candidate genes prioritized in this study are required in the future to strengthen genetic evidence and further for genetic counseling and the clinical assessment of NPDs. The expression patterns and functional network offer novel insight for the shared and unique genetic mechanisms in the etiology of NPDs.

## Supplementary Information

Below is the link to the electronic supplementary material.

Supplementary file1 (DOCX 762 kb)
