# Supplementary material for: Cross-Disorder Analysis of De Novo Mutations in Neuropsychiatric Disorders
Source: J Autism Dev Disord. 2021 May 10;52(3):1299–313. doi: 10.1007/s10803-021-05031-7 (PMC8854168; doi:10.1007/s10803-021-05031-7)
Supplement: Supplementary file 1 — Supplementary file1 (DOCX 762 kb) [file 10803_2021_5031_MOESM1_ESM.docx]

**Cross-disorder analysis of de novo mutations in neuropsychiatric disorders**

**Figure S1. Odds ratio for each class of exonic DNMs in five disorders.**


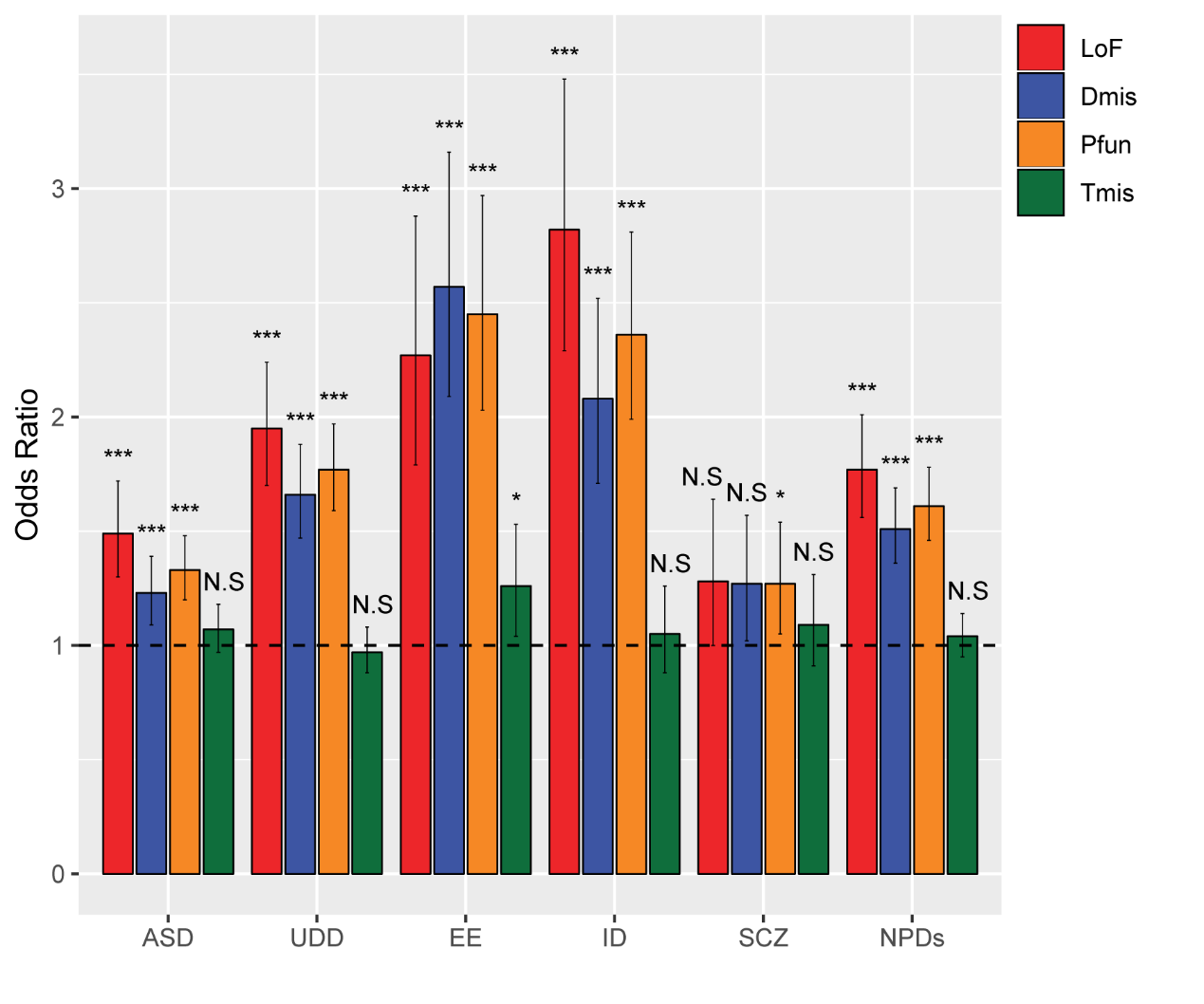


Abbreviation: ASD, autism spectrum disorder; UDD, undiagnosed developmental disorder; EE, epileptic encephalopathy; ID, intellectual disability; SCZ, schizophrenia; NPDs, integration of five disorders. Dmis, deleterious missense variants as predicted by ReVe; Tmis, tolerant missense variants as predicted by ReVe; LoF, loss-of-function variants including frameshift, stoploss and stopgain, splicing variants. Pfun, putative functional variant including Dmis and LoF variants. N.S, not significant; * P_adj_ < 0.05; ** P_adj_ < 0.01; *** P_adj_ < 0.001. Dotted black line indicates odds ratio equal to one.

**Figure S2. Comparing candidate genes in different studies.**


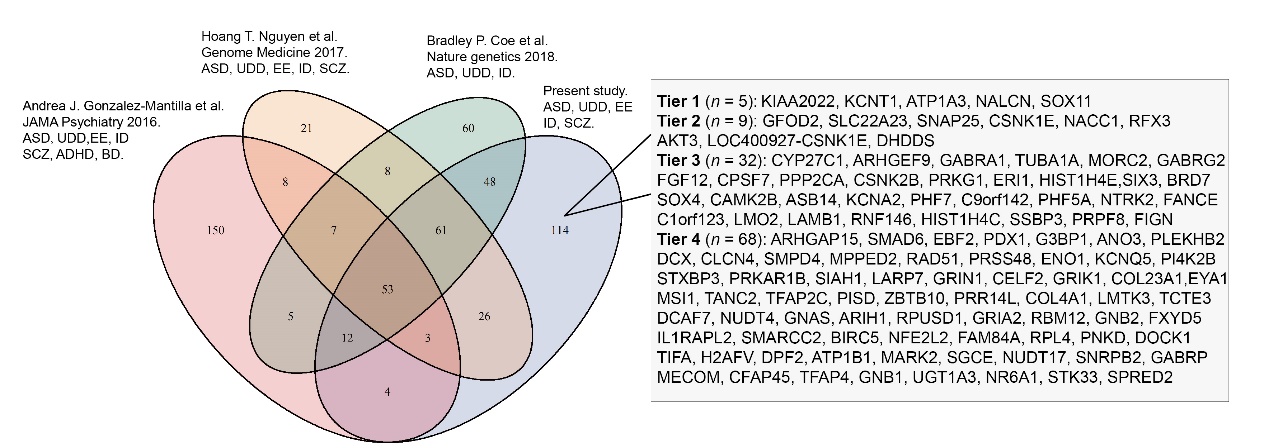


Abbreviation: ASD, autism spectrum disorder; UDD, undiagnosed developmental disorder; EE, epileptic encephalopathy; ID, intellectual disability; SCZ, schizophrenia; ADHD, attention-deficit/hyperactivity disorder; BD, bipolar disorder. Unique candidate genes with four tiers in our study in the right box.

**Table S1. Summary of studies regarding to DNMs by WES or WGS.**

| **Reference** | **PubMed ID** | **Method** | **Samples** | **DNMs** | **Exonic DNMs** |
| --- | --- | --- | --- | --- | --- |
| **Autism spectrum disorder (ASD)** | | | | | |
| Augustine Kong et al. Nature 2012 [1] | 22914163 | WGS | 78 | 4,932 | 73 |
| Jacob J. Michaelson et al. Cell 2012[2] | 23260136 | WGS | 10 | 581 | 10 |
| ASC: Silvia De Rubeis et al. Nature 2014[3] | 25363760 | WES | 1,445 | 1,702 | 1,684 |
| SSC: Ivan Iossifov et al. Nature 2014[4]  Joon-Yong An et al. Science 2018[5] | 25363768  30545852 | WES  WGS | 2,631 | 130,110 | 3,483 |
| Ryota Hashimoto et al. J Hum Genet. 2016[6] | 26582266 | WES | 30 | 38 | 35 |
| Jinchen Li, et al. Molecular Psychiatry 2017[7] | 28831199 | WGS | 32 | 2,091 | 32 |
| Rui Chen et al. Molecular Autism 2017[8] | 28344757 | WES | 116 | 128 | 121 |
| Elaine T Lim, et al. Nat Neurosci. 2017[9] | 28714951 | WES | 282 | 322 | 305 |
| Ryan KC Yuen et al. Nature Neuroscience 2017[10] | 28263302 | WGS | 1,625 | 140,556 | 2,113 |
| Atsushi Takata, et al. Cell Reports 2018[11] | 29346770 | WES | 262 | 322 | 319 |
|  |  |  | **6,511** | **280,782** | **8,175** |
| **Schizophrenia (SCZ)** | | | | | |
| Simon L Girard et al. Nat. Genet. 2011[12] | 21743468 | WES | 14 | 15 | 15 |
| Bin Xu et al. Nat. Genet. 2012[13] | 23042115 | WES | 231 | 175 | 165 |
| Suleyman Gulsuner et al. Cell 2013[14] | 23911319 | WES | 105 | 100 | 99 |
| Menachem Fromer et al. Nature 2014[15] | 24463507 | WES | 617 | 640 | 639 |
| SE McCarthy et al. Molecular Psychiatry 2014[16] | 24776741 | WES | 57 | 65 | 65 |
| Michel Guipponi et al. PLoS ONE 2014[17] | 25420024 | WES | 53 | 49 | 49 |
| Amirthagowri Ambalavanan et al. Eur J Hum Genet. 2016[18] | 26508570 | WES | 17 | 20 | 20 |
|  |  |  | **1,094** | **1,064** | **1,052** |
| **Intellectual disability (ID)** |  |  |  |  |  |
| Vissers LE et al. Nature genetics 2011[19] | 21076407 | WES | 10 | 9 | 9 |
| Anita Rauch et al. Lancet 2012[20] | 23020937 | WES | 51 | 87 | 84 |
| de Ligt J et al. N Engl J Med. 2012[21]  Gilissen C et al. Nature. 2014[22] | 23033978  24896178 | WES,  WGS | 100 | 143 | 142 |
| Fadi F. Hamdan et al. PLoS Genetics 2014[23] | 25356899 | WES | 41 | 81 | 80 |
| Stefan H Lelieveld et al. Nat Neurosci. 2016[24] | 27479843 | WES | 820 | 1,083 | 1,078 |
|  |  |  | **1,022** | **1,403** | **1,393** |
| **Epileptic encephalopathie (EE)** | | | | | |
| Veeramah KR et al. Epilepsia 2013[25] | 23647072 | WES | 10 | 15 | 15 |
| Epi4K Consortium. Nature 2013[26] | 23934111 | WES | 264 | 329 | 292 |
| Appenzeller S et al. Am J Hum Genet. 2014[27] | 25262651 | WES | 92 | 123 | 123 |
| Fadi F. Hamdan et al. Am J Hum Genet. 2017[28] | 29100083 | WGS | 197 | 294 | 293 |
| Helbig KL et al. Genetics in Medicine 2016[29] | 26795593 | WES | 216 | 302 | 292 |
| Henrike O. Heyne et al. Nature genetics 2018[30] | 29942082 | WES | 144 | 140 | 140 |
| Tran Mau-Them F et al. Genetic in medcine 2019[31] | 30166628 | WES | 10 | 10 | 10 |
|  |  |  | **933** | **1,213** | **1,165** |
| **Undiagnosed developmental disorders (UDD)** | | | | | |
| Jeremy F. McRae et al. Nature 2017[32] | 28135719 | WES | **4,293** | **8,361** | **7,696** |
| **Unaffected sibling (Control)** | | | | | |
| Bin Xu et al. Nat. Genet. 2012 [13] | 23042115 | WES | 34 | 18 | 17 |
| Suleyman Gulsuner et al. Cell 2013 [14] | 23911319 | WES | 84 | 67 | 67 |
| Genome of the Netherlands Consortium. Nat Genet. 2014[33]  Kloosterman WP et al. Genome Res 2015[34] | 24974849  25883321 | WGS | 258 | 11,311 | 141 |
|  |  |  |  |  |  |
| SSC: Ivan Iossifov et al. Nature 2014[4]  SSC: Joon-Yong An et al. Science 2018[5] | 25363768  30545852 | WES  WGS | 2151 | 126,880 | 2,663 |
| Raheleh Rahbari et al. Nature Genetics 2015[35] | 26656846 | WGS | 12 | 747 | 19 |
| Goldmann JM, et al. Nature Genetics 2016[36] | 27322544 | WGS | 832 | 35,793 | 702 |
| Ji feng Guo et al. PNAS. 2018[37] | 30348779 | WES | 20 | 20 | 20 |
|  |  |  | **3,391** | **174,836** | **3,629** |

DNMs, *de novo* mutations number reported in the original; Exonic DNMs, *de novo* mutations number annotated into exon in this study.

**Table S2. Overlap of genes across five disorders based on *de novo* mutations.**

| Disorders | **LoF** | | | **Dmis** | | | **Pfun** | | | |
| --- | --- | --- | --- | --- | --- | --- | --- | --- | --- | --- |
|  | O/E | *P* | *P*_adj_ | O/E | *P* | *P*_adj_ | O/E | *P* | *P*_adj_ |  |
| ASD vs UDD | 6.76 | 1.00E-05 | **3.85E-05** | 2.96 | 1.00E-05 | **3.85E-05** | 3.35 | 1.00E-05 | **3.85E-05** |  |
| ASD vs EE | 3.64 | 1.23E-03 | **2.46E-03** | 3.93 | 1.00E-05 | **3.85E-05** | 3.36 | 3.00E-05 | **9.37E-05** |  |
| ASD vs ID | 8.47 | 1.00E-05 | **3.85E-05** | 3.25 | 1.00E-05 | **3.85E-05** | 4.33 | 1.00E-05 | **3.85E-05** |  |
| ASD vs SCZ | 2.60 | 1.10E-04 | **2.89E-04** | 1.88 | 2.00E-05 | **6.67E-05** | 1.77 | 4.00E-05 | **1.11E-04** |  |
| UDD vs EE | 8.02 | 1.07E-03 | **2.23E-03** | 8.62 | 1.00E-05 | **3.85E-05** | 6.03 | 4.00E-05 | **1.11E-04** |  |
| UDD vs ID | 25.94 | 1.00E-05 | **3.85E-05** | 7.89 | 1.00E-05 | **3.85E-05** | 10.51 | 1.00E-05 | **3.85E-05** |  |
| UDD vs SCZ | 2.57 | 1.68E-02 | **2.79E-02** | 2.34 | 2.00E-05 | **6.67E-05** | 2.01 | 1.90E-04 | **4.52E-04** |  |
| EE vs ID | 9.11 | 1.53E-03 | **2.83E-03** | 9.82 | 1.00E-05 | **3.85E-05** | 8.21 | 1.00E-05 | **3.85E-05** |  |
| EE vs SCZ | 2.00 | 0.10 | 0.12 | 1.91 | 0.070 | 0.097 | 1.59 | 0.073 | 0.097 |  |
| ID vs SCZ | 5.12 | 3.60E-04 | **8.18E-04** | 2.37 | 3.53E-03 | **6.09E-03** | 2.48 | 1.40E-04 | **3.50E-04** |  |

Abbreviation: O/E, ratio of observed to expected numbers of shared genes. Dmis, Deleterious missense variants; Tmis, Tolerant missense variants; LoF, loss of function. LoF include frameshift, stoploss and stopgain, splicing variants. Pfun, Putative functional variants, including Dmis and LoF variants. The Benjamini and Hochberg false discovery rate (FDR) procedure was used to adjust for multiple testing. *P*_adj_ below 0.05 were highlighted in bold.

**Table S3. The TADA FDR values of genes with putative functional DNMs.**

| **Group** | **Gene** | **LoF** | **Dmis** | **P-value** | **FDR** |
| --- | --- | --- | --- | --- | --- |
| ASD | CHD8 | 10 | 4 | 8.95E-07 | 4.44E-13 |
| ASD | SCN2A | 5 | 7 | 8.95E-07 | 1.40E-10 |
| ASD | SYNGAP1 | 8 | 1 | 8.95E-07 | 4.68E-09 |
| ASD | PTEN | 3 | 6 | 8.95E-07 | 7.51E-09 |
| ASD | KDM5B | 5 | 4 | 8.95E-07 | 3.60E-08 |
| ASD | SLC6A1 | 3 | 5 | 8.95E-07 | 1.16E-07 |
| ASD | ADNP | 6 | 0 | 8.95E-07 | 1.16E-06 |
| ASD | ARID1B | 6 | 1 | 8.95E-07 | 3.13E-06 |
| ASD | SHANK3 | 6 | 0 | 8.95E-07 | 4.82E-06 |
| ASD | DYRK1A | 5 | 0 | 8.95E-07 | 1.33E-05 |
| ASD | CHD2 | 5 | 1 | 8.95E-07 | 2.26E-05 |
| ASD | GRIN2B | 4 | 2 | 8.95E-07 | 4.33E-05 |
| ASD | ANK2 | 5 | 2 | 1.79E-06 | 9.78E-05 |
| ASD | TBR1 | 2 | 3 | 1.79E-06 | 1.67E-04 |
| ASD | MYO1E | 3 | 2 | 1.79E-06 | 2.36E-04 |
| ASD | NAA15 | 4 | 0 | 1.79E-06 | 3.07E-04 |
| ASD | POGZ | 3 | 2 | 1.79E-06 | 4.03E-04 |
| ASD | PPP2R5D | 3 | 1 | 1.79E-06 | 5.46E-04 |
| ASD | DNMT3A | 1 | 4 | 3.58E-06 | 6.94E-04 |
| ASD | FOXP1 | 3 | 1 | 3.58E-06 | 8.41E-04 |
| ASD | KMT5B | 3 | 1 | 7.16E-06 | 1.02E-03 |
| ASD | TRIP12 | 3 | 2 | 7.16E-06 | 1.19E-03 |
| ASD | STXBP1 | 1 | 3 | 1.07E-05 | 1.64E-03 |
| ASD | WDFY3 | 3 | 3 | 1.07E-05 | 2.07E-03 |
| ASD | WAC | 3 | 0 | 2.33E-05 | 2.71E-03 |
| ASD | BRD7 | 3 | 0 | 2.51E-05 | 3.32E-03 |
| ASD | PIK3CA | 0 | 4 | 3.76E-05 | 4.15E-03 |
| ASD | ASB14 | 1 | 2 | 5.55E-05 | 5.29E-03 |
| ASD | NR2F1 | 2 | 1 | 7.34E-05 | 6.54E-03 |
| ASD | DSCAM | 4 | 0 | 7.34E-05 | 7.73E-03 |
| ASD | PRPF8 | 2 | 3 | 8.23E-05 | 8.92E-03 |
| ASD | KCNQ2 | 3 | 0 | 9.48E-05 | 1.02E-02 |
| ASD | TCF4 | 2 | 1 | 9.48E-05 | 1.14E-02 |
| ASD | DDX3X | 2 | 1 | 9.48E-05 | 1.25E-02 |
| ASD | PBX1 | 1 | 2 | 9.84E-05 | 1.36E-02 |
| ASD | ASH1L | 4 | 0 | 1.04E-04 | 1.48E-02 |
| ASD | GALNT18 | 2 | 1 | 1.07E-04 | 1.59E-02 |
| ASD | NRXN1 | 2 | 2 | 1.07E-04 | 1.70E-02 |
| ASD | PRKAR1B | 1 | 2 | 1.27E-04 | 1.83E-02 |
| ASD | AGO3 | 2 | 1 | 1.31E-04 | 1.95E-02 |
| ASD | GRIK1 | 2 | 1 | 1.36E-04 | 2.08E-02 |
| ASD | GIGYF1 | 3 | 0 | 1.65E-04 | 2.21E-02 |
| ASD | PTPN11 | 1 | 2 | 1.66E-04 | 2.34E-02 |
| ASD | TANC2 | 2 | 2 | 1.75E-04 | 2.47E-02 |
| ASD | RFX3 | 1 | 2 | 2.13E-04 | 2.62E-02 |
| ASD | LMTK3 | 2 | 1 | 2.13E-04 | 2.76E-02 |
| ASD | TLK2 | 1 | 2 | 2.24E-04 | 2.90E-02 |
| ASD | SCN1A | 1 | 3 | 2.65E-04 | 3.07E-02 |
| ASD | GRIA2 | 1 | 2 | 2.85E-04 | 3.24E-02 |
| ASD | FXYD5 | 2 | 0 | 2.88E-04 | 3.41E-02 |
| ASD | SET | 2 | 0 | 2.95E-04 | 3.58E-02 |
| ASD | ACHE | 1 | 2 | 3.15E-04 | 3.74E-02 |
| ASD | PAPOLG | 0 | 3 | 3.36E-04 | 3.91E-02 |
| ASD | LAMB1 | 1 | 3 | 3.40E-04 | 4.07E-02 |
| ASD | ATP1B1 | 2 | 0 | 3.90E-04 | 4.25E-02 |
| ASD | NUDT17 | 2 | 0 | 3.94E-04 | 4.43E-02 |
| ASD | MECOM | 1 | 2 | 4.01E-04 | 4.60E-02 |
| ASD | ILF2 | 2 | 0 | 4.37E-04 | 4.78E-02 |
| ASD | STK33 | 2 | 0 | 4.74E-04 | 4.97E-02 |
| ASD | FANCE | 2 | 0 | 5.15E-04 | 5.17E-02 |
| ASD | ASXL3 | 3 | 0 | 6.30E-04 | 5.95E-02 |
| ASD | TFAP2C | 2 | 0 | 6.98E-04 | 6.56E-02 |
| ASD | SPAST | 2 | 0 | 8.02E-04 | 6.99E-02 |
| ASD | CELF2 | 2 | 0 | 8.05E-04 | 7.20E-02 |
| ASD | EYA1 | 2 | 0 | 8.46E-04 | 7.61E-02 |
| ASD | UNC80 | 1 | 1 | 8.93E-04 | 8.03E-02 |
| ASD | ERI1 | 1 | 1 | 9.47E-04 | 8.45E-02 |
| ASD | TCF7L2 | 2 | 0 | 9.68E-04 | 8.65E-02 |
| ASD | MYT1L | 1 | 2 | 1.06E-03 | 9.26E-02 |
| ASD | RNF146 | 1 | 1 | 1.11E-03 | 9.87E-02 |
| ASD | CUL3 | 2 | 0 | 1.18E-03 | 1.01E-01 |
| ASD | TBL1XR1 | 1 | 1 | 1.23E-03 | 1.07E-01 |
| ASD | QRICH1 | 2 | 0 | 1.24E-03 | 1.11E-01 |
| ASD | G3BP1 | 1 | 1 | 1.57E-03 | 1.24E-01 |
| ASD | STXBP3 | 1 | 1 | 1.76E-03 | 1.32E-01 |
| ASD | GABRB3 | 1 | 1 | 1.98E-03 | 1.42E-01 |
| ASD | CNOT3 | 2 | 0 | 2.20E-03 | 1.59E-01 |
| ASD | CAMK2A | 1 | 1 | 2.44E-03 | 1.71E-01 |
| ASD | ATP1A3 | 0 | 3 | 2.48E-03 | 1.72E-01 |
| ASD | GNAI1 | 0 | 2 | 2.57E-03 | 1.76E-01 |
| ASD | CSNK2A1 | 0 | 2 | 2.93E-03 | 1.97E-01 |
| ASD | PDK2 | 0 | 2 | 3.06E-03 | 2.02E-01 |
| ASD | FBXO11 | 1 | 1 | 3.14E-03 | 2.05E-01 |
| ASD | GLRA2 | 0 | 2 | 3.19E-03 | 2.10E-01 |
| ASD | GABRG2 | 0 | 2 | 3.32E-03 | 2.14E-01 |
| ASD | MED13L | 2 | 1 | 3.37E-03 | 2.15E-01 |
| ASD | ABI2 | 0 | 2 | 3.43E-03 | 2.19E-01 |
| ASD | TFAP4 | 0 | 2 | 3.56E-03 | 2.28E-01 |
| ASD | CTCF | 1 | 1 | 3.59E-03 | 2.31E-01 |
| ASD | WDR26 | 0 | 2 | 3.66E-03 | 2.36E-01 |
| ASD | BCL11A | 2 | 0 | 4.00E-03 | 2.48E-01 |
| ASD | ANKRD11 | 3 | 1 | 4.20E-03 | 2.54E-01 |
| ASD | CSNK1E | 0 | 2 | 4.28E-03 | 2.58E-01 |
| ASD | MBD5 | 2 | 0 | 4.30E-03 | 2.60E-01 |
| ASD | MARK2 | 1 | 1 | 4.77E-03 | 2.78E-01 |
| ASD | FOXG1 | 0 | 2 | 4.82E-03 | 2.80E-01 |
| ASD | SMARCC2 | 2 | 0 | 4.84E-03 | 2.82E-01 |
| ASD | LOC400927-CSNK1E | 0 | 2 | 5.18E-03 | 2.92E-01 |
| ASD | PSD3 | 1 | 1 | 5.35E-03 | 2.98E-01 |
| ASD | FAM200A | 1 | 0 | 5.74E-03 | 3.14E-01 |
| ASD | NUDT4 | 1 | 0 | 6.46E-03 | 3.40E-01 |
| ASD | IRF2BPL | 1 | 1 | 6.71E-03 | 3.50E-01 |
| ASD | TIFA | 1 | 0 | 6.86E-03 | 3.56E-01 |
| ASD | CBL | 0 | 2 | 6.98E-03 | 3.63E-01 |
| ASD | PRR14L | 1 | 0 | 7.43E-03 | 3.82E-01 |
| ASD | TCTE3 | 1 | 0 | 7.44E-03 | 3.83E-01 |
| ASD | PRKD1 | 0 | 2 | 7.50E-03 | 3.88E-01 |
| ASD | BIRC5 | 1 | 0 | 7.74E-03 | 3.95E-01 |
| ASD | CSNK2B | 1 | 0 | 8.16E-03 | 4.16E-01 |
| ASD | TNPO3 | 0 | 2 | 8.61E-03 | 4.30E-01 |
| ASD | PHF7 | 1 | 0 | 1.12E-02 | 4.77E-01 |
| ASD | HIST1H1E | 1 | 0 | 1.14E-02 | 4.79E-01 |
| ASD | AGO1 | 0 | 2 | 1.14E-02 | 4.80E-01 |
| ASD | SMC3 | 0 | 2 | 1.16E-02 | 4.83E-01 |
| ASD | ARHGAP15 | 1 | 0 | 1.55E-02 | 5.29E-01 |
| ASD | CYP27C1 | 1 | 0 | 1.57E-02 | 5.31E-01 |
| ASD | UPF3B | 1 | 0 | 1.61E-02 | 5.36E-01 |
| ASD | H2AFV | 0 | 1 | 1.68E-02 | 5.43E-01 |
| ASD | LZTR1 | 0 | 2 | 1.73E-02 | 5.46E-01 |
| ASD | SMARCD1 | 1 | 0 | 1.76E-02 | 5.51E-01 |
| ASD | SYNCRIP | 1 | 0 | 1.86E-02 | 5.62E-01 |
| ASD | GNAS | 0 | 2 | 1.89E-02 | 5.65E-01 |
| ASD | SMARCA4 | 0 | 3 | 1.91E-02 | 5.68E-01 |
| ASD | CPSF7 | 1 | 0 | 2.00E-02 | 5.75E-01 |
| ASD | UGT1A3 | 1 | 0 | 2.04E-02 | 5.81E-01 |
| ASD | SETBP1 | 1 | 1 | 2.09E-02 | 5.85E-01 |
| ASD | PPM1D | 1 | 0 | 2.35E-02 | 6.04E-01 |
| ASD | KMT2A | 3 | 0 | 2.53E-02 | 6.15E-01 |
| ASD | NSD2 | 1 | 0 | 2.65E-02 | 6.20E-01 |
| ASD | MECP2 | 1 | 0 | 2.69E-02 | 6.23E-01 |
| ASD | PHIP | 1 | 1 | 2.74E-02 | 6.26E-01 |
| ASD | ZMYND11 | 1 | 0 | 2.81E-02 | 6.29E-01 |
| ASD | PHF21A | 1 | 0 | 2.97E-02 | 6.36E-01 |
| ASD | NACC1 | 1 | 0 | 2.99E-02 | 6.37E-01 |
| ASD | FOXP2 | 1 | 0 | 3.03E-02 | 6.38E-01 |
| ASD | SPRED2 | 1 | 0 | 3.10E-02 | 6.43E-01 |
| ASD | PPP2CA | 0 | 1 | 3.22E-02 | 6.47E-01 |
| ASD | SOX5 | 1 | 0 | 3.52E-02 | 6.58E-01 |
| ASD | MAP2K1 | 0 | 1 | 3.59E-02 | 6.59E-01 |
| ASD | CTNNB1 | 1 | 0 | 3.63E-02 | 6.61E-01 |
| ASD | DHDDS | 0 | 1 | 3.71E-02 | 6.63E-01 |
| ASD | DYNC1H1 | 2 | 3 | 3.74E-02 | 6.65E-01 |
| ASD | ENO1 | 0 | 1 | 3.75E-02 | 6.65E-01 |
| ASD | CACNA1E | 0 | 3 | 3.75E-02 | 6.66E-01 |
| ASD | YWHAG | 0 | 1 | 3.83E-02 | 6.68E-01 |
| ASD | SYT1 | 0 | 1 | 3.91E-02 | 6.71E-01 |
| ASD | GABRA1 | 0 | 1 | 4.07E-02 | 6.75E-01 |
| ASD | SMAD6 | 0 | 1 | 4.28E-02 | 6.83E-01 |
| ASD | TUBA1A | 0 | 1 | 4.46E-02 | 6.88E-01 |
| ASD | VEZF1 | 0 | 1 | 4.51E-02 | 6.89E-01 |
| ASD | RPL4 | 0 | 1 | 4.52E-02 | 6.90E-01 |
| ASD | DPF2 | 0 | 1 | 4.57E-02 | 6.91E-01 |
| ASD | GNAO1 | 0 | 1 | 4.80E-02 | 6.97E-01 |
| ASD | GNB2 | 0 | 1 | 4.80E-02 | 6.97E-01 |
| ASD | SMAD4 | 0 | 1 | 5.09E-02 | 7.05E-01 |
| ASD | GABRB2 | 0 | 1 | 5.11E-02 | 7.05E-01 |
| ASD | MEIS2 | 0 | 1 | 5.41E-02 | 7.13E-01 |
| ASD | SLC22A23 | 0 | 1 | 5.63E-02 | 7.17E-01 |
| ASD | COL4A3BP | 0 | 1 | 5.83E-02 | 7.22E-01 |
| ASD | CFAP45 | 0 | 1 | 5.99E-02 | 7.24E-01 |
| ASD | EEF1A2 | 0 | 1 | 6.14E-02 | 7.27E-01 |
| ASD | PUF60 | 0 | 1 | 6.20E-02 | 7.29E-01 |
| ASD | SOX11 | 0 | 1 | 6.35E-02 | 7.31E-01 |
| ASD | BRAF | 0 | 1 | 6.61E-02 | 7.36E-01 |
| ASD | CAMK2B | 0 | 1 | 7.37E-02 | 7.47E-01 |
| ASD | DEAF1 | 0 | 1 | 7.37E-02 | 7.47E-01 |
| ASD | NR4A2 | 0 | 1 | 7.63E-02 | 7.51E-01 |
| ASD | AHDC1 | 1 | 1 | 7.90E-02 | 7.55E-01 |
| ASD | KDM6A | 1 | 0 | 7.93E-02 | 7.56E-01 |
| ASD | NALCN | 0 | 2 | 8.03E-02 | 7.57E-01 |
| ASD | KIF5C | 0 | 1 | 8.07E-02 | 7.58E-01 |
| ASD | RBM12 | 0 | 1 | 8.25E-02 | 7.60E-01 |
| ASD | EBF3 | 0 | 1 | 8.42E-02 | 7.63E-01 |
| ASD | EP300 | 1 | 1 | 8.51E-02 | 7.64E-01 |
| ASD | SETD5 | 1 | 0 | 8.89E-02 | 7.68E-01 |
| ASD | CASK | 0 | 1 | 8.97E-02 | 7.68E-01 |
| ASD | KCNB1 | 0 | 1 | 9.11E-02 | 7.70E-01 |
| ASD | DLG4 | 0 | 1 | 9.20E-02 | 7.71E-01 |
| ASD | NSD1 | 1 | 1 | 9.34E-02 | 7.73E-01 |
| ASD | KCNQ3 | 0 | 1 | 9.40E-02 | 7.73E-01 |
| ASD | EFTUD2 | 0 | 1 | 9.62E-02 | 7.75E-01 |
| ASD | IQSEC2 | 0 | 1 | 9.76E-02 | 7.76E-01 |
| ASD | PACS1 | 0 | 1 | 9.97E-02 | 7.78E-01 |
| ASD | CHD3 | 0 | 2 | 1.08E-01 | 7.84E-01 |
| ASD | GABBR2 | 0 | 1 | 1.13E-01 | 7.86E-01 |
| ASD | HK1 | 0 | 1 | 1.15E-01 | 7.87E-01 |
| ASD | CDK13 | 0 | 1 | 1.22E-01 | 7.90E-01 |
| ASD | USP7 | 0 | 1 | 1.22E-01 | 7.91E-01 |
| ASD | KIAA2022 | 0 | 1 | 1.53E-01 | 8.02E-01 |
| ASD | SIN3A | 0 | 1 | 1.73E-01 | 8.08E-01 |
| ASD | KCNT1 | 1 | 0 | 2.51E-01 | 8.30E-01 |
| ASD | GRIN1 | 0 | 1 | 2.60E-01 | 8.32E-01 |
| ASD | TCF20 | 1 | 0 | 3.07E-01 | 8.43E-01 |
| ASD | SRCAP | 2 | 0 | 3.74E-01 | 8.55E-01 |
| ASD | BRPF1 | 0 | 1 | 3.74E-01 | 8.55E-01 |
| ASD | KAT6A | 1 | 0 | 4.56E-01 | 8.67E-01 |
| ASD | HECW2 | 0 | 1 | 5.68E-01 | 8.80E-01 |
| ASD | CREBBP | 0 | 2 | 5.72E-01 | 8.80E-01 |
| ASD | ITPR1 | 0 | 2 | 5.72E-01 | 8.80E-01 |
| ASD | CHD4 | 0 | 1 | 7.34E-01 | 8.96E-01 |
| ASD | SCN8A | 0 | 1 | 7.34E-01 | 8.96E-01 |
| ASD | USP9X | 0 | 1 | 8.37E-01 | 9.04E-01 |
| ASD | SON | 0 | 1 | 8.48E-01 | 9.05E-01 |
| EE | SCN1A | 12 | 11 | 5.03E-06 | 0 |
| EE | IRF2BPL | 10 | 0 | 5.03E-06 | 3.05E-15 |
| EE | SCN2A | 0 | 9 | 5.03E-06 | 7.78E-14 |
| EE | SCN8A | 1 | 6 | 5.03E-06 | 3.06E-10 |
| EE | DNM1 | 0 | 6 | 5.03E-06 | 1.82E-09 |
| EE | KCNQ2 | 0 | 6 | 5.03E-06 | 3.03E-09 |
| EE | CDKL5 | 3 | 3 | 5.03E-06 | 4.85E-09 |
| EE | GABRB2 | 0 | 5 | 5.03E-06 | 2.37E-08 |
| EE | STXBP1 | 1 | 4 | 5.03E-06 | 5.42E-08 |
| EE | GNAO1 | 0 | 4 | 5.03E-06 | 8.11E-07 |
| EE | CACNA1A | 0 | 4 | 5.03E-06 | 7.58E-06 |
| EE | CLTC | 2 | 2 | 5.03E-06 | 1.35E-05 |
| EE | KCNT1 | 0 | 4 | 5.03E-06 | 1.86E-05 |
| EE | MEF2C | 1 | 2 | 5.03E-06 | 5.31E-05 |
| EE | GABBR2 | 0 | 3 | 1.01E-05 | 1.34E-04 |
| EE | IQSEC2 | 3 | 0 | 1.01E-05 | 2.45E-04 |
| EE | CHD2 | 2 | 1 | 2.01E-05 | 5.78E-04 |
| EE | SNAP25 | 0 | 2 | 2.01E-05 | 1.23E-03 |
| EE | FGF12 | 0 | 2 | 2.01E-05 | 1.87E-03 |
| EE | DHDDS | 0 | 2 | 2.01E-05 | 2.56E-03 |
| EE | GABRA1 | 0 | 2 | 2.01E-05 | 3.24E-03 |
| EE | KCNA2 | 0 | 2 | 3.02E-05 | 3.92E-03 |
| EE | NTRK2 | 0 | 2 | 5.03E-05 | 4.70E-03 |
| EE | CSNK1E | 0 | 2 | 5.03E-05 | 5.44E-03 |
| EE | EEF1A2 | 0 | 2 | 6.04E-05 | 6.20E-03 |
| EE | SLC35A2 | 2 | 0 | 6.04E-05 | 6.90E-03 |
| EE | FOXG1 | 0 | 2 | 7.04E-05 | 7.58E-03 |
| EE | GABRB3 | 1 | 1 | 7.04E-05 | 8.21E-03 |
| EE | WDR45 | 2 | 0 | 9.05E-05 | 8.81E-03 |
| EE | LOC400927-CSNK1E | 0 | 2 | 9.05E-05 | 9.43E-03 |
| EE | EBF2 | 1 | 1 | 1.01E-04 | 1.01E-02 |
| EE | ANO3 | 0 | 2 | 1.21E-04 | 1.11E-02 |
| EE | CLCN4 | 0 | 2 | 1.31E-04 | 1.21E-02 |
| EE | SMPD4 | 0 | 2 | 1.51E-04 | 1.33E-02 |
| EE | ATP1A3 | 0 | 2 | 2.72E-04 | 1.55E-02 |
| EE | KIAA2022 | 2 | 0 | 2.82E-04 | 1.77E-02 |
| EE | GRIN1 | 1 | 1 | 3.22E-04 | 2.00E-02 |
| EE | GRIN2B | 0 | 2 | 3.62E-04 | 2.37E-02 |
| EE | COL4A1 | 0 | 2 | 3.82E-04 | 2.74E-02 |
| EE | SYNGAP1 | 2 | 0 | 3.82E-04 | 3.09E-02 |
| EE | TAF1 | 0 | 2 | 3.82E-04 | 3.47E-02 |
| EE | DOCK1 | 0 | 2 | 4.53E-04 | 3.96E-02 |
| EE | NUDT4 | 0 | 1 | 5.63E-04 | 4.64E-02 |
| EE | TIFA | 0 | 1 | 6.04E-04 | 5.32E-02 |
| EE | HIST1H4E | 0 | 1 | 7.14E-04 | 7.83E-02 |
| EE | ARID1B | 2 | 0 | 7.44E-04 | 9.49E-02 |
| EE | RAB11A | 0 | 1 | 7.85E-04 | 1.15E-01 |
| EE | NAA10 | 0 | 1 | 1.02E-03 | 1.55E-01 |
| EE | TCTE3 | 1 | 0 | 1.41E-03 | 1.93E-01 |
| EE | ZC4H2 | 1 | 0 | 1.46E-03 | 1.99E-01 |
| EE | RNF146 | 0 | 1 | 1.65E-03 | 2.19E-01 |
| EE | PTEN | 0 | 1 | 1.72E-03 | 2.27E-01 |
| EE | CACNA1E | 0 | 2 | 1.96E-03 | 2.48E-01 |
| EE | YWHAG | 0 | 1 | 2.34E-03 | 2.70E-01 |
| EE | CSNK2A1 | 0 | 1 | 2.51E-03 | 2.73E-01 |
| EE | TUBA1A | 0 | 1 | 3.46E-03 | 2.96E-01 |
| EE | GABRG2 | 0 | 1 | 3.53E-03 | 2.97E-01 |
| EE | RPL4 | 0 | 1 | 3.60E-03 | 2.98E-01 |
| EE | ABI2 | 0 | 1 | 3.71E-03 | 3.02E-01 |
| EE | CPSF7 | 0 | 1 | 4.76E-03 | 3.28E-01 |
| EE | ARHGEF9 | 0 | 1 | 5.01E-03 | 3.31E-01 |
| EE | G3BP1 | 1 | 0 | 5.36E-03 | 3.48E-01 |
| EE | DCX | 1 | 0 | 5.39E-03 | 3.51E-01 |
| EE | PURA | 1 | 0 | 5.62E-03 | 3.59E-01 |
| EE | SPAST | 0 | 1 | 5.84E-03 | 3.63E-01 |
| EE | COL4A3BP | 0 | 1 | 6.05E-03 | 3.66E-01 |
| EE | MECP2 | 0 | 1 | 6.78E-03 | 3.75E-01 |
| EE | NACC1 | 0 | 1 | 7.53E-03 | 3.78E-01 |
| EE | BRAF | 0 | 1 | 7.83E-03 | 3.82E-01 |
| EE | PPP2R1A | 0 | 1 | 8.26E-03 | 3.88E-01 |
| EE | RFX3 | 0 | 1 | 8.71E-03 | 3.92E-01 |
| EE | WAC | 1 | 0 | 8.84E-03 | 3.94E-01 |
| EE | DDX3X | 0 | 1 | 8.89E-03 | 3.95E-01 |
| EE | PHF21A | 1 | 0 | 9.80E-03 | 4.11E-01 |
| EE | SPRED2 | 1 | 0 | 1.01E-02 | 4.14E-01 |
| EE | KCNB1 | 0 | 1 | 1.29E-02 | 4.45E-01 |
| EE | RBM12 | 1 | 0 | 1.30E-02 | 4.47E-01 |
| EE | KCNQ3 | 0 | 1 | 1.37E-02 | 4.54E-01 |
| EE | AGO1 | 0 | 1 | 1.43E-02 | 4.59E-01 |
| EE | MARK2 | 1 | 0 | 1.45E-02 | 4.62E-01 |
| EE | CASK | 1 | 0 | 1.48E-02 | 4.63E-01 |
| EE | PSD3 | 1 | 0 | 1.55E-02 | 4.70E-01 |
| EE | LMTK3 | 1 | 0 | 1.59E-02 | 4.72E-01 |
| EE | SMC3 | 1 | 0 | 1.62E-02 | 4.75E-01 |
| EE | DNMT3A | 0 | 1 | 1.67E-02 | 4.77E-01 |
| EE | ASXL1 | 1 | 0 | 2.27E-02 | 5.12E-01 |
| EE | HECW2 | 0 | 1 | 2.30E-02 | 5.13E-01 |
| EE | PHIP | 1 | 0 | 2.36E-02 | 5.16E-01 |
| EE | GRIN2A | 1 | 0 | 2.48E-02 | 5.22E-01 |
| EE | CHD4 | 0 | 1 | 2.65E-02 | 5.29E-01 |
| EE | SON | 1 | 0 | 8.31E-02 | 5.70E-01 |
| EE | CREBBP | 0 | 1 | 4.38E-01 | 7.06E-01 |
| EE | KMT2A | 0 | 1 | 7.99E-01 | 7.76E-01 |
| EE | ANKRD11 | 1 | 0 | 8.40E-01 | 7.82E-01 |
| EE | DYNC1H1 | 0 | 1 | 9.57E-01 | 8.01E-01 |
| ID | SCN2A | 7 | 2 | 4.39E-06 | 1.71E-13 |
| ID | DDX3X | 8 | 0 | 4.39E-06 | 1.91E-13 |
| ID | POGZ | 8 | 0 | 4.39E-06 | 5.36E-13 |
| ID | ARID1B | 7 | 0 | 4.39E-06 | 1.59E-10 |
| ID | CTNNB1 | 5 | 0 | 4.39E-06 | 1.66E-08 |
| ID | ASXL3 | 5 | 0 | 4.39E-06 | 2.72E-07 |
| ID | KAT6B | 5 | 0 | 4.39E-06 | 4.99E-07 |
| ID | GATAD2B | 4 | 0 | 4.39E-06 | 9.50E-07 |
| ID | DLG4 | 3 | 1 | 4.39E-06 | 2.85E-06 |
| ID | TBR1 | 2 | 2 | 4.39E-06 | 4.78E-06 |
| ID | PPP2R5D | 0 | 4 | 4.39E-06 | 7.54E-06 |
| ID | SYNGAP1 | 4 | 0 | 4.39E-06 | 1.08E-05 |
| ID | TCF20 | 4 | 0 | 4.39E-06 | 1.68E-05 |
| ID | CHD2 | 3 | 1 | 4.39E-06 | 2.46E-05 |
| ID | PHIP | 2 | 2 | 4.39E-06 | 3.36E-05 |
| ID | WDR45 | 3 | 0 | 4.39E-06 | 4.31E-05 |
| ID | WAC | 3 | 0 | 4.39E-06 | 5.70E-05 |
| ID | PPP1CB | 1 | 2 | 4.39E-06 | 7.42E-05 |
| ID | SOX5 | 3 | 0 | 4.39E-06 | 9.02E-05 |
| ID | GRIN2A | 0 | 4 | 4.39E-06 | 1.11E-04 |
| ID | GRIN2B | 0 | 4 | 4.39E-06 | 1.32E-04 |
| ID | MED13L | 2 | 2 | 4.39E-06 | 1.50E-04 |
| ID | SCN8A | 1 | 3 | 4.39E-06 | 1.71E-04 |
| ID | TCF4 | 2 | 1 | 4.39E-06 | 1.89E-04 |
| ID | TLK2 | 2 | 1 | 4.39E-06 | 2.09E-04 |
| ID | PURA | 0 | 3 | 4.39E-06 | 2.36E-04 |
| ID | FOXG1 | 1 | 2 | 4.39E-06 | 2.61E-04 |
| ID | STXBP1 | 1 | 2 | 4.39E-06 | 2.90E-04 |
| ID | COL4A3BP | 0 | 3 | 4.39E-06 | 3.24E-04 |
| ID | SETBP1 | 3 | 0 | 8.77E-06 | 4.08E-04 |
| ID | USP7 | 1 | 2 | 8.77E-06 | 4.98E-04 |
| ID | TNPO2 | 0 | 3 | 1.75E-05 | 5.96E-04 |
| ID | KCNH1 | 0 | 3 | 1.75E-05 | 7.04E-04 |
| ID | KCNQ2 | 0 | 3 | 1.75E-05 | 8.19E-04 |
| ID | AGO2 | 0 | 3 | 1.75E-05 | 9.86E-04 |
| ID | KMT2A | 3 | 1 | 3.51E-05 | 1.27E-03 |
| ID | SYNCRIP | 2 | 0 | 3.51E-05 | 1.63E-03 |
| ID | AHDC1 | 3 | 0 | 3.51E-05 | 1.98E-03 |
| ID | PPP2CA | 1 | 1 | 4.39E-05 | 2.34E-03 |
| ID | PPM1D | 2 | 0 | 5.26E-05 | 2.72E-03 |
| ID | NAA10 | 0 | 2 | 7.02E-05 | 3.20E-03 |
| ID | TUBA1A | 1 | 1 | 7.02E-05 | 3.65E-03 |
| ID | CSNK2A1 | 0 | 2 | 1.40E-04 | 4.31E-03 |
| ID | SLC35A2 | 0 | 2 | 1.40E-04 | 4.94E-03 |
| ID | PUF60 | 1 | 1 | 1.49E-04 | 5.58E-03 |
| ID | TCF7L2 | 1 | 1 | 1.49E-04 | 6.23E-03 |
| ID | FBXO11 | 1 | 1 | 1.84E-04 | 6.97E-03 |
| ID | SLC6A1 | 1 | 1 | 1.84E-04 | 7.71E-03 |
| ID | SATB2 | 1 | 1 | 2.11E-04 | 8.44E-03 |
| ID | FOXP1 | 1 | 1 | 2.11E-04 | 9.16E-03 |
| ID | FIGN | 1 | 1 | 2.11E-04 | 9.87E-03 |
| ID | DYRK1A | 1 | 1 | 2.11E-04 | 1.06E-02 |
| ID | DEAF1 | 0 | 2 | 3.33E-04 | 1.17E-02 |
| ID | SMARCA4 | 0 | 3 | 3.42E-04 | 1.29E-02 |
| ID | KIAA2022 | 2 | 0 | 3.51E-04 | 1.41E-02 |
| ID | SETD5 | 2 | 0 | 3.95E-04 | 1.57E-02 |
| ID | KCNQ5 | 0 | 2 | 3.95E-04 | 1.72E-02 |
| ID | AGO1 | 0 | 2 | 4.91E-04 | 1.91E-02 |
| ID | ATP8A1 | 0 | 2 | 5.09E-04 | 2.14E-02 |
| ID | CACNA1A | 2 | 0 | 6.32E-04 | 2.48E-02 |
| ID | SRCAP | 3 | 0 | 6.40E-04 | 2.82E-02 |
| ID | TRIP12 | 2 | 0 | 7.11E-04 | 3.22E-02 |
| ID | EHMT1 | 2 | 0 | 7.54E-04 | 3.64E-02 |
| ID | RPL26 | 1 | 0 | 7.89E-04 | 4.05E-02 |
| ID | SNRPB2 | 1 | 0 | 8.25E-04 | 4.46E-02 |
| ID | LRRC3C | 1 | 0 | 8.51E-04 | 4.87E-02 |
| ID | SET | 1 | 0 | 8.68E-04 | 6.07E-02 |
| ID | ANKRD11 | 3 | 0 | 1.12E-03 | 9.08E-02 |
| ID | FANCE | 1 | 0 | 2.28E-03 | 1.87E-01 |
| ID | ARHGAP15 | 1 | 0 | 2.47E-03 | 1.95E-01 |
| ID | SMAD6 | 1 | 0 | 2.47E-03 | 1.97E-01 |
| ID | RAC1 | 0 | 1 | 2.49E-03 | 1.99E-01 |
| ID | KAT6A | 1 | 1 | 3.00E-03 | 2.24E-01 |
| ID | POU3F3 | 1 | 0 | 3.03E-03 | 2.26E-01 |
| ID | CHD8 | 2 | 0 | 3.67E-03 | 2.56E-01 |
| ID | USP9X | 2 | 0 | 3.87E-03 | 2.65E-01 |
| ID | ARIH1 | 1 | 0 | 3.98E-03 | 2.66E-01 |
| ID | TBL1XR1 | 0 | 1 | 4.51E-03 | 2.77E-01 |
| ID | SON | 2 | 0 | 4.54E-03 | 2.79E-01 |
| ID | DHDDS | 0 | 1 | 4.87E-03 | 2.92E-01 |
| ID | MECP2 | 1 | 0 | 5.52E-03 | 3.08E-01 |
| ID | PHF21A | 1 | 0 | 6.23E-03 | 3.34E-01 |
| ID | FOXP2 | 1 | 0 | 6.36E-03 | 3.38E-01 |
| ID | SPRED2 | 1 | 0 | 6.55E-03 | 3.43E-01 |
| ID | CHAMP1 | 1 | 0 | 6.86E-03 | 3.52E-01 |
| ID | CHD3 | 1 | 1 | 7.01E-03 | 3.57E-01 |
| ID | GNAO1 | 0 | 1 | 7.35E-03 | 3.65E-01 |
| ID | GABRB2 | 0 | 1 | 8.04E-03 | 3.78E-01 |
| ID | GABRB3 | 0 | 1 | 8.12E-03 | 3.81E-01 |
| ID | NFIX | 0 | 1 | 1.03E-02 | 4.13E-01 |
| ID | NR2F1 | 0 | 1 | 1.04E-02 | 4.14E-01 |
| ID | NSD2 | 0 | 1 | 1.04E-02 | 4.15E-01 |
| ID | EEF1A2 | 0 | 1 | 1.06E-02 | 4.20E-01 |
| ID | KIF1A | 0 | 2 | 1.09E-02 | 4.24E-01 |
| ID | KMT5B | 1 | 0 | 1.10E-02 | 4.25E-01 |
| ID | NACC1 | 0 | 1 | 1.16E-02 | 4.32E-01 |
| ID | SF1 | 0 | 1 | 1.26E-02 | 4.41E-01 |
| ID | CASK | 1 | 0 | 1.35E-02 | 4.50E-01 |
| ID | CUL3 | 0 | 1 | 1.36E-02 | 4.51E-01 |
| ID | ADNP | 1 | 0 | 1.38E-02 | 4.53E-01 |
| ID | NR4A2 | 0 | 1 | 1.47E-02 | 4.66E-01 |
| ID | HNRNPU | 1 | 0 | 1.49E-02 | 4.67E-01 |
| ID | CACNA1E | 1 | 1 | 1.54E-02 | 4.73E-01 |
| ID | SMC3 | 1 | 0 | 1.55E-02 | 4.76E-01 |
| ID | IQSEC2 | 1 | 0 | 1.57E-02 | 4.79E-01 |
| ID | KIF5C | 0 | 1 | 1.57E-02 | 4.81E-01 |
| ID | RBM12 | 0 | 1 | 1.61E-02 | 4.85E-01 |
| ID | CDK13 | 1 | 0 | 1.83E-02 | 5.05E-01 |
| ID | KCNQ3 | 0 | 1 | 1.93E-02 | 5.20E-01 |
| ID | BCL11A | 1 | 0 | 2.00E-02 | 5.24E-01 |
| ID | SMC1A | 1 | 0 | 2.00E-02 | 5.25E-01 |
| ID | DNM1 | 0 | 1 | 2.01E-02 | 5.26E-01 |
| ID | MBD5 | 1 | 0 | 2.09E-02 | 5.34E-01 |
| ID | PACS1 | 0 | 1 | 2.10E-02 | 5.36E-01 |
| ID | MYT1L | 1 | 0 | 2.15E-02 | 5.38E-01 |
| ID | SHANK3 | 1 | 0 | 2.25E-02 | 5.42E-01 |
| ID | DNMT3A | 0 | 1 | 2.30E-02 | 5.46E-01 |
| ID | CLTC | 1 | 0 | 2.49E-02 | 5.60E-01 |
| ID | CREBBP | 0 | 2 | 2.49E-02 | 5.61E-01 |
| ID | GRIN1 | 0 | 1 | 2.58E-02 | 5.68E-01 |
| ID | SMARCA2 | 0 | 1 | 4.58E-02 | 6.00E-01 |
| ID | TAF1 | 0 | 1 | 1.64E-01 | 6.67E-01 |
| ID | TANC2 | 0 | 1 | 2.38E-01 | 6.93E-01 |
| ID | EP300 | 1 | 0 | 3.50E-01 | 7.28E-01 |
| ID | ITPR1 | 0 | 1 | 7.64E-01 | 8.05E-01 |
| ID | ANK2 | 1 | 0 | 8.69E-01 | 8.16E-01 |
| ID | DYNC1H1 | 0 | 2 | 9.31E-01 | 8.22E-01 |
| SCZ | TAF13 | 2 | 0 | 3.04E-05 | 1.37E-01 |
| SCZ | PHF7 | 1 | 1 | 5.07E-05 | 1.57E-01 |
| SCZ | SSBP3 | 1 | 1 | 7.09E-05 | 1.64E-01 |
| SCZ | HIST1H1E | 1 | 0 | 1.39E-03 | 4.90E-01 |
| SCZ | H2AFV | 0 | 1 | 2.43E-03 | 5.69E-01 |
| SCZ | CELF2 | 1 | 0 | 2.85E-03 | 5.77E-01 |
| SCZ | ZMYND11 | 1 | 0 | 3.17E-03 | 5.94E-01 |
| SCZ | PBX1 | 0 | 1 | 5.64E-03 | 6.38E-01 |
| SCZ | GNAO1 | 0 | 1 | 6.49E-03 | 6.47E-01 |
| SCZ | GNB2 | 0 | 1 | 6.49E-03 | 6.48E-01 |
| SCZ | KCNQ5 | 1 | 0 | 6.53E-03 | 6.49E-01 |
| SCZ | UGT1A3 | 0 | 1 | 7.38E-03 | 6.54E-01 |
| SCZ | SLC22A23 | 0 | 1 | 7.82E-03 | 6.56E-01 |
| SCZ | MECP2 | 0 | 1 | 8.53E-03 | 6.60E-01 |
| SCZ | PUF60 | 0 | 1 | 8.69E-03 | 6.61E-01 |
| SCZ | RFX3 | 0 | 1 | 9.90E-03 | 6.64E-01 |
| SCZ | MAP4K4 | 1 | 0 | 9.95E-03 | 6.64E-01 |
| SCZ | CUL3 | 0 | 1 | 1.03E-02 | 6.65E-01 |
| SCZ | TLK2 | 0 | 1 | 1.05E-02 | 6.66E-01 |
| SCZ | SMARCC2 | 1 | 0 | 1.32E-02 | 6.73E-01 |
| SCZ | POGZ | 1 | 0 | 1.37E-02 | 6.74E-01 |
| SCZ | EFTUD2 | 0 | 1 | 1.45E-02 | 6.76E-01 |
| SCZ | BRPF1 | 1 | 0 | 1.47E-02 | 6.77E-01 |
| SCZ | SYNGAP1 | 1 | 0 | 1.60E-02 | 6.80E-01 |
| SCZ | AUTS2 | 1 | 0 | 1.66E-02 | 6.81E-01 |
| SCZ | SCN2A | 1 | 0 | 1.88E-02 | 6.86E-01 |
| SCZ | GRIN2A | 0 | 1 | 2.27E-02 | 6.91E-01 |
| SCZ | NRXN1 | 0 | 1 | 2.35E-02 | 6.93E-01 |
| SCZ | CHD8 | 1 | 0 | 2.36E-02 | 6.93E-01 |
| SCZ | NALCN | 0 | 1 | 2.43E-02 | 6.94E-01 |
| SCZ | TANC2 | 0 | 1 | 2.46E-02 | 6.96E-01 |
| SCZ | CHD4 | 0 | 1 | 2.51E-02 | 6.96E-01 |
| SCZ | AHDC1 | 0 | 1 | 2.69E-02 | 7.01E-01 |
| SCZ | DSCAM | 0 | 1 | 2.83E-02 | 7.03E-01 |
| SCZ | ITPR1 | 0 | 1 | 2.99E-02 | 7.06E-01 |
| UDD | MECP2 | 6 | 8 | 9.97E-07 | 0 |
| UDD | SETD5 | 15 | 2 | 9.97E-07 | 0 |
| UDD | ASXL3 | 14 | 0 | 9.97E-07 | 0 |
| UDD | MED13L | 13 | 6 | 9.97E-07 | 0 |
| UDD | DYRK1A | 14 | 4 | 9.97E-07 | 0 |
| UDD | KCNQ2 | 0 | 16 | 9.97E-07 | 0 |
| UDD | ADNP | 19 | 1 | 9.97E-07 | 0 |
| UDD | KMT2A | 26 | 2 | 9.97E-07 | 0 |
| UDD | SYNGAP1 | 15 | 0 | 9.97E-07 | 0 |
| UDD | ANKRD11 | 32 | 0 | 9.97E-07 | 0 |
| UDD | DDX3X | 14 | 11 | 9.97E-07 | 0 |
| UDD | SCN2A | 5 | 12 | 9.97E-07 | 0 |
| UDD | ARID1B | 31 | 1 | 9.97E-07 | 0 |
| UDD | SATB2 | 9 | 4 | 9.97E-07 | 0 |
| UDD | EP300 | 12 | 4 | 9.97E-07 | 0 |
| UDD | TCF4 | 10 | 2 | 9.97E-07 | 1.39E-17 |
| UDD | FOXP1 | 8 | 4 | 9.97E-07 | 5.22E-17 |
| UDD | PPP2R5D | 0 | 12 | 9.97E-07 | 1.54E-16 |
| UDD | CTNNB1 | 11 | 0 | 9.97E-07 | 2.98E-16 |
| UDD | CDK13 | 1 | 11 | 9.97E-07 | 1.84E-15 |
| UDD | STXBP1 | 5 | 6 | 9.97E-07 | 3.90E-15 |
| UDD | PURA | 7 | 3 | 9.97E-07 | 1.02E-14 |
| UDD | TBL1XR1 | 4 | 6 | 9.97E-07 | 2.03E-14 |
| UDD | SMC1A | 8 | 1 | 9.97E-07 | 4.98E-12 |
| UDD | WDR45 | 6 | 2 | 9.97E-07 | 9.89E-12 |
| UDD | CASK | 4 | 5 | 9.97E-07 | 1.50E-11 |
| UDD | HDAC8 | 3 | 5 | 9.97E-07 | 2.03E-11 |
| UDD | KAT6A | 8 | 2 | 9.97E-07 | 2.69E-11 |
| UDD | SMARCA2 | 2 | 8 | 9.97E-07 | 3.78E-11 |
| UDD | MEF2C | 4 | 4 | 9.97E-07 | 4.92E-11 |
| UDD | CSNK2A1 | 0 | 8 | 9.97E-07 | 6.46E-11 |
| UDD | KANSL1 | 8 | 0 | 9.97E-07 | 1.21E-10 |
| UDD | SLC6A1 | 2 | 6 | 9.97E-07 | 1.81E-10 |
| UDD | NSD1 | 7 | 3 | 9.97E-07 | 2.54E-10 |
| UDD | EHMT1 | 7 | 2 | 9.97E-07 | 3.63E-10 |
| UDD | GATAD2B | 7 | 0 | 9.97E-07 | 4.70E-10 |
| UDD | KAT6B | 8 | 1 | 9.97E-07 | 5.75E-10 |
| UDD | PACS1 | 0 | 8 | 9.97E-07 | 9.99E-10 |
| UDD | GNAO1 | 2 | 5 | 9.97E-07 | 1.42E-09 |
| UDD | HNRNPU | 7 | 0 | 9.97E-07 | 2.14E-09 |
| UDD | CREBBP | 3 | 7 | 9.97E-07 | 3.79E-09 |
| UDD | CHD2 | 6 | 2 | 9.97E-07 | 5.78E-09 |
| UDD | CTCF | 0 | 7 | 9.97E-07 | 8.16E-09 |
| UDD | CNOT3 | 2 | 5 | 9.97E-07 | 1.22E-08 |
| UDD | IQSEC2 | 3 | 4 | 9.97E-07 | 1.68E-08 |
| UDD | AHDC1 | 8 | 0 | 9.97E-07 | 2.20E-08 |
| UDD | NFIX | 4 | 2 | 9.97E-07 | 3.09E-08 |
| UDD | EEF1A2 | 0 | 6 | 9.97E-07 | 5.93E-08 |
| UDD | PTPN11 | 0 | 6 | 9.97E-07 | 8.80E-08 |
| UDD | BRAF | 0 | 6 | 9.97E-07 | 1.22E-07 |
| UDD | KIF1A | 0 | 8 | 9.97E-07 | 1.83E-07 |
| UDD | CHD3 | 1 | 7 | 9.97E-07 | 2.51E-07 |
| UDD | EFTUD2 | 3 | 3 | 9.97E-07 | 3.40E-07 |
| UDD | PPM1D | 5 | 0 | 9.97E-07 | 4.33E-07 |
| UDD | NAA10 | 0 | 5 | 9.97E-07 | 5.35E-07 |
| UDD | CDKL5 | 2 | 4 | 9.97E-07 | 6.39E-07 |
| UDD | POGZ | 6 | 0 | 9.97E-07 | 7.59E-07 |
| UDD | GNAI1 | 1 | 4 | 9.97E-07 | 8.82E-07 |
| UDD | HECW2 | 1 | 6 | 9.97E-07 | 1.01E-06 |
| UDD | CHAMP1 | 5 | 0 | 9.97E-07 | 1.14E-06 |
| UDD | ZBTB18 | 4 | 1 | 9.97E-07 | 1.30E-06 |
| UDD | BCL11A | 3 | 3 | 9.97E-07 | 1.50E-06 |
| UDD | FOXG1 | 3 | 2 | 9.97E-07 | 1.78E-06 |
| UDD | CNKSR2 | 5 | 0 | 9.97E-07 | 2.21E-06 |
| UDD | USP9X | 5 | 2 | 9.97E-07 | 2.68E-06 |
| UDD | CHD4 | 1 | 6 | 9.97E-07 | 3.15E-06 |
| UDD | SCN8A | 0 | 7 | 9.97E-07 | 3.70E-06 |
| UDD | ITPR1 | 0 | 8 | 9.97E-07 | 4.72E-06 |
| UDD | BTF3 | 0 | 4 | 9.97E-07 | 5.92E-06 |
| UDD | MSL3 | 4 | 0 | 9.97E-07 | 7.18E-06 |
| UDD | UPF3B | 4 | 0 | 9.97E-07 | 8.62E-06 |
| UDD | PDHA1 | 3 | 1 | 9.97E-07 | 1.09E-05 |
| UDD | PPP1CB | 0 | 4 | 9.97E-07 | 1.34E-05 |
| UDD | MAP4K4 | 2 | 3 | 9.97E-07 | 1.68E-05 |
| UDD | DNM1 | 0 | 5 | 9.97E-07 | 2.06E-05 |
| UDD | GRIN2B | 0 | 6 | 1.99E-06 | 2.48E-05 |
| UDD | WAC | 3 | 1 | 1.99E-06 | 2.91E-05 |
| UDD | KCNH1 | 0 | 5 | 1.99E-06 | 3.33E-05 |
| UDD | KDM6A | 2 | 3 | 3.99E-06 | 3.76E-05 |
| UDD | PUF60 | 3 | 1 | 3.99E-06 | 4.22E-05 |
| UDD | NALCN | 2 | 4 | 3.99E-06 | 4.69E-05 |
| UDD | SMAD4 | 0 | 4 | 3.99E-06 | 5.39E-05 |
| UDD | COL4A3BP | 0 | 4 | 3.99E-06 | 6.39E-05 |
| UDD | SOX11 | 1 | 3 | 3.99E-06 | 7.37E-05 |
| UDD | CLTC | 3 | 2 | 3.99E-06 | 8.59E-05 |
| UDD | ZC4H2 | 3 | 0 | 3.99E-06 | 9.85E-05 |
| UDD | SET | 3 | 0 | 3.99E-06 | 1.12E-04 |
| UDD | EBF3 | 3 | 1 | 3.99E-06 | 1.25E-04 |
| UDD | PPP2R1A | 0 | 4 | 5.98E-06 | 1.39E-04 |
| UDD | GFOD2 | 1 | 2 | 5.98E-06 | 1.54E-04 |
| UDD | TCF20 | 5 | 0 | 5.98E-06 | 1.73E-04 |
| UDD | RAB11A | 1 | 2 | 7.98E-06 | 2.01E-04 |
| UDD | TAOK1 | 1 | 3 | 9.97E-06 | 2.40E-04 |
| UDD | RAC1 | 0 | 3 | 9.97E-06 | 2.78E-04 |
| UDD | KCNB1 | 1 | 3 | 9.97E-06 | 3.17E-04 |
| UDD | SMARCA4 | 0 | 6 | 9.97E-06 | 3.56E-04 |
| UDD | UNC80 | 0 | 3 | 9.97E-06 | 3.95E-04 |
| UDD | TRIP12 | 3 | 2 | 1.20E-05 | 4.41E-04 |
| UDD | PRKAR1A | 2 | 1 | 1.20E-05 | 4.86E-04 |
| UDD | SMC3 | 1 | 3 | 1.40E-05 | 5.40E-04 |
| UDD | KCNQ3 | 0 | 4 | 1.40E-05 | 5.97E-04 |
| UDD | SYT1 | 1 | 2 | 1.40E-05 | 6.57E-04 |
| UDD | AKT3 | 1 | 2 | 1.40E-05 | 7.18E-04 |
| UDD | SLC12A2 | 1 | 3 | 1.40E-05 | 7.81E-04 |
| UDD | CACNA1E | 2 | 4 | 1.40E-05 | 8.46E-04 |
| UDD | HNRNPK | 2 | 1 | 1.40E-05 | 9.10E-04 |
| UDD | SIN3A | 3 | 1 | 1.40E-05 | 9.74E-04 |
| UDD | CACNA1A | 0 | 5 | 1.40E-05 | 1.04E-03 |
| UDD | WDR26 | 2 | 1 | 1.40E-05 | 1.10E-03 |
| UDD | TAB2 | 3 | 0 | 1.40E-05 | 1.16E-03 |
| UDD | NSD2 | 3 | 0 | 1.99E-05 | 1.23E-03 |
| UDD | ARHGEF9 | 2 | 1 | 1.99E-05 | 1.30E-03 |
| UDD | HK1 | 1 | 3 | 1.99E-05 | 1.37E-03 |
| UDD | POU3F3 | 1 | 2 | 2.39E-05 | 1.45E-03 |
| UDD | DNMT3A | 1 | 3 | 2.39E-05 | 1.53E-03 |
| UDD | CYP27C1 | 0 | 3 | 2.39E-05 | 1.61E-03 |
| UDD | MORC2 | 0 | 4 | 2.39E-05 | 1.69E-03 |
| UDD | CAMK2A | 2 | 1 | 3.39E-05 | 1.78E-03 |
| UDD | BRPF1 | 4 | 0 | 3.59E-05 | 1.88E-03 |
| UDD | CUL3 | 3 | 0 | 3.79E-05 | 1.99E-03 |
| UDD | TCF12 | 2 | 1 | 3.79E-05 | 2.10E-03 |
| UDD | MYT1L | 2 | 2 | 4.19E-05 | 2.21E-03 |
| UDD | FOXP2 | 2 | 1 | 4.39E-05 | 2.32E-03 |
| UDD | QRICH1 | 3 | 0 | 4.39E-05 | 2.43E-03 |
| UDD | SF1 | 2 | 1 | 5.39E-05 | 2.56E-03 |
| UDD | ASXL1 | 4 | 0 | 5.39E-05 | 2.70E-03 |
| UDD | PRKG1 | 1 | 2 | 5.39E-05 | 2.83E-03 |
| UDD | SLC22A23 | 0 | 3 | 5.59E-05 | 2.97E-03 |
| UDD | FBXO11 | 2 | 1 | 6.38E-05 | 3.11E-03 |
| UDD | SIX3 | 0 | 3 | 6.58E-05 | 3.26E-03 |
| UDD | SOX4 | 0 | 3 | 6.98E-05 | 3.42E-03 |
| UDD | CAMK2B | 2 | 1 | 7.18E-05 | 3.58E-03 |
| UDD | AUTS2 | 4 | 0 | 7.38E-05 | 3.74E-03 |
| UDD | ZMYND11 | 0 | 3 | 7.38E-05 | 3.91E-03 |
| UDD | SOX5 | 1 | 2 | 7.78E-05 | 4.11E-03 |
| UDD | C9orf142 | 2 | 0 | 8.58E-05 | 4.35E-03 |
| UDD | PHF5A | 1 | 1 | 9.18E-05 | 4.61E-03 |
| UDD | TLK2 | 0 | 3 | 9.97E-05 | 4.89E-03 |
| UDD | C1orf123 | 1 | 1 | 1.18E-04 | 5.22E-03 |
| UDD | LMO2 | 1 | 1 | 1.26E-04 | 5.57E-03 |
| UDD | CBL | 1 | 2 | 1.28E-04 | 5.93E-03 |
| UDD | VAMP2 | 0 | 2 | 1.30E-04 | 6.30E-03 |
| UDD | TNPO3 | 2 | 1 | 1.30E-04 | 6.66E-03 |
| UDD | PLAC8L1 | 0 | 2 | 1.42E-04 | 7.08E-03 |
| UDD | CSNK2B | 1 | 1 | 1.46E-04 | 7.49E-03 |
| UDD | SNAP25 | 1 | 1 | 1.72E-04 | 7.94E-03 |
| UDD | HIST1H4C | 0 | 2 | 1.74E-04 | 8.39E-03 |
| UDD | SNX11 | 1 | 1 | 1.74E-04 | 8.84E-03 |
| UDD | HIST1H4E | 0 | 2 | 1.78E-04 | 9.29E-03 |
| UDD | HIST1H1E | 2 | 0 | 1.78E-04 | 9.73E-03 |
| UDD | PDX1 | 1 | 1 | 1.93E-04 | 1.02E-02 |
| UDD | PRKD1 | 0 | 3 | 2.19E-04 | 1.07E-02 |
| UDD | PLEKHB2 | 1 | 1 | 2.31E-04 | 1.12E-02 |
| UDD | HIST1H2AC | 0 | 2 | 2.51E-04 | 1.17E-02 |
| UDD | FAM104A | 0 | 2 | 2.61E-04 | 1.23E-02 |
| UDD | PIK3CA | 0 | 3 | 2.77E-04 | 1.28E-02 |
| UDD | MPPED2 | 0 | 2 | 3.07E-04 | 1.34E-02 |
| UDD | GOLPH3 | 1 | 1 | 3.15E-04 | 1.40E-02 |
| UDD | PTEN | 1 | 1 | 3.35E-04 | 1.46E-02 |
| UDD | RAD51 | 0 | 2 | 3.79E-04 | 1.53E-02 |
| UDD | FOSL2 | 2 | 0 | 3.85E-04 | 1.59E-02 |
| UDD | KIAA2022 | 3 | 0 | 4.19E-04 | 1.66E-02 |
| UDD | PI4K2B | 0 | 2 | 4.23E-04 | 1.73E-02 |
| UDD | PRSS48 | 0 | 2 | 4.23E-04 | 1.79E-02 |
| UDD | SIAH1 | 0 | 2 | 4.23E-04 | 1.86E-02 |
| UDD | LARP7 | 2 | 0 | 4.31E-04 | 1.92E-02 |
| UDD | EIF4A2 | 1 | 1 | 4.33E-04 | 1.99E-02 |
| UDD | VEZF1 | 2 | 0 | 4.35E-04 | 2.06E-02 |
| UDD | COL23A1 | 2 | 0 | 5.55E-04 | 2.13E-02 |
| UDD | RAB11B | 0 | 2 | 5.59E-04 | 2.21E-02 |
| UDD | MSI1 | 1 | 1 | 5.78E-04 | 2.29E-02 |
| UDD | MBD5 | 3 | 0 | 5.84E-04 | 2.37E-02 |
| UDD | SMPD2 | 1 | 1 | 5.94E-04 | 2.45E-02 |
| UDD | MAP2K1 | 0 | 2 | 5.98E-04 | 2.52E-02 |
| UDD | PISD | 2 | 0 | 6.08E-04 | 2.60E-02 |
| UDD | ZBTB10 | 2 | 0 | 6.20E-04 | 2.68E-02 |
| UDD | DCX | 1 | 1 | 6.42E-04 | 2.76E-02 |
| UDD | DCAF7 | 0 | 2 | 6.52E-04 | 2.84E-02 |
| UDD | ENO1 | 0 | 2 | 6.68E-04 | 2.93E-02 |
| UDD | NONO | 1 | 1 | 6.80E-04 | 3.01E-02 |
| UDD | YWHAG | 0 | 2 | 6.96E-04 | 3.09E-02 |
| UDD | RPUSD1 | 1 | 1 | 6.98E-04 | 3.17E-02 |
| UDD | MEIS2 | 2 | 0 | 7.26E-04 | 3.26E-02 |
| UDD | TMEM26 | 0 | 2 | 7.30E-04 | 3.34E-02 |
| UDD | IL1RAPL2 | 2 | 0 | 7.36E-04 | 3.43E-02 |
| UDD | SMARCD1 | 1 | 1 | 7.50E-04 | 3.51E-02 |
| UDD | TGFB2 | 1 | 1 | 7.68E-04 | 3.60E-02 |
| UDD | NFE2L2 | 1 | 1 | 7.78E-04 | 3.68E-02 |
| UDD | LZTR1 | 1 | 2 | 7.84E-04 | 3.77E-02 |
| UDD | FAM84A | 1 | 1 | 8.14E-04 | 3.86E-02 |
| UDD | PNKD | 0 | 2 | 8.16E-04 | 3.94E-02 |
| UDD | PBX1 | 0 | 2 | 8.40E-04 | 4.03E-02 |
| UDD | ERBB4 | 2 | 1 | 8.54E-04 | 4.12E-02 |
| UDD | CLDN5 | 0 | 2 | 8.86E-04 | 4.21E-02 |
| UDD | SHANK3 | 3 | 0 | 8.86E-04 | 4.30E-02 |
| UDD | SGCE | 0 | 2 | 9.08E-04 | 4.39E-02 |
| UDD | ACTC1 | 0 | 2 | 9.24E-04 | 4.48E-02 |
| UDD | GABRP | 0 | 2 | 9.24E-04 | 4.57E-02 |
| UDD | CFAP45 | 2 | 0 | 9.50E-04 | 4.66E-02 |
| UDD | GNB1 | 0 | 2 | 9.71E-04 | 4.76E-02 |
| UDD | DPF2 | 0 | 2 | 9.87E-04 | 4.85E-02 |
| UDD | NR6A1 | 0 | 2 | 9.87E-04 | 4.95E-02 |
| UDD | ARIH1 | 1 | 1 | 1.02E-03 | 5.04E-02 |
| UDD | GNAS | 0 | 3 | 1.18E-03 | 5.75E-02 |
| UDD | CPSF7 | 0 | 2 | 1.24E-03 | 6.07E-02 |
| UDD | GABRB2 | 0 | 2 | 1.24E-03 | 6.18E-02 |
| UDD | GABRB3 | 0 | 2 | 1.25E-03 | 6.39E-02 |
| UDD | NR2F1 | 1 | 1 | 1.36E-03 | 6.93E-02 |
| UDD | NAA15 | 2 | 0 | 1.39E-03 | 7.04E-02 |
| UDD | TCF7L2 | 1 | 1 | 1.51E-03 | 7.73E-02 |
| UDD | KDM5B | 3 | 0 | 1.63E-03 | 8.35E-02 |
| UDD | NACC1 | 0 | 2 | 1.92E-03 | 9.41E-02 |
| UDD | FAM200A | 1 | 0 | 1.94E-03 | 9.69E-02 |
| UDD | KMT5B | 2 | 0 | 2.19E-03 | 1.13E-01 |
| UDD | ATP1A3 | 0 | 3 | 2.21E-03 | 1.15E-01 |
| UDD | NR4A2 | 1 | 1 | 2.26E-03 | 1.21E-01 |
| UDD | RPL26 | 1 | 0 | 2.79E-03 | 1.58E-01 |
| UDD | PRR14L | 1 | 0 | 3.23E-03 | 1.80E-01 |
| UDD | BIRC5 | 1 | 0 | 3.64E-03 | 1.93E-01 |
| UDD | KIF5C | 0 | 2 | 3.95E-03 | 2.09E-01 |
| UDD | ERI1 | 1 | 0 | 5.32E-03 | 2.62E-01 |
| UDD | TNPO2 | 1 | 1 | 8.55E-03 | 3.57E-01 |
| UDD | FGF12 | 0 | 1 | 9.94E-03 | 3.90E-01 |
| UDD | SRCAP | 4 | 1 | 1.07E-02 | 4.07E-01 |
| UDD | SSBP3 | 1 | 0 | 1.22E-02 | 4.34E-01 |
| UDD | SLC35A2 | 1 | 0 | 1.26E-02 | 4.40E-01 |
| UDD | AGO1 | 0 | 2 | 1.34E-02 | 4.57E-01 |
| UDD | SMAD6 | 1 | 0 | 1.47E-02 | 4.75E-01 |
| UDD | LAMB1 | 2 | 1 | 1.58E-02 | 4.86E-01 |
| UDD | IRF2BPL | 1 | 1 | 1.60E-02 | 4.89E-01 |
| UDD | STXBP3 | 1 | 0 | 1.81E-02 | 5.11E-01 |
| UDD | GNB2 | 1 | 0 | 1.88E-02 | 5.18E-01 |
| UDD | GABRA1 | 0 | 1 | 1.93E-02 | 5.19E-01 |
| UDD | SYNCRIP | 1 | 0 | 1.95E-02 | 5.21E-01 |
| UDD | PDK2 | 0 | 1 | 1.99E-02 | 5.25E-01 |
| UDD | ARHGAP15 | 0 | 1 | 2.12E-02 | 5.36E-01 |
| UDD | GLRA2 | 0 | 1 | 2.14E-02 | 5.39E-01 |
| UDD | GABBR2 | 0 | 2 | 2.21E-02 | 5.45E-01 |
| UDD | GABRG2 | 0 | 1 | 2.31E-02 | 5.55E-01 |
| UDD | RPL4 | 0 | 1 | 2.35E-02 | 5.60E-01 |
| UDD | TFAP4 | 0 | 1 | 2.54E-02 | 5.76E-01 |
| UDD | EYA1 | 1 | 0 | 2.78E-02 | 5.92E-01 |
| UDD | TFAP2C | 0 | 1 | 2.90E-02 | 6.01E-01 |
| UDD | UGT1A3 | 0 | 1 | 2.90E-02 | 6.02E-01 |
| UDD | NTRK2 | 0 | 1 | 3.22E-02 | 6.19E-01 |
| UDD | SPAST | 0 | 1 | 3.36E-02 | 6.28E-01 |
| UDD | CSNK1E | 0 | 1 | 3.41E-02 | 6.32E-01 |
| UDD | CHD8 | 3 | 0 | 3.48E-02 | 6.35E-01 |
| UDD | SON | 3 | 0 | 3.90E-02 | 6.53E-01 |
| UDD | SMARCC2 | 0 | 2 | 4.11E-02 | 6.61E-01 |
| UDD | FIGN | 1 | 0 | 4.28E-02 | 6.68E-01 |
| UDD | LOC400927-CSNK1E | 0 | 1 | 4.44E-02 | 6.72E-01 |
| UDD | DEAF1 | 0 | 1 | 5.31E-02 | 6.90E-01 |
| UDD | PHIP | 2 | 0 | 5.70E-02 | 6.97E-01 |
| UDD | SETBP1 | 1 | 1 | 5.79E-02 | 6.98E-01 |
| UDD | RBM12 | 0 | 1 | 8.85E-02 | 7.24E-01 |
| UDD | AGO3 | 0 | 1 | 1.02E-01 | 7.34E-01 |
| UDD | DLG4 | 1 | 0 | 1.10E-01 | 7.41E-01 |
| UDD | GRIK1 | 0 | 1 | 1.10E-01 | 7.41E-01 |
| UDD | PSD3 | 1 | 0 | 1.29E-01 | 7.54E-01 |
| UDD | MARK2 | 0 | 1 | 1.42E-01 | 7.62E-01 |
| UDD | ANO3 | 0 | 1 | 1.42E-01 | 7.62E-01 |
| UDD | MECOM | 0 | 1 | 1.64E-01 | 7.75E-01 |
| UDD | DYNC1H1 | 0 | 7 | 1.82E-01 | 7.84E-01 |
| UDD | CLCN4 | 0 | 1 | 1.85E-01 | 7.86E-01 |
| UDD | NRXN1 | 0 | 2 | 2.75E-01 | 8.19E-01 |
| UDD | COL4A1 | 0 | 2 | 2.90E-01 | 8.24E-01 |
| UDD | SCN1A | 0 | 2 | 3.54E-01 | 8.40E-01 |
| UDD | TAF1 | 0 | 2 | 3.63E-01 | 8.41E-01 |
| UDD | GRIN1 | 0 | 1 | 5.40E-01 | 8.71E-01 |
| UDD | ASH1L | 2 | 0 | 7.39E-01 | 8.96E-01 |
| UDD | GRIN2A | 0 | 1 | 7.45E-01 | 8.96E-01 |
| UDD | PRPF8 | 0 | 2 | 8.20E-01 | 9.03E-01 |
| UDD | DOCK1 | 0 | 1 | 8.50E-01 | 9.06E-01 |
| UDD | DSCAM | 0 | 1 | 9.16E-01 | 9.11E-01 |
| UDD | ANK2 | 0 | 1 | 9.79E-01 | 9.15E-01 |
| NPDs | MECP2 | 8 | 10 | 5.27E-07 | 0 |
| NPDs | SETD5 | 18 | 2 | 5.27E-07 | 0 |
| NPDs | ASXL3 | 22 | 0 | 5.27E-07 | 0 |
| NPDs | MED13L | 17 | 9 | 5.27E-07 | 0 |
| NPDs | SCN1A | 13 | 16 | 5.27E-07 | 0 |
| NPDs | DYRK1A | 20 | 5 | 5.27E-07 | 0 |
| NPDs | KCNQ2 | 3 | 25 | 5.27E-07 | 0 |
| NPDs | ADNP | 26 | 1 | 5.27E-07 | 0 |
| NPDs | KMT2A | 32 | 4 | 5.27E-07 | 0 |
| NPDs | SYNGAP1 | 30 | 1 | 5.27E-07 | 0 |
| NPDs | ANKRD11 | 39 | 1 | 5.27E-07 | 0 |
| NPDs | DDX3X | 24 | 13 | 5.27E-07 | 0 |
| NPDs | SCN2A | 18 | 30 | 5.27E-07 | 0 |
| NPDs | ARID1B | 46 | 2 | 5.27E-07 | 0 |
| NPDs | TCF4 | 14 | 4 | 5.27E-07 | 0 |
| NPDs | FOXP1 | 12 | 6 | 5.27E-07 | 0 |
| NPDs | PPP2R5D | 3 | 17 | 5.27E-07 | 0 |
| NPDs | CTNNB1 | 17 | 0 | 5.27E-07 | 0 |
| NPDs | STXBP1 | 8 | 15 | 5.27E-07 | 0 |
| NPDs | CHD8 | 16 | 4 | 5.27E-07 | 0 |
| NPDs | POGZ | 18 | 2 | 5.27E-07 | 0 |
| NPDs | SLC6A1 | 6 | 12 | 5.27E-07 | 0 |
| NPDs | CHD2 | 16 | 5 | 5.27E-07 | 0 |
| NPDs | SATB2 | 10 | 5 | 5.27E-07 | 4.63E-17 |
| NPDs | EP300 | 14 | 5 | 5.27E-07 | 9.77E-17 |
| NPDs | SCN8A | 2 | 17 | 5.27E-07 | 1.71E-16 |
| NPDs | PURA | 8 | 6 | 5.27E-07 | 2.75E-16 |
| NPDs | WDR45 | 11 | 2 | 5.27E-07 | 4.92E-16 |
| NPDs | GRIN2B | 4 | 14 | 5.27E-07 | 7.24E-16 |
| NPDs | GNAO1 | 2 | 12 | 5.27E-07 | 1.00E-15 |
| NPDs | TBL1XR1 | 5 | 8 | 5.27E-07 | 2.59E-15 |
| NPDs | CSNK2A1 | 0 | 13 | 5.27E-07 | 4.27E-15 |
| NPDs | IRF2BPL | 12 | 2 | 5.27E-07 | 7.15E-15 |
| NPDs | PTEN | 4 | 8 | 5.27E-07 | 3.23E-14 |
| NPDs | GATAD2B | 11 | 0 | 5.27E-07 | 1.32E-13 |
| NPDs | CDK13 | 2 | 12 | 5.27E-07 | 2.40E-13 |
| NPDs | WAC | 10 | 1 | 5.27E-07 | 4.51E-13 |
| NPDs | FOXG1 | 4 | 8 | 5.27E-07 | 8.08E-13 |
| NPDs | KAT6B | 13 | 1 | 5.27E-07 | 1.33E-12 |
| NPDs | MEF2C | 5 | 6 | 5.27E-07 | 2.54E-12 |
| NPDs | CASK | 6 | 6 | 5.27E-07 | 4.98E-12 |
| NPDs | IQSEC2 | 7 | 5 | 5.27E-07 | 8.78E-12 |
| NPDs | CDKL5 | 5 | 7 | 5.27E-07 | 1.45E-11 |
| NPDs | AHDC1 | 12 | 2 | 5.27E-07 | 2.01E-11 |
| NPDs | DNM1 | 0 | 12 | 5.27E-07 | 2.76E-11 |
| NPDs | KAT6A | 10 | 3 | 5.27E-07 | 8.46E-11 |
| NPDs | EEF1A2 | 0 | 10 | 5.27E-07 | 1.60E-10 |
| NPDs | KDM5B | 8 | 4 | 5.27E-07 | 2.45E-10 |
| NPDs | TLK2 | 3 | 7 | 5.27E-07 | 4.33E-10 |
| NPDs | DNMT3A | 2 | 9 | 5.27E-07 | 8.39E-10 |
| NPDs | SHANK3 | 10 | 0 | 5.27E-07 | 1.33E-09 |
| NPDs | SMC1A | 9 | 1 | 5.27E-07 | 1.82E-09 |
| NPDs | GABRB2 | 0 | 9 | 5.27E-07 | 2.31E-09 |
| NPDs | TRIP12 | 8 | 4 | 5.27E-07 | 2.86E-09 |
| NPDs | PPM1D | 8 | 0 | 5.27E-07 | 3.56E-09 |
| NPDs | HDAC8 | 3 | 5 | 5.27E-07 | 4.36E-09 |
| NPDs | NAA10 | 0 | 8 | 5.27E-07 | 5.16E-09 |
| NPDs | COL4A3BP | 0 | 9 | 5.27E-07 | 5.95E-09 |
| NPDs | CREBBP | 3 | 12 | 5.27E-07 | 6.84E-09 |
| NPDs | PTPN11 | 1 | 8 | 5.27E-07 | 7.98E-09 |
| NPDs | PACS1 | 0 | 10 | 5.27E-07 | 1.03E-08 |
| NPDs | EHMT1 | 9 | 2 | 5.27E-07 | 1.32E-08 |
| NPDs | CTCF | 1 | 8 | 5.27E-07 | 1.70E-08 |
| NPDs | TBR1 | 4 | 5 | 5.27E-07 | 2.07E-08 |
| NPDs | TCF20 | 10 | 0 | 5.27E-07 | 2.95E-08 |
| NPDs | CNOT3 | 4 | 5 | 5.27E-07 | 3.88E-08 |
| NPDs | PUF60 | 4 | 4 | 5.27E-07 | 4.79E-08 |
| NPDs | SMARCA2 | 2 | 9 | 5.27E-07 | 5.95E-08 |
| NPDs | HNRNPU | 8 | 0 | 5.27E-07 | 7.29E-08 |
| NPDs | PPP1CB | 1 | 6 | 5.27E-07 | 8.79E-08 |
| NPDs | CLTC | 6 | 4 | 5.27E-07 | 1.03E-07 |
| NPDs | SET | 6 | 0 | 5.27E-07 | 1.20E-07 |
| NPDs | CACNA1A | 2 | 9 | 5.27E-07 | 1.37E-07 |
| NPDs | NSD1 | 8 | 4 | 5.27E-07 | 1.56E-07 |
| NPDs | CACNA1E | 3 | 10 | 5.27E-07 | 1.75E-07 |
| NPDs | BRAF | 0 | 8 | 5.27E-07 | 1.95E-07 |
| NPDs | GNAI1 | 1 | 6 | 5.27E-07 | 2.18E-07 |
| NPDs | BCL11A | 6 | 3 | 5.27E-07 | 2.43E-07 |
| NPDs | CHD3 | 2 | 10 | 5.27E-07 | 2.70E-07 |
| NPDs | KANSL1 | 8 | 0 | 5.27E-07 | 3.09E-07 |
| NPDs | SMARCA4 | 0 | 12 | 5.27E-07 | 3.52E-07 |
| NPDs | GABRB3 | 2 | 5 | 5.27E-07 | 4.36E-07 |
| NPDs | NFIX | 4 | 3 | 5.27E-07 | 5.22E-07 |
| NPDs | KIAA2022 | 7 | 1 | 5.27E-07 | 6.34E-07 |
| NPDs | SOX5 | 5 | 2 | 5.27E-07 | 7.53E-07 |
| NPDs | CUL3 | 5 | 2 | 5.27E-07 | 8.77E-07 |
| NPDs | KMT5B | 6 | 1 | 5.27E-07 | 1.04E-06 |
| NPDs | FBXO11 | 4 | 3 | 5.27E-07 | 1.22E-06 |
| NPDs | EFTUD2 | 3 | 5 | 5.27E-07 | 1.42E-06 |
| NPDs | SMC3 | 3 | 5 | 5.27E-07 | 1.63E-06 |
| NPDs | PHIP | 6 | 3 | 5.27E-07 | 1.95E-06 |
| NPDs | KCNH1 | 0 | 8 | 5.27E-07 | 2.34E-06 |
| NPDs | CHAMP1 | 6 | 0 | 5.27E-07 | 2.80E-06 |
| NPDs | NAA15 | 6 | 0 | 5.27E-07 | 3.35E-06 |
| NPDs | PBX1 | 1 | 5 | 5.27E-07 | 3.95E-06 |
| NPDs | USP9X | 7 | 3 | 5.27E-07 | 4.89E-06 |
| NPDs | MYT1L | 4 | 4 | 5.27E-07 | 5.92E-06 |
| NPDs | CHD4 | 1 | 9 | 5.27E-07 | 6.96E-06 |
| NPDs | ITPR1 | 0 | 12 | 5.27E-07 | 8.00E-06 |
| NPDs | TCF7L2 | 4 | 2 | 5.27E-07 | 9.17E-06 |
| NPDs | NR2F1 | 3 | 3 | 5.27E-07 | 1.05E-05 |
| NPDs | UPF3B | 5 | 0 | 5.27E-07 | 1.22E-05 |
| NPDs | PIK3CA | 0 | 7 | 5.27E-07 | 1.39E-05 |
| NPDs | HECW2 | 1 | 8 | 5.27E-07 | 1.57E-05 |
| NPDs | UNC80 | 1 | 4 | 5.27E-07 | 1.75E-05 |
| NPDs | KIF1A | 0 | 10 | 5.27E-07 | 2.02E-05 |
| NPDs | KCNQ3 | 0 | 7 | 5.27E-07 | 2.30E-05 |
| NPDs | ATP1A3 | 0 | 8 | 5.27E-07 | 2.61E-05 |
| NPDs | SLC35A2 | 3 | 2 | 5.27E-07 | 2.96E-05 |
| NPDs | AGO1 | 0 | 7 | 5.27E-07 | 3.31E-05 |
| NPDs | ZC4H2 | 4 | 0 | 5.27E-07 | 3.79E-05 |
| NPDs | NALCN | 2 | 7 | 2.11E-06 | 4.40E-05 |
| NPDs | ZBTB18 | 4 | 1 | 5.27E-06 | 5.33E-05 |
| NPDs | NSD2 | 4 | 1 | 5.27E-06 | 6.26E-05 |
| NPDs | WDR26 | 2 | 3 | 5.27E-06 | 7.24E-05 |
| NPDs | DLG4 | 4 | 2 | 5.27E-06 | 8.24E-05 |
| NPDs | BTF3 | 0 | 4 | 6.32E-06 | 9.43E-05 |
| NPDs | FOXP2 | 4 | 1 | 6.32E-06 | 1.06E-04 |
| NPDs | QRICH1 | 5 | 0 | 6.32E-06 | 1.18E-04 |
| NPDs | HIST1H1E | 4 | 0 | 7.37E-06 | 1.31E-04 |
| NPDs | CAMK2A | 3 | 2 | 7.37E-06 | 1.44E-04 |
| NPDs | SMAD4 | 0 | 5 | 7.37E-06 | 1.60E-04 |
| NPDs | RAB11A | 1 | 3 | 9.48E-06 | 1.79E-04 |
| NPDs | MBD5 | 6 | 0 | 9.48E-06 | 1.99E-04 |
| NPDs | MSL3 | 4 | 0 | 1.16E-05 | 2.21E-04 |
| NPDs | SETBP1 | 5 | 2 | 1.16E-05 | 2.43E-04 |
| NPDs | ZMYND11 | 2 | 3 | 1.16E-05 | 2.65E-04 |
| NPDs | SLC22A23 | 0 | 5 | 1.16E-05 | 2.87E-04 |
| NPDs | KCNB1 | 1 | 5 | 1.16E-05 | 3.08E-04 |
| NPDs | SNAP25 | 1 | 3 | 1.16E-05 | 3.30E-04 |
| NPDs | RAC1 | 0 | 4 | 1.37E-05 | 3.52E-04 |
| NPDs | CSNK1E | 0 | 5 | 1.37E-05 | 3.75E-04 |
| NPDs | SOX11 | 1 | 4 | 1.90E-05 | 4.05E-04 |
| NPDs | NACC1 | 1 | 4 | 1.90E-05 | 4.38E-04 |
| NPDs | CNKSR2 | 5 | 0 | 1.90E-05 | 4.72E-04 |
| NPDs | SYNCRIP | 4 | 0 | 1.90E-05 | 5.08E-04 |
| NPDs | MAP4K4 | 3 | 3 | 1.90E-05 | 5.45E-04 |
| NPDs | PDHA1 | 3 | 1 | 1.90E-05 | 5.90E-04 |
| NPDs | RFX3 | 1 | 4 | 1.90E-05 | 6.35E-04 |
| NPDs | PPP2R1A | 0 | 5 | 1.90E-05 | 6.79E-04 |
| NPDs | LOC400927-CSNK1E | 0 | 5 | 1.90E-05 | 7.26E-04 |
| NPDs | KDM6A | 3 | 3 | 2.00E-05 | 7.83E-04 |
| NPDs | SYT1 | 1 | 3 | 2.53E-05 | 8.48E-04 |
| NPDs | DHDDS | 0 | 4 | 2.63E-05 | 9.14E-04 |
| NPDs | EBF3 | 3 | 2 | 2.84E-05 | 9.83E-04 |
| NPDs | YWHAG | 0 | 4 | 2.84E-05 | 1.05E-03 |
| NPDs | GABBR2 | 0 | 6 | 3.05E-05 | 1.13E-03 |
| NPDs | GFOD2 | 1 | 2 | 3.05E-05 | 1.20E-03 |
| NPDs | CYP27C1 | 1 | 3 | 3.37E-05 | 1.29E-03 |
| NPDs | GABRA1 | 0 | 4 | 3.48E-05 | 1.37E-03 |
| NPDs | POU3F3 | 2 | 2 | 3.48E-05 | 1.45E-03 |
| NPDs | SRCAP | 9 | 1 | 3.79E-05 | 1.54E-03 |
| NPDs | TUBA1A | 1 | 3 | 3.79E-05 | 1.63E-03 |
| NPDs | BRPF1 | 5 | 1 | 4.74E-05 | 1.74E-03 |
| NPDs | GABRG2 | 0 | 4 | 4.74E-05 | 1.85E-03 |
| NPDs | ARHGEF9 | 2 | 2 | 4.74E-05 | 1.96E-03 |
| NPDs | CBL | 1 | 4 | 4.95E-05 | 2.07E-03 |
| NPDs | CPSF7 | 1 | 3 | 6.32E-05 | 2.21E-03 |
| NPDs | CSNK2B | 2 | 1 | 6.42E-05 | 2.35E-03 |
| NPDs | SPAST | 2 | 2 | 6.74E-05 | 2.50E-03 |
| NPDs | TNPO3 | 2 | 3 | 7.06E-05 | 2.64E-03 |
| NPDs | PRKD1 | 0 | 5 | 7.69E-05 | 2.81E-03 |
| NPDs | ERI1 | 2 | 1 | 8.85E-05 | 2.99E-03 |
| NPDs | HIST1H4E | 0 | 3 | 9.16E-05 | 3.18E-03 |
| NPDs | GRIN2A | 1 | 6 | 1.02E-04 | 3.41E-03 |
| NPDs | ASB14 | 1 | 2 | 1.08E-04 | 3.64E-03 |
| NPDs | NRXN1 | 2 | 5 | 1.11E-04 | 3.88E-03 |
| NPDs | PHF7 | 2 | 1 | 1.28E-04 | 4.16E-03 |
| NPDs | SF1 | 2 | 2 | 1.37E-04 | 4.45E-03 |
| NPDs | FANCE | 3 | 0 | 1.43E-04 | 4.77E-03 |
| NPDs | TNPO2 | 1 | 4 | 1.44E-04 | 5.08E-03 |
| NPDs | PPP2CA | 1 | 2 | 1.82E-04 | 5.45E-03 |
| NPDs | FGF12 | 0 | 3 | 1.90E-04 | 5.83E-03 |
| NPDs | DYNC1H1 | 2 | 13 | 1.95E-04 | 6.21E-03 |
| NPDs | RNF146 | 1 | 2 | 1.97E-04 | 6.60E-03 |
| NPDs | LAMB1 | 3 | 4 | 2.02E-04 | 6.98E-03 |
| NPDs | MYO1E | 3 | 2 | 2.18E-04 | 7.38E-03 |
| NPDs | CAMK2B | 2 | 2 | 2.30E-04 | 7.79E-03 |
| NPDs | FAM200A | 2 | 0 | 2.35E-04 | 8.19E-03 |
| NPDs | SSBP3 | 2 | 1 | 2.41E-04 | 8.60E-03 |
| NPDs | PRKAR1A | 2 | 1 | 2.54E-04 | 9.02E-03 |
| NPDs | ARHGAP15 | 2 | 1 | 3.20E-04 | 1.04E-02 |
| NPDs | SMAD6 | 2 | 1 | 3.20E-04 | 1.09E-02 |
| NPDs | G3BP1 | 2 | 1 | 3.22E-04 | 1.14E-02 |
| NPDs | TAF13 | 2 | 0 | 3.24E-04 | 1.19E-02 |
| NPDs | DCX | 2 | 1 | 3.35E-04 | 1.24E-02 |
| NPDs | MAP2K1 | 0 | 3 | 3.60E-04 | 1.30E-02 |
| NPDs | VEZF1 | 2 | 1 | 3.68E-04 | 1.36E-02 |
| NPDs | AKT3 | 1 | 2 | 3.68E-04 | 1.41E-02 |
| NPDs | SIN3A | 3 | 2 | 3.68E-04 | 1.47E-02 |
| NPDs | HK1 | 1 | 4 | 3.71E-04 | 1.52E-02 |
| NPDs | SON | 6 | 1 | 3.77E-04 | 1.58E-02 |
| NPDs | NR4A2 | 1 | 3 | 3.98E-04 | 1.64E-02 |
| NPDs | SMARCD1 | 2 | 1 | 4.06E-04 | 1.70E-02 |
| NPDs | DEAF1 | 0 | 4 | 4.06E-04 | 1.76E-02 |
| NPDs | ENO1 | 0 | 3 | 4.09E-04 | 1.82E-02 |
| NPDs | STXBP3 | 2 | 1 | 4.32E-04 | 1.88E-02 |
| NPDs | LZTR1 | 1 | 4 | 4.43E-04 | 1.94E-02 |
| NPDs | HNRNPK | 2 | 1 | 4.85E-04 | 2.00E-02 |
| NPDs | RPL26 | 2 | 0 | 4.92E-04 | 2.07E-02 |
| NPDs | CELF2 | 3 | 0 | 4.92E-04 | 2.13E-02 |
| NPDs | TAB2 | 3 | 0 | 5.40E-04 | 2.20E-02 |
| NPDs | PHF5A | 1 | 1 | 5.40E-04 | 2.27E-02 |
| NPDs | AGO3 | 2 | 2 | 5.51E-04 | 2.34E-02 |
| NPDs | EYA1 | 3 | 0 | 5.51E-04 | 2.41E-02 |
| NPDs | PDK2 | 0 | 3 | 5.74E-04 | 2.48E-02 |
| NPDs | C9orf142 | 2 | 0 | 5.75E-04 | 2.55E-02 |
| NPDs | TFAP2C | 2 | 1 | 6.15E-04 | 2.63E-02 |
| NPDs | GRIK1 | 2 | 2 | 6.16E-04 | 2.70E-02 |
| NPDs | PRR14L | 2 | 0 | 6.20E-04 | 2.77E-02 |
| NPDs | TCTE3 | 2 | 0 | 6.26E-04 | 2.85E-02 |
| NPDs | NUDT4 | 1 | 1 | 6.27E-04 | 2.92E-02 |
| NPDs | GLRA2 | 0 | 3 | 6.38E-04 | 2.99E-02 |
| NPDs | BRD7 | 3 | 0 | 6.53E-04 | 3.07E-02 |
| NPDs | ARIH1 | 2 | 1 | 6.57E-04 | 3.14E-02 |
| NPDs | GNAS | 0 | 5 | 6.58E-04 | 3.22E-02 |
| NPDs | RBM12 | 1 | 3 | 6.62E-04 | 3.29E-02 |
| NPDs | GNB2 | 1 | 2 | 6.90E-04 | 3.37E-02 |
| NPDs | SMARCC2 | 3 | 2 | 7.09E-04 | 3.44E-02 |
| NPDs | FXYD5 | 2 | 0 | 7.16E-04 | 3.52E-02 |
| NPDs | MEIS2 | 2 | 1 | 7.23E-04 | 3.60E-02 |
| NPDs | BIRC5 | 2 | 0 | 7.28E-04 | 3.67E-02 |
| NPDs | KIF5C | 0 | 4 | 7.38E-04 | 3.75E-02 |
| NPDs | RPL4 | 0 | 3 | 7.75E-04 | 3.83E-02 |
| NPDs | ASXL1 | 5 | 0 | 7.94E-04 | 3.91E-02 |
| NPDs | TIFA | 1 | 1 | 7.95E-04 | 3.99E-02 |
| NPDs | DPF2 | 0 | 3 | 8.07E-04 | 4.07E-02 |
| NPDs | H2AFV | 0 | 2 | 8.10E-04 | 4.14E-02 |
| NPDs | PSD3 | 3 | 1 | 8.13E-04 | 4.22E-02 |
| NPDs | MARK2 | 2 | 2 | 8.18E-04 | 4.30E-02 |
| NPDs | ABI2 | 0 | 3 | 8.19E-04 | 4.38E-02 |
| NPDs | PHF21A | 3 | 0 | 8.39E-04 | 4.46E-02 |
| NPDs | C1orf123 | 1 | 1 | 8.60E-04 | 4.54E-02 |
| NPDs | VAMP2 | 0 | 2 | 9.27E-04 | 4.62E-02 |
| NPDs | LMTK3 | 3 | 1 | 9.30E-04 | 4.70E-02 |
| NPDs | TFAP4 | 0 | 3 | 9.36E-04 | 4.79E-02 |
| NPDs | UGT1A3 | 1 | 2 | 9.66E-04 | 4.87E-02 |
| NPDs | SPRED2 | 3 | 0 | 9.67E-04 | 4.95E-02 |
| NPDs | LMO2 | 1 | 1 | 9.87E-04 | 5.12E-02 |
| NPDs | CFAP45 | 2 | 1 | 1.12E-03 | 5.63E-02 |
| NPDs | PRKAR1B | 1 | 2 | 1.13E-03 | 5.72E-02 |
| NPDs | AUTS2 | 5 | 0 | 1.13E-03 | 5.81E-02 |
| NPDs | PLAC8L1 | 0 | 2 | 1.17E-03 | 5.99E-02 |
| NPDs | TAOK1 | 1 | 3 | 1.37E-03 | 6.73E-02 |
| NPDs | TCF12 | 2 | 1 | 1.39E-03 | 7.11E-02 |
| NPDs | HIST1H4C | 0 | 2 | 1.40E-03 | 7.30E-02 |
| NPDs | MECOM | 1 | 3 | 1.43E-03 | 7.67E-02 |
| NPDs | ATP1B1 | 2 | 0 | 1.61E-03 | 8.35E-02 |
| NPDs | NTRK2 | 0 | 3 | 1.62E-03 | 8.44E-02 |
| NPDs | SNX11 | 1 | 1 | 1.62E-03 | 8.54E-02 |
| NPDs | GRIN1 | 1 | 4 | 1.64E-03 | 8.64E-02 |
| NPDs | NUDT17 | 2 | 0 | 1.66E-03 | 8.83E-02 |
| NPDs | PDX1 | 1 | 1 | 1.79E-03 | 9.23E-02 |
| NPDs | PRKG1 | 1 | 2 | 1.90E-03 | 9.44E-02 |
| NPDs | SIX3 | 0 | 3 | 2.04E-03 | 9.97E-02 |
| NPDs | HIST1H2AC | 0 | 2 | 2.09E-03 | 1.03E-01 |
| NPDs | PLEKHB2 | 1 | 1 | 2.16E-03 | 1.06E-01 |
| NPDs | FAM104A | 0 | 2 | 2.18E-03 | 1.07E-01 |
| NPDs | ILF2 | 2 | 0 | 2.23E-03 | 1.08E-01 |
| NPDs | SOX4 | 0 | 3 | 2.30E-03 | 1.10E-01 |
| NPDs | MPPED2 | 0 | 2 | 2.58E-03 | 1.18E-01 |
| NPDs | STK33 | 2 | 0 | 2.73E-03 | 1.23E-01 |
| NPDs | FIGN | 2 | 1 | 3.04E-03 | 1.33E-01 |
| NPDs | RAD51 | 0 | 2 | 3.05E-03 | 1.34E-01 |
| NPDs | GOLPH3 | 1 | 1 | 3.07E-03 | 1.35E-01 |
| NPDs | PAPOLG | 0 | 3 | 3.37E-03 | 1.41E-01 |
| NPDs | GALNT18 | 2 | 1 | 3.42E-03 | 1.42E-01 |
| NPDs | SLC12A2 | 1 | 3 | 3.42E-03 | 1.43E-01 |
| NPDs | PI4K2B | 0 | 2 | 3.43E-03 | 1.44E-01 |
| NPDs | PRSS48 | 0 | 2 | 3.43E-03 | 1.46E-01 |
| NPDs | SIAH1 | 0 | 2 | 3.43E-03 | 1.47E-01 |
| NPDs | FOSL2 | 2 | 0 | 3.82E-03 | 1.63E-01 |
| NPDs | EIF4A2 | 1 | 1 | 3.98E-03 | 1.66E-01 |
| NPDs | TANC2 | 2 | 4 | 3.98E-03 | 1.69E-01 |
| NPDs | LARP7 | 2 | 0 | 4.33E-03 | 1.79E-01 |
| NPDs | RAB11B | 0 | 2 | 4.44E-03 | 1.84E-01 |
| NPDs | USP7 | 1 | 3 | 5.09E-03 | 2.01E-01 |
| NPDs | MORC2 | 0 | 4 | 5.14E-03 | 2.03E-01 |
| NPDs | ANK2 | 6 | 3 | 5.27E-03 | 2.09E-01 |
| NPDs | MSI1 | 1 | 1 | 5.32E-03 | 2.12E-01 |
| NPDs | COL23A1 | 2 | 0 | 5.34E-03 | 2.15E-01 |
| NPDs | DCAF7 | 0 | 2 | 5.43E-03 | 2.19E-01 |
| NPDs | SMPD2 | 1 | 1 | 5.55E-03 | 2.21E-01 |
| NPDs | PISD | 2 | 0 | 6.18E-03 | 2.33E-01 |
| NPDs | ZBTB10 | 2 | 0 | 6.35E-03 | 2.40E-01 |
| NPDs | NONO | 1 | 1 | 6.43E-03 | 2.47E-01 |
| NPDs | TMEM26 | 0 | 2 | 6.47E-03 | 2.49E-01 |
| NPDs | RPUSD1 | 1 | 1 | 6.63E-03 | 2.55E-01 |
| NPDs | GRIA2 | 1 | 2 | 7.33E-03 | 2.74E-01 |
| NPDs | PNKD | 0 | 2 | 7.35E-03 | 2.75E-01 |
| NPDs | TGFB2 | 1 | 1 | 7.62E-03 | 2.84E-01 |
| NPDs | NFE2L2 | 1 | 1 | 7.75E-03 | 2.86E-01 |
| NPDs | IL1RAPL2 | 2 | 0 | 7.91E-03 | 2.92E-01 |
| NPDs | CLDN5 | 0 | 2 | 8.05E-03 | 2.95E-01 |
| NPDs | FAM84A | 1 | 1 | 8.11E-03 | 2.97E-01 |
| NPDs | SGCE | 0 | 2 | 8.25E-03 | 3.01E-01 |
| NPDs | ACHE | 1 | 2 | 8.51E-03 | 3.08E-01 |
| NPDs | ACTC1 | 0 | 2 | 8.62E-03 | 3.11E-01 |
| NPDs | GABRP | 0 | 2 | 8.62E-03 | 3.12E-01 |
| NPDs | KCNA2 | 0 | 2 | 9.35E-03 | 3.30E-01 |
| NPDs | GNB1 | 0 | 2 | 9.65E-03 | 3.43E-01 |
| NPDs | NR6A1 | 0 | 2 | 9.78E-03 | 3.50E-01 |
| NPDs | SNRPB2 | 1 | 0 | 1.04E-02 | 3.62E-01 |
| NPDs | LRRC3C | 1 | 0 | 1.10E-02 | 3.72E-01 |
| NPDs | KCNQ5 | 1 | 2 | 1.13E-02 | 3.79E-01 |
| NPDs | ASH1L | 6 | 0 | 1.46E-02 | 4.44E-01 |
| NPDs | KCNT1 | 1 | 4 | 1.73E-02 | 4.82E-01 |
| NPDs | ANO3 | 0 | 3 | 1.79E-02 | 4.93E-01 |
| NPDs | PRPF8 | 2 | 5 | 1.88E-02 | 5.05E-01 |
| NPDs | EBF2 | 1 | 1 | 2.32E-02 | 5.48E-01 |
| NPDs | CLCN4 | 0 | 3 | 2.40E-02 | 5.53E-01 |
| NPDs | DSCAM | 4 | 2 | 2.69E-02 | 5.83E-01 |
| NPDs | TAF1 | 0 | 5 | 3.78E-02 | 6.45E-01 |
| NPDs | ERBB4 | 2 | 1 | 5.50E-02 | 7.09E-01 |
| NPDs | GIGYF1 | 3 | 0 | 5.57E-02 | 7.12E-01 |
| NPDs | AGO2 | 0 | 3 | 1.08E-01 | 7.89E-01 |
| NPDs | COL4A1 | 0 | 4 | 2.01E-01 | 8.46E-01 |
| NPDs | SMPD4 | 0 | 2 | 2.70E-01 | 8.69E-01 |
| NPDs | ATP8A1 | 0 | 2 | 3.15E-01 | 8.81E-01 |
| NPDs | WDFY3 | 3 | 3 | 5.90E-01 | 9.22E-01 |
| NPDs | DOCK1 | 0 | 3 | 7.55E-01 | 9.35E-01 |

Abbreviation: ASD, autism spectrum disorder; UDD, undiagnosed developmental disorder; EE, epileptic encephalopathy; ID, intellectual disability; SCZ, schizophrenia; NPDs, integration of five disorders; LoF, loss-of-function variants; Dmis, deleterious missense variants; Pfun, putative functional variants; P-value and FDR were calculated by transmitted and de novo association (TADA) package.

**Table S4. Contribution of candidate genes to each disorder.**

| **Disorder** | **NO. of NPDs patients** | **NO. of candidate genes** | **NO. of Pfun DNMs** | **NO. of patients** | **Percent of patients (%)** |
| --- | --- | --- | --- | --- | --- |
| ASD | 6,511 | 202 | 478 | 465 | 7.14 |
| UDD | 4,293 | 273 | 1,223 | 1,175 | 27.37 |
| EE | 933 | 95 | 208 | 201 | 21.54 |
| ID | 1,022 | 127 | 279 | 274 | 26.81 |
| SCZ | 1,094 | 35 | 38 | 35 | 3.20 |
| NPDs | 13,858 | 321 | 2,226 | 2,150 | 15.51 |

Abbreviation: ASD, autism spectrum disorder; UDD, undiagnosed developmental disorder; EE, epileptic encephalopathy; ID, intellectual disability; SCZ, schizophrenia; NPDs, integration of five disorders; Pfun, putative functional variants; NO. of NPDs patients, number of patients in each condition; NO. of candidate genes, number of genes in each condition carry Pfun variants; NO. of Pfun DNMs, number of Pfun variants in each condition; NO. of patients, number of patients in each condition carry Pfun variants; Percent of patients, the proportion of patients of each condition carry Pfun variants.

**Table S5. Disorder-biased genes identified in this study.**

| **Disorder** | **Gene** | **N_NPDs** | **N_Disorder** | **P** | **Padj** | **OR** | **95% CI** | |
| --- | --- | --- | --- | --- | --- | --- | --- | --- |
| EE | SCN1A | 29 | 23 | 3.59E-22 | 3.41E-20 | 53.08 | 21.01 | 159.38 |
| EE | IRF2BPL | 14 | 10 | 1.49E-09 | 7.09E-08 | 34.62 | 9.99 | 151.22 |
| EE | SCN2A | 48 | 9 | 4.32E-03 | 3.38E-02 | 3.20 | 1.36 | 6.71 |
| EE | SCN8A | 19 | 7 | 1.53E-04 | 2.08E-03 | 8.08 | 2.69 | 22.25 |
| EE | DNM1 | 12 | 6 | 6.04E-05 | 1.44E-03 | 13.85 | 3.70 | 51.80 |
| EE | CDKL5 | 12 | 6 | 6.04E-05 | 1.44E-03 | 13.85 | 3.70 | 51.80 |
| EE | GABRB2 | 9 | 5 | 1.39E-04 | 2.08E-03 | 17.31 | 3.73 | 87.23 |
| EE | CACNA1A | 11 | 4 | 4.62E-03 | 3.38E-02 | 7.91 | 1.70 | 31.13 |
| EE | CLTC | 10 | 4 | 3.11E-03 | 3.38E-02 | 9.23 | 1.92 | 38.93 |
| EE | KCNT1 | 5 | 4 | 9.73E-05 | 1.85E-03 | 55.39 | 5.48 | 2727.88 |
| EE | GABBR2 | 6 | 3 | 5.23E-03 | 3.55E-02 | 13.85 | 1.85 | 103.39 |
| ID | POGZ | 20 | 8 | 4.92E-05 | 6.25E-03 | 8.37 | 2.97 | 22.27 |
| ID | KAT6B | 14 | 5 | 2.48E-03 | 4.38E-02 | 6.97 | 1.84 | 23.18 |
| ID | DLG4 | 6 | 4 | 3.94E-04 | 1.70E-02 | 25.11 | 3.60 | 277.58 |
| ID | TBR1 | 9 | 4 | 2.76E-03 | 4.38E-02 | 10.04 | 1.99 | 46.66 |
| ID | PHIP | 9 | 4 | 2.76E-03 | 4.38E-02 | 10.04 | 1.99 | 46.66 |
| ID | GRIN2A | 7 | 4 | 8.64E-04 | 2.74E-02 | 16.74 | 2.83 | 114.28 |
| ID | USP7 | 4 | 3 | 1.52E-03 | 3.85E-02 | 37.66 | 3.02 | 1977.28 |
| UDD | ADNP | 27 | 20 | 5.17E-06 | 2.38E-04 | 6.36 | 2.58 | 17.81 |
| UDD | ANKRD11 | 40 | 32 | 2.32E-10 | 6.40E-08 | 8.91 | 4.02 | 22.37 |
| UDD | ARID1B | 48 | 32 | 4.15E-07 | 3.82E-05 | 4.45 | 2.37 | 8.69 |
| UDD | ASXL3 | 22 | 14 | 1.89E-03 | 2.31E-02 | 3.90 | 1.53 | 10.72 |
| UDD | DDX3X | 37 | 25 | 6.26E-06 | 2.47E-04 | 4.64 | 2.25 | 10.14 |
| UDD | DYRK1A | 25 | 18 | 2.96E-05 | 8.18E-04 | 5.73 | 2.28 | 16.22 |
| UDD | EP300 | 19 | 16 | 2.50E-06 | 1.38E-04 | 11.88 | 3.40 | 63.61 |
| UDD | KCNQ2 | 28 | 16 | 6.44E-03 | 4.68E-02 | 2.97 | 1.32 | 6.88 |
| UDD | KMT2A | 36 | 28 | 1.01E-08 | 1.39E-06 | 7.79 | 3.46 | 19.79 |
| UDD | MECP2 | 18 | 14 | 5.91E-05 | 1.36E-03 | 7.79 | 2.45 | 32.52 |
| UDD | MED13L | 26 | 19 | 1.25E-05 | 3.97E-04 | 6.04 | 2.43 | 17.01 |
| UDD | SATB2 | 15 | 13 | 1.30E-05 | 3.97E-04 | 14.47 | 3.28 | 132.12 |
| UDD | SETD5 | 20 | 17 | 9.06E-07 | 6.25E-05 | 12.62 | 3.65 | 67.21 |
| UDD | TCF4 | 18 | 12 | 3.22E-03 | 2.96E-02 | 4.45 | 1.55 | 14.46 |
| UDD | FOXP1 | 18 | 12 | 3.22E-03 | 2.96E-02 | 4.45 | 1.55 | 14.46 |
| UDD | CTNNB1 | 17 | 11 | 6.12E-03 | 4.57E-02 | 4.08 | 1.38 | 13.44 |
| UDD | CDK13 | 14 | 12 | 3.64E-05 | 9.14E-04 | 13.36 | 2.97 | 122.92 |
| UDD | PURA | 14 | 10 | 2.19E-03 | 2.33E-02 | 5.57 | 1.61 | 24.32 |
| UDD | TBL1XR1 | 13 | 10 | 8.69E-04 | 1.20E-02 | 7.42 | 1.91 | 41.97 |
| UDD | SMC1A | 10 | 9 | 1.90E-04 | 3.28E-03 | 20.04 | 2.78 | 878.46 |
| UDD | CASK | 12 | 9 | 2.18E-03 | 2.33E-02 | 6.68 | 1.67 | 38.36 |
| UDD | KAT6A | 13 | 10 | 8.69E-04 | 1.20E-02 | 7.42 | 1.91 | 41.97 |
| UDD | SMARCA2 | 11 | 10 | 6.45E-05 | 1.37E-03 | 22.27 | 3.17 | 966.42 |
| UDD | MEF2C | 11 | 8 | 5.37E-03 | 4.12E-02 | 5.94 | 1.43 | 34.75 |
| UDD | NSD1 | 12 | 10 | 2.79E-04 | 4.52E-03 | 11.13 | 2.37 | 104.51 |
| UDD | EHMT1 | 11 | 9 | 7.55E-04 | 1.16E-02 | 10.02 | 2.07 | 95.31 |
| UDD | PACS1 | 10 | 8 | 2.01E-03 | 2.31E-02 | 8.91 | 1.78 | 86.10 |
| UDD | HNRNPU | 8 | 7 | 1.60E-03 | 2.10E-02 | 15.59 | 2.00 | 702.54 |
| UDD | CTCF | 9 | 7 | 5.26E-03 | 4.12E-02 | 7.79 | 1.48 | 76.89 |
| UDD | CNOT3 | 9 | 7 | 5.26E-03 | 4.12E-02 | 7.79 | 1.48 | 76.89 |
| UDD | NFIX | 7 | 6 | 4.55E-03 | 3.93E-02 | 13.36 | 1.62 | 614.59 |
| UDD | KIF1A | 10 | 8 | 2.01E-03 | 2.31E-02 | 8.91 | 1.78 | 86.10 |
| UDD | HECW2 | 9 | 7 | 5.26E-03 | 4.12E-02 | 7.79 | 1.48 | 76.89 |

N_NPDs, number of putative functional variants in all NPDs; N_Disorder, number of putative functional variants in specific disorder; OR, odds ratio; CI, confidence interval. We performed Poisson rate test to calculate the bias level of gene. The Benjamini and Hochberg false discovery rate procedure was used to adjust for multiple testing.

**Table S6. Novel genes related to human brain disorders.**

| **Gene** | **Disorders** | **Pubmed ID** |
| --- | --- | --- |
| ARHGEF9 | Epileptic encephalopathy/intellectual disability | 29130122 |
| ATP1A3 | Cerebellar ataxia | 29435658 |
| FGF12 | Early onset epilepsy | 29699863 |
| GABRA1 | Epileptic encephalopathies | 26918889 |
| GNB1 | Neurodevelopmental Disability, Hypotonia, Seizures | 27108799 |
| KCNA2 | Epileptic encephalopathies; hereditary spastic paraplegias and ataxia | 25751627; 29050392; 27543892 |
| KCNT1 | Malignant migrating partial seizures of infancy | 23086397 |
| KIAA2022 | Intellectual disability, intractable epilepsy | 27358180 |
| MORC2 | Axonal Charcot-Marie-Tooth disease | 26659848; 28771897 |
| NACC1 | Infantile Epilepsy, Cataracts, Developmental Delay | 28132692 |
| NALCN | Cognitive delay; psychomotor retardation | 24075186; 29168298; 29968795 |
| SGCE | Myoclonus-dystonia | 23332219; 24297365 |
| SIX3 | Holoprosencephaly | 19353631 |
| SOX11 | Coffin–Siris syndrome | 26543203 |
| TUBA1A | Classical lissencephaly | 18954413 |
| PRKAR1B | Novel late-onset neurodegenerative disorder | 24722252 |
| GFOD2 | Schizophrenia | 32197942; 25056061 |
| SLC22A23 | Attention deficit hyperactivity disorder | 25261991;26941099 |
| SNAP25 | Schizophrenia; attention deficit hyperactivity disorder; Parkinson | 30610939; 26941099; 30334187 |
| CSNK1E | Bipolar; epileptic encephalopathy; schizophrenia | 30445897; 30488659; 27236410 |
| AKT3 | Developmental disorders and extreme megalencephaly | 28969385; 22729224 |
| DHDDS | Developmental and Epileptic Encephalopathies | 29100083 |
| GABRG2 | Epileptic encephalopathies | 27864268; 27367160 |
| PPP2CA | Intellectual disability and developmental delay | 30595372 |
| CSNK2B | Schizophrenia; seizures; intellectual disability | 29483533; 30655572; 28585349 |
| ERI1 | Intellectual Disability | 28488351 |
| BRD7 | Impaired cognitive | 25721744 |
| SOX4 | Neurodevelopmental Disease | 30661772 |
| CAMK2B | Neurodevelopmental disorders; Intellectual Disability | 29560374 |
| FANCE | Fanconi anemia | 16127171 |
| LAMB1 | ASD; cobblestone brain malformation;  Cystic leukoencephalopathy with cortical dysplasia | 25774865; 23472759; 25925986 |
| HIST1H4C | Neurodevelopmental syndrome | 28920961 |
| ARHGAP15 | Cognitive deficits | 27713499 |
| SMAD6 | Neurodevelopment; intellectual disability | 31592950; 27479843 |
| G3BP1 | Amyotrophic lateral sclerosis | 29373831 |
| ANO3 | Craniocervical dystonia with levodopa-responsive parkinsonism; febrile seizures | 30692049; 25344690 |
| DCX | lissencephaly cognitive, language impairment, cerebral palsy, epileptic seizures | 20301364 |
| CLCN4 | Intellectual disability and epilepsy | 27550844 |
| SMPD4 | Microcephaly and Congenital Arthrogryposis | 31495489 |
| RAD51 | Fanconi anemia | 26681308; 26253028 |
| KCNQ5 | Intellectual Disability or Epileptic Encephalopathy | 28669405 |
| PI4K2B | Bipolar disorder | 19539307 |
| SIAH1 | Developmental delay, hypotonia and dysmorphic features | 32430360 |
| LARP7 | Microcephalic; intellectual disability | 30006060; 26607181 |
| GRIN1 | Developmental delay, severe intellectual disability | 27164704; 31176596 |
| EYA1 | Marfan syndrome | 23552953 |
| TANC2 | Neurodevelopmental syndrome | 31616000 |
| PISD | Liberfarb syndrome | 31263216 |
| COL4A1 | Epilepsy | 31980581 |
| GNAS | Pseudohypoparathyroidism Ia; Pseudopseudohypoparathyroidism | 17161328 |
| GRIA2 | Neurodevelopmental disorders | 31300657 |
| RBM12 | Psychosis | 28628109 |
| GNB2 | Global developmental delay, intellectual disability | 31698099 |
| IL1RAPL2 | Autism | 18801879 |
| SMARCC2 | Intellectual Disability and Developmental Delay | 30580808 |
| NFE2L2 | Parkinson's disease | 25496089 |
| PNKD | Tourette Disorder | 28894297 |
| DOCK1 | Schizophrenia; brain development disease | 28645778; 24563715 |
| DPF2 | Coffin-Siris Syndrome | 29429572 |
| MARK2 | Alzheimer; Bipolar Disorder | 23001711; 30930738 |
| GABRP | Schizophrenia | 16172613 |
| SPRED2 | Down syndrome; Neurofibromatosis type 1 | 22777171; 19443465 |

**Table S7. Summary information of expression pattern and functional network.**

| **Gene** | **Spatiotemporal module** | **Neocortical module** | **Shared disorders** | **PPI** | **Coexpression** | **RVIS** | **Aggarwala** |
| --- | --- | --- | --- | --- | --- | --- | --- |
| ANKRD11 | 0 | a | 4 | 0 | 0 | -3.13 | 0.95 |
| KMT2A | 1 | 0 | 4 | 20 | 0 | -4.14 | 2.63 |
| CHD8 | 1 | a | 4 | 21 | 41 | -2.36 | 1.96 |
| ARID1B | 1 | 0 | 4 | 8 | 30 | -2.21 | - |
| MED13L | 1 | a | 3 | 3 | 15 | -1.61 | 2.00 |
| ASXL3 | 1 | a | 3 | 0 | 18 | -1.33 | -0.08 |
| SCN1A | 2 | a | 3 | 17 | 2 | -1.85 | 1.98 |
| SCN2A | 2 | a | 4 | 14 | 6 | -2.51 | 2.13 |
| CHD2 | 1 | 0 | 4 | 12 | 9 | - | - |
| SYNGAP1 | 2 | 0 | 4 | 6 | 0 | -2.29 | 2.15 |
| POGZ | 1 | a | 4 | 4 | 36 | -1.96 | 1.11 |
| SETD5 | 1 | 0 | 3 | 0 | 8 | -1.54 | 1.16 |
| KCNQ2 | 1 | b | 4 | 13 | 0 | -1.25 | - |
| ADNP | 1 | c | 3 | 2 | 59 | -1.54 | 0.60 |
| DYRK1A | 1 | c | 3 | 13 | 17 | -0.84 | 0.75 |
| FOXP1 | 0 | a | 3 | 4 | 0 | -1.25 | -0.18 |
| CTNNB1 | 1 | b | 3 | 44 | 0 | -1.07 | 1.57 |
| SLC6A1 | 2 | b | 3 | 1 | 0 | -0.99 | 1.28 |
| STXBP1 | 2 | a | 4 | 13 | 6 | -0.72 | 1.32 |
| DDX3X | 1 | 0 | 4 | 9 | 5 | - | - |
| TCF4 | 1 | 0 | 3 | 12 | 0 | -0.79 | 1.07 |
| PPP2R5D | 0 | a | 3 | 15 | 0 | -0.92 | 1.22 |
| MECP2 | 1 | a | 4 | 15 | 2 | -0.32 | - |
| EP300 | 1 | 0 | 3 | 49 | 35 | -4.11 | 1.34 |
| SATB2 | 1 | a | 2 | 4 | 0 | -1.31 | 1.41 |
| SCN8A | 2 | 0 | 4 | 9 | 8 | -3.09 | 2.10 |
| PURA | 2 | b | 3 | 0 | 0 | -0.43 | 1.17 |
| WDR45 | 2 | a | 3 | 0 | 0 | -0.32 | - |
| GRIN2B | 0 | a | 4 | 0 | 0 | -2.48 | 1.79 |
| GNAO1 | 2 | a | 4 | 16 | 0 | -0.94 | - |
| CDK13 | 1 | 0 | 3 | 6 | 26 | -0.79 | 1.75 |
| TBL1XR1 | 1 | c | 3 | 14 | 0 | -0.43 | 1.02 |
| IRF2BPL | 1 | 0 | 3 | 0 | 22 | -0.98 | 1.49 |
| CSNK2A1 | 1 | a | 4 | 24 | 37 | -0.40 | 0.92 |
| PTEN | 1 | c | 3 | 29 | 0 | -0.16 | 1.08 |
| GATAD2B | 1 | a | 2 | 13 | 31 | -0.65 | 0.88 |
| WAC | 0 | c | 4 | 1 | 0 | -0.48 | 0.66 |
| FOXG1 | 1 | c | 4 | 6 | 19 | -0.29 | 1.47 |
| KAT6B | 1 | c | 2 | 14 | 40 | -3.45 | 1.16 |
| MEF2C | 0 | a | 2 | 14 | 0 | -0.58 | 1.29 |
| SMC1A | 1 | a | 2 | 16 | 35 | -1.95 | - |
| CASK | 1 | a | 4 | 8 | 10 | -1.52 | - |
| IQSEC2 | 2 | a | 4 | 1 | 7 | -1.59 | - |
| CDKL5 | 2 | a | 2 | 7 | 2 | -1.93 | - |
| AHDC1 | 1 | a | 4 | 0 | 3 | -2.17 | 1.31 |
| HDAC8 | 0 | b | 1 | 33 | 0 | -0.45 | - |
| KAT6A | 1 | 0 | 3 | 25 | 53 | -1.76 | 1.58 |
| DNM1 | 2 | a | 3 | 14 | 7 | -0.85 | - |
| SMARCA2 | 0 | a | 2 | 49 | 0 | -2.38 | 1.53 |
| KANSL1 | 1 | b | 1 | 6 | 42 | 0.46 | -0.26 |
| EEF1A2 | 2 | c | 4 | 6 | 0 | -0.72 | 1.38 |
| KDM5B | 1 | 0 | 2 | 12 | 57 | -2.30 | 1.65 |
| NSD1 | 1 | a | 2 | 12 | 39 | -2.68 | 0.91 |
| EHMT1 | 1 | a | 2 | 41 | 32 | -2.09 | 1.54 |
| TLK2 | 1 | 0 | 4 | 1 | 34 | -0.60 | 1.38 |
| DNMT3A | 1 | 0 | 4 | 20 | 22 | -1.96 | 1.48 |
| PACS1 | 1 | a | 3 | 1 | 0 | -1.48 | - |
| SHANK3 | 2 | a | 3 | 7 | 0 | - | - |
| HNRNPU | 1 | c | 2 | 14 | 27 | -0.73 | 1.54 |
| GABRB2 | 2 | a | 4 | 10 | 12 | -1.01 | 1.32 |
| TRIP12 | 1 | c | 3 | 12 | 3 | -3.21 | 2.38 |
| PPM1D | 1 | b | 3 | 1 | 13 | 0.10 | 1.13 |
| CREBBP | 1 | 0 | 4 | 44 | 36 | -4.23 | 2.00 |
| NAA10 | 0 | 0 | 3 | 2 | 0 | -0.50 | - |
| COL4A3BP | 2 | c | 4 | 2 | 10 | -0.66 | 0.46 |
| PTPN11 | 2 | 0 | 2 | 16 | 0 | -0.63 | 1.39 |
| CTCF | 1 | 0 | 2 | 21 | 61 | -1.48 | 1.58 |
| CNOT3 | 1 | 0 | 2 | 2 | 3 | 0.00 | 0.70 |
| TBR1 | 1 | a | 2 | 10 | 8 | -0.84 | - |
| TCF20 | 1 | a | 3 | 2 | 39 | -3.82 | 0.52 |
| NFIX | 1 | a | 2 | 1 | 0 | -0.76 | 1.39 |
| PUF60 | 1 | 0 | 4 | 11 | 0 | -0.88 | 1.40 |
| PPP1CB | 0 | c | 2 | 15 | 0 | -0.14 | 0.76 |
| CLTC | 2 | a | 3 | 13 | 4 | -2.06 | 2.15 |
| SET | 1 | c | 3 | 6 | 22 | -0.03 | 0.60 |
| BRAF | 1 | 0 | 3 | 14 | 1 | -0.97 | 1.57 |
| CACNA1A | 2 | 0 | 3 | 13 | 0 | -2.12 | - |
| CACNA1E | 1 | a | 4 | 1 | 3 | -1.60 | 1.86 |
| KIF1A | 2 | a | 2 | 3 | 0 | -2.95 | 1.99 |
| GNAI1 | 1 | a | 2 | 17 | 0 | -0.56 | - |
| BCL11A | 1 | a | 3 | 8 | 15 | -1.67 | 1.79 |
| CHD3 | 1 | a | 3 | 23 | 13 | -2.36 | 2.66 |
| EFTUD2 | 1 | a | 3 | 21 | 14 | -1.84 | 1.78 |
| SMARCA4 | 1 | a | 3 | 57 | 44 | -3.62 | 2.16 |
| GABRB3 | 2 | a | 4 | 9 | 1 | -0.56 | 0.76 |
| KIAA2022 | 1 | a | 4 | 0 | 19 | -2.04 | - |
| SOX5 | 1 | a | 3 | 0 | 5 | -1.83 | 0.84 |
| CUL3 | 1 | a | 4 | 13 | 8 | -0.98 | 1.41 |
| HECW2 | 2 | 0 | 3 | 6 | 1 | -1.39 | 1.04 |
| KMT5B | 1 | a | 3 | 4 | 0 | - | - |
| CHAMP1 | 1 | b | 2 | 1 | 53 | -1.09 | 0.28 |
| FBXO11 | 1 | c | 3 | 0 | 14 | -1.47 | 1.44 |
| ZBTB18 | 1 | a | 1 | 4 | 0 | -1.23 | 0.73 |
| SMC3 | 1 | b | 4 | 15 | 34 | -1.54 | 1.87 |
| PHIP | 1 | b | 4 | 2 | 6 | -2.79 | 1.34 |
| CNKSR2 | 2 | a | 1 | 4 | 8 | - | - |
| KCNH1 | 2 | 0 | 2 | 6 | 5 | -1.87 | 1.14 |
| USP9X | 1 | a | 3 | 12 | 6 | -3.76 | - |
| DLG4 | 2 | a | 3 | 21 | 0 | -1.24 | 1.39 |
| CHD4 | 1 | 0 | 4 | 24 | 25 | -3.32 | 1.70 |
| NAA15 | 1 | c | 2 | 2 | 32 | -0.95 | 1.37 |
| PBX1 | 1 | a | 3 | 11 | 17 | -0.52 | 0.77 |
| ITPR1 | 2 | a | 4 | 13 | 0 | -4.63 | 2.47 |
| BTF3 | 1 | 0 | 1 | 3 | 9 | -0.03 | 0.83 |
| MYT1L | 1 | a | 3 | 2 | 2 | -1.83 | 1.68 |
| MSL3 | 1 | a | 1 | 6 | 0 | -0.81 | - |
| UPF3B | 1 | 0 | 2 | 14 | 2 | -0.33 | - |
| TCF7L2 | 0 | b | 3 | 12 | 1 | -0.89 | - |
| NR2F1 | 1 | 0 | 3 | 2 | 0 | -0.52 | - |
| PDHA1 | 2 | 0 | 1 | 3 | 0 | -0.44 | - |
| PIK3CA | 1 | c | 2 | 35 | 12 | -1.01 | 1.35 |
| MAP4K4 | 1 | a | 2 | 2 | 0 | -0.73 | 1.80 |
| UNC80 | 2 | a | 2 | 1 | 4 | - | 1.94 |
| KCNT1 | 2 | 0 | 2 | 0 | 1 | -1.73 | - |
| KCNQ3 | 2 | 0 | 4 | 11 | 1 | -1.13 | - |
| ATP1A3 | 2 | a | 3 | 8 | 0 | -1.93 | 1.69 |
| SLC35A2 | 2 | a | 3 | 1 | 0 | -0.46 | - |
| AGO1 | 1 | b | 4 | 8 | 0 | -1.69 | 1.62 |
| KDM6A | 1 | c | 2 | 21 | 17 | -1.54 | - |
| ZC4H2 | 1 | 0 | 2 | 1 | 28 | -0.11 | - |
| NALCN | 2 | 0 | 3 | 8 | 11 | -2.76 | 1.72 |
| SMAD4 | 1 | b | 2 | 20 | 35 | -0.45 | 0.50 |
| NSD2 | 1 | a | 3 | 9 | 0 | - | - |
| WDR26 | 1 | c | 2 | 1 | 8 | -0.61 | - |
| SOX11 | 1 | 0 | 2 | 1 | 35 | - | 1.44 |
| ANK2 | 2 | 0 | 3 | 8 | 0 | -2.98 | 1.09 |
| QRICH1 | 1 | a | 2 | 1 | 34 | -1.31 | 1.01 |
| GRIN2A | 2 | 0 | 4 | 10 | 10 | -2.40 | 1.55 |
| FOXP2 | 0 | b | 3 | 1 | 0 | -1.22 | 1.46 |
| EBF3 | 0 | 0 | 2 | 0 | 0 | -1.17 | - |
| HIST1H1E | 0 | b | 3 | 4 | 0 | -0.61 | -0.22 |
| GABBR2 | 2 | a | 3 | 8 | 0 | -1.24 | - |
| PPP2R1A | 1 | a | 2 | 25 | 0 | -0.97 | 0.88 |
| CAMK2A | 2 | a | 2 | 16 | 3 | -0.71 | 1.32 |
| GFOD2 | 1 | a | 1 | 0 | 0 | -0.80 | 0.34 |
| RAB11A | 1 | 0 | 2 | 18 | 0 | -0.17 | 0.85 |
| MBD5 | 1 | a | 3 | 3 | 13 | -1.99 | 1.29 |
| SETBP1 | 1 | c | 3 | 11 | 38 | -0.96 | 0.77 |
| MYO1E | 0 | 0 | 1 | 4 | 0 | -1.69 | 1.48 |
| TAOK1 | 1 | 0 | 1 | 9 | 2 | -1.40 | 1.36 |
| SLC22A23 | 1 | 0 | 3 | 0 | 0 | -0.93 | 1.16 |
| RAC1 | 0 | a | 2 | 25 | 0 | -0.15 | 0.88 |
| ZMYND11 | 1 | a | 3 | 4 | 0 | -0.95 | 1.42 |
| KCNB1 | 2 | a | 3 | 10 | 5 | -0.27 | 1.60 |
| SNAP25 | 2 | a | 2 | 11 | 9 | -0.10 | 0.89 |
| CSNK1E | 1 | a | 3 | 10 | 33 | - | - |
| NACC1 | 2 | 0 | 4 | 0 | 0 | -0.75 | 1.30 |
| PRKAR1A | 2 | c | 1 | 12 | 5 | -0.58 | 1.16 |
| USP7 | 1 | 0 | 2 | 6 | 3 | -2.07 | 1.59 |
| SYNCRIP | 1 | b | 3 | 6 | 32 | -1.14 | 1.35 |
| TNPO2 | 1 | a | 2 | 4 | 0 | -1.19 | 1.57 |
| RFX3 | 1 | c | 3 | 1 | 5 | -1.25 | 1.39 |
| SYT1 | 2 | a | 2 | 13 | 17 | -0.52 | 1.11 |
| AKT3 | 1 | a | 1 | 17 | 10 | -0.58 | 0.95 |
| LOC400927-CSNK1E | 0 | 0 | 3 | 0 | 0 | - | - |
| SLC12A2 | 2 | c | 1 | 2 | 0 | -1.56 | 1.22 |
| HNRNPK | 1 | c | 1 | 12 | 24 | -0.82 | 1.27 |
| DHDDS | 0 | 0 | 3 | 2 | 1 | 0.01 | 0.35 |
| SIN3A | 1 | b | 2 | 31 | 48 | -2.25 | 1.63 |
| AGO2 | 1 | 0 | 1 | 12 | 0 | -1.71 | 1.87 |
| YWHAG | 2 | a | 3 | 14 | 1 | -0.45 | - |
| TAB2 | 1 | c | 1 | 7 | 0 | -1.12 | 0.89 |
| CYP27C1 | 0 | 0 | 2 | 0 | 0 | -0.89 | 0.68 |
| ARHGEF9 | 2 | a | 2 | 8 | 6 | 0.34 | - |
| HK1 | 2 | a | 2 | 3 | 0 | -1.65 | 1.40 |
| GABRA1 | 2 | a | 3 | 10 | 8 | -0.56 | 0.98 |
| POU3F3 | 1 | 0 | 2 | 3 | 0 | - | 1.56 |
| SRCAP | 1 | 0 | 3 | 16 | 6 | -4.21 | 1.52 |
| TUBA1A | 1 | a | 3 | 14 | 1 | 0.48 | 1.25 |
| MORC2 | 1 | 0 | 1 | 0 | 51 | -1.96 | 1.77 |
| GABRG2 | 2 | a | 3 | 13 | 12 | -0.91 | 0.47 |
| BRPF1 | 1 | a | 3 | 10 | 21 | -2.50 | 2.00 |
| FGF12 | 2 | a | 2 | 2 | 0 | -0.46 | 0.67 |
| WDFY3 | 1 | c | 1 | 2 | 1 | -5.03 | 2.18 |
| CBL | 1 | 0 | 2 | 14 | 11 | -0.78 | 0.93 |
| TCF12 | 1 | b | 1 | 7 | 9 | -0.87 | 0.23 |
| CPSF7 | 1 | 0 | 3 | 8 | 29 | -0.77 | 1.23 |
| PPP2CA | 2 | c | 2 | 28 | 0 | -0.18 | 0.49 |
| CSNK2B | 1 | 0 | 2 | 12 | 0 | -0.02 | 0.89 |
| SPAST | 1 | c | 3 | 3 | 30 | -0.26 | - |
| SF1 | 1 | a | 2 | 11 | 23 | -0.81 | - |
| TNPO3 | 1 | a | 2 | 1 | 46 | -1.79 | 1.31 |
| ASXL1 | 1 | 0 | 2 | 5 | 39 | -0.48 | 0.47 |
| PRKD1 | 0 | b | 2 | 2 | 0 | -0.42 | 1.14 |
| PRKG1 | 0 | b | 1 | 18 | 0 | - | - |
| ERI1 | 1 | b | 2 | 0 | 20 | -0.64 | - |
| HIST1H4E | 0 | b | 2 | 0 | 0 | -0.38 | 0.72 |
| SIX3 | 0 | 0 | 1 | 3 | 0 | 0.04 | 1.24 |
| BRD7 | 1 | b | 1 | 12 | 0 | -0.23 | 0.01 |
| SOX4 | 1 | 0 | 1 | 5 | 18 | -0.13 | 0.92 |
| CAMK2B | 2 | a | 2 | 12 | 0 | -0.56 | - |
| ASB14 | 0 | 0 | 1 | 5 | 0 | - | 0.07 |
| AUTS2 | 1 | a | 2 | 3 | 27 | -1.96 | 1.53 |
| NRXN1 | 2 | a | 3 | 9 | 0 | -1.88 | 1.97 |
| KCNA2 | 2 | 0 | 1 | 9 | 5 | -0.78 | 1.04 |
| PHF7 | 2 | 0 | 2 | 4 | 0 | -0.64 | -0.23 |
| C9orf142 | 0 | 0 | 1 | 1 | 0 | -0.14 | - |
| PHF5A | 1 | c | 1 | 11 | 2 | 0.05 | 0.63 |
| NTRK2 | 0 | a | 2 | 9 | 0 | -1.19 | 0.83 |
| FANCE | 1 | a | 2 | 1 | 32 | 0.70 | - |
| C1orf123 | 0 | 0 | 1 | 0 | 0 | -0.26 | 0.34 |
| LMO2 | 0 | b | 1 | 5 | 0 | -0.08 | - |
| LAMB1 | 0 | 0 | 2 | 3 | 0 | -1.24 | -0.44 |
| VAMP2 | 2 | 0 | 1 | 16 | 1 | 0.23 | 0.66 |
| RNF146 | 1 | c | 2 | 0 | 0 | -0.01 | 0.03 |
| DYNC1H1 | 1 | a | 4 | 10 | 0 | -8.60 | 3.69 |
| PLAC8L1 | 0 | 0 | 1 | 0 | 0 | 0.36 | -0.34 |
| DSCAM | 1 | a | 3 | 2 | 0 | -4.39 | 1.92 |
| FAM200A | 1 | c | 2 | 0 | 16 | - | -0.85 |
| HIST1H4C | 0 | 0 | 1 | 29 | 0 | -0.45 | 0.75 |
| SSBP3 | 1 | a | 2 | 0 | 0 | -0.22 | 1.22 |
| SNX11 | 0 | 0 | 1 | 0 | 0 | -0.27 | 0.00 |
| PRPF8 | 1 | a | 2 | 13 | 35 | -5.03 | 2.71 |
| ARHGAP15 | 0 | a | 3 | 1 | 0 | - | - |
| FIGN | 0 | b | 2 | 0 | 1 | -1.37 | 0.68 |
| SMAD6 | 0 | b | 3 | 5 | 2 | - | - |
| EBF2 | 0 | 0 | 1 | 0 | 0 | -1.07 | 0.85 |
| PDX1 | 0 | c | 1 | 1 | 0 | - | 0.72 |
| G3BP1 | 1 | b | 2 | 5 | 11 | -0.83 | 1.21 |
| TAF13 | 2 | 0 | 1 | 3 | 0 | -0.02 | 0.62 |
| ANO3 | 0 | a | 2 | 0 | 1 | -1.52 | 1.44 |
| PLEKHB2 | 2 | a | 1 | 0 | 8 | 0.06 | 1.14 |
| DCX | 1 | a | 2 | 6 | 24 | -0.67 | - |
| DEAF1 | 0 | a | 3 | 1 | 0 | -0.59 | - |
| HIST1H2AC | 0 | b | 1 | 30 | 0 | -0.48 | 0.42 |
| CLCN4 | 2 | a | 2 | 0 | 0 | -1.56 | - |
| MAP2K1 | 2 | 0 | 2 | 25 | 8 | -0.44 | 1.12 |
| FAM104A | 1 | b | 1 | 0 | 0 | 0.27 | 0.09 |
| SMPD4 | 1 | 0 | 1 | 1 | 0 | -0.15 | 1.30 |
| MPPED2 | 1 | b | 1 | 0 | 0 | -0.50 | 0.99 |
| VEZF1 | 1 | b | 2 | 1 | 31 | -1.01 | 1.30 |
| GOLPH3 | 1 | c | 1 | 2 | 2 | -0.24 | - |
| ASH1L | 2 | 0 | 2 | 32 | 1 | -3.88 | 1.87 |
| SON | 1 | c | 4 | 5 | 17 | -2.98 | 1.13 |
| RAD51 | 1 | b | 1 | 19 | 4 | -0.16 | 0.55 |
| NR4A2 | 1 | a | 3 | 1 | 1 | -0.37 | 0.98 |
| GALNT18 | 2 | a | 1 | 0 | 0 | -1.07 | 0.75 |
| FOSL2 | 0 | 0 | 1 | 2 | 0 | -0.65 | - |
| SMARCD1 | 1 | 0 | 2 | 14 | 42 | -0.61 | - |
| PRSS48 | 0 | 0 | 1 | 0 | 0 | 0.84 | -0.84 |
| ENO1 | 0 | 0 | 2 | 12 | 0 | -0.60 | 0.29 |
| KCNQ5 | 2 | a | 2 | 7 | 3 | - | - |
| PI4K2B | 0 | b | 1 | 1 | 0 | -0.12 | - |
| STXBP3 | 2 | b | 2 | 4 | 0 | - | - |
| PRKAR1B | 2 | 0 | 1 | 12 | 4 | -0.18 | 1.05 |
| SIAH1 | 1 | c | 1 | 6 | 33 | 0.06 | 0.51 |
| LZTR1 | 0 | 0 | 2 | 1 | 0 | -2.27 | 1.27 |
| LARP7 | 1 | b | 1 | 0 | 0 | 0.25 | 0.01 |
| AGO3 | 1 | 0 | 2 | 9 | 0 | -0.93 | 1.71 |
| EIF4A2 | 2 | a | 1 | 14 | 0 | - | - |
| GRIN1 | 2 | a | 4 | 13 | 4 | -1.38 | - |
| RPL26 | 1 | 0 | 2 | 8 | 0 | 0.05 | 0.36 |
| CELF2 | 1 | b | 2 | 1 | 12 | - | - |
| GRIK1 | 0 | 0 | 2 | 1 | 0 | -0.90 | 0.90 |
| COL23A1 | 0 | 0 | 1 | 1 | 0 | 0.48 | - |
| ATP8A1 | 2 | 0 | 1 | 0 | 0 | -1.77 | 1.26 |
| RAB11B | 1 | 0 | 1 | 3 | 0 | -0.34 | 0.97 |
| GIGYF1 | 0 | 0 | 1 | 0 | 0 | -2.62 | 1.03 |
| EYA1 | 0 | 0 | 2 | 3 | 1 | - | - |
| MSI1 | 1 | 0 | 1 | 2 | 10 | -0.36 | - |
| SMPD2 | 0 | 0 | 1 | 1 | 0 | -0.66 | 0.00 |
| TANC2 | 1 | a | 3 | 1 | 5 | -3.88 | 1.33 |
| PDK2 | 2 | 0 | 2 | 5 | 1 | -0.27 | 0.56 |
| TFAP2C | 1 | b | 2 | 5 | 0 | -0.33 | 0.43 |
| PISD | 0 | a | 1 | 0 | 2 | 0.29 | 0.42 |
| ZBTB10 | 1 | c | 1 | 0 | 28 | -0.55 | 1.53 |
| PRR14L | 1 | 0 | 2 | 2 | 15 | - | -1.49 |
| COL4A1 | 0 | b | 2 | 2 | 0 | -2.22 | 0.76 |
| LMTK3 | 0 | a | 2 | 0 | 0 | - | - |
| TCTE3 | 1 | 0 | 2 | 1 | 0 | - | - |
| DCAF7 | 1 | a | 1 | 1 | 54 | - | 0.83 |
| NUDT4 | 2 | c | 2 | 0 | 0 | 0.37 | - |
| GLRA2 | 1 | a | 2 | 7 | 1 | -0.52 | - |
| NONO | 1 | 0 | 1 | 8 | 46 | -0.25 | - |
| GNAS | 0 | a | 2 | 7 | 0 | 0.35 | 0.92 |
| ARIH1 | 1 | a | 2 | 2 | 20 | -0.65 | - |
| RPUSD1 | 0 | 0 | 1 | 0 | 0 | -0.10 | -0.22 |
| GRIA2 | 2 | a | 1 | 10 | 0 | -0.90 | 1.47 |
| RBM12 | 1 | c | 4 | 0 | 37 | -1.51 | 0.65 |
| MEIS2 | 1 | b | 2 | 2 | 0 | -0.52 | 1.11 |
| GNB2 | 1 | a | 3 | 15 | 0 | -0.77 | 0.92 |
| TMEM26 | 2 | 0 | 1 | 0 | 0 | -0.91 | 0.86 |
| FXYD5 | 0 | 0 | 1 | 1 | 2 | 0.30 | -0.88 |
| IL1RAPL2 | 1 | 0 | 1 | 0 | 0 | -0.12 | - |
| TAF1 | 1 | b | 3 | 34 | 11 | -2.47 | - |
| SMARCC2 | 1 | a | 3 | 22 | 12 | -1.46 | 1.26 |
| TGFB2 | 0 | a | 1 | 3 | 0 | -0.58 | 0.79 |
| BIRC5 | 1 | b | 2 | 16 | 3 | 0.62 | - |
| NFE2L2 | 0 | b | 1 | 5 | 1 | 0.12 | -0.30 |
| KIF5C | 2 | a | 3 | 5 | 0 | - | 1.58 |
| ACHE | 0 | a | 1 | 3 | 0 | -0.47 | 0.76 |
| FAM84A | 2 | 0 | 1 | 0 | 0 | 0.16 | 1.14 |
| RPL4 | 1 | a | 3 | 10 | 15 | -0.49 | 0.55 |
| PAPOLG | 1 | c | 1 | 1 | 27 | -1.52 | 0.95 |
| PNKD | 2 | b | 1 | 1 | 0 | 0.80 | 0.33 |
| PSD3 | 2 | a | 3 | 0 | 4 | 0.09 | -1.70 |
| DOCK1 | 0 | b | 2 | 3 | 0 | - | 1.31 |
| TIFA | 1 | b | 2 | 0 | 3 | 0.44 | -0.45 |
| H2AFV | 1 | b | 2 | 32 | 0 | -0.04 | 0.64 |
| ERBB4 | 0 | 0 | 1 | 12 | 0 | -2.65 | 1.37 |
| DPF2 | 1 | b | 2 | 5 | 0 | -0.61 | 1.22 |
| CLDN5 | 0 | 0 | 1 | 0 | 2 | 0.24 | 0.77 |
| ATP1B1 | 2 | 0 | 1 | 4 | 7 | -0.03 | - |
| MARK2 | 2 | a | 3 | 9 | 0 | -1.29 | 1.75 |
| ABI2 | 1 | a | 2 | 3 | 0 | -0.61 | 1.12 |
| SGCE | 0 | b | 1 | 1 | 1 | -0.69 | 0.84 |
| PHF21A | 1 | 0 | 3 | 8 | 22 | -0.53 | 0.73 |
| NUDT17 | 0 | 0 | 1 | 0 | 0 | 0.48 | -0.15 |
| SNRPB2 | 1 | c | 1 | 10 | 7 | -0.23 | 0.81 |
| GABRP | 0 | 0 | 1 | 5 | 0 | 0.24 | -0.15 |
| ACTC1 | 0 | 0 | 1 | 23 | 0 | -0.43 | 1.19 |
| MECOM | 0 | 0 | 2 | 10 | 0 | -0.17 | -0.38 |
| CFAP45 | 0 | b | 2 | 0 | 0 | - | - |
| TFAP4 | 1 | 0 | 2 | 0 | 7 | -0.35 | 0.70 |
| GNB1 | 1 | a | 1 | 16 | 0 | -0.40 | 1.14 |
| ILF2 | 1 | 0 | 1 | 3 | 30 | -0.51 | 1.08 |
| UGT1A3 | 0 | 0 | 3 | 1 | 0 | 0.40 | -1.26 |
| LRRC3C | 0 | 0 | 1 | 3 | 0 | - | -1.13 |
| NR6A1 | 0 | 0 | 1 | 1 | 0 | -0.66 | 0.46 |
| STK33 | 0 | b | 1 | 1 | 0 | -0.05 | 0.10 |
| SPRED2 | 2 | 0 | 3 | 4 | 0 | -0.60 | 0.36 |

Spatiotemporal module and neocortical module were coexpression modules from BrainSpan dataset; Shared disorders, number of disorders carry putative functional DNMs of specific genes; PPI, number of protein-protein interaction for gene with other candidate genes; Coexpression, number of other candidate genes show coexpression with specific genes. RVIS, residual variation intolerance score; Aggarwala, substitution intolerance scores from Aggarwala et al., Nature Genetics 2016.

# **References**

1. Kong A, Frigge ML, Masson G, Besenbacher S, Sulem P, Magnusson G, et al. Rate of de novo mutations and the importance of father's age to disease risk. Nature. 2012;488:471-475.

2. Michaelson JJ, Shi Y, Gujral M, Zheng H, Malhotra D, Jin X, et al. Whole-genome sequencing in autism identifies hot spots for de novo germline mutation. Cell. 2012;151:1431-1442.

3. De Rubeis S, He X, Goldberg AP, Poultney CS, Samocha K, Cicek AE, et al. Synaptic, transcriptional and chromatin genes disrupted in autism. Nature. 2014;515:209-215.

4. Iossifov I, O'Roak BJ, Sanders SJ, Ronemus M, Krumm N, Levy D, et al. The contribution of de novo coding mutations to autism spectrum disorder. Nature. 2014;515:216-221.

5. An JY, Lin K, Zhu L, Werling DM, Dong S, Brand H, et al. Genome-wide de novo risk score implicates promoter variation in autism spectrum disorder. Science. 2018;362.

6. Hashimoto R, Nakazawa T, Tsurusaki Y, Yasuda Y, Nagayasu K, Matsumura K, et al. Whole-exome sequencing and neurite outgrowth analysis in autism spectrum disorder. J Hum Genet. 2016;61:199-206.

7. Li J, Wang L, Guo H, Shi L, Zhang K, Tang M, et al. Targeted sequencing and functional analysis reveal brain-size-related genes and their networks in autism spectrum disorders. Mol Psychiatry. 2017;22:1282-1290.

8. Chen R, Davis LK, Guter S, Wei Q, Jacob S, Potter MH, et al. Leveraging blood serotonin as an endophenotype to identify de novo and rare variants involved in autism. Mol Autism. 2017;8:14.

9. Lim ET, Uddin M, De Rubeis S, Chan Y, Kamumbu AS, Zhang X, et al. Rates, distribution and implications of postzygotic mosaic mutations in autism spectrum disorder. Nat Neurosci. 2017;20:1217-1224.

10. RK CY, Merico D, Bookman M, J LH, Thiruvahindrapuram B, Patel RV, et al. Whole genome sequencing resource identifies 18 new candidate genes for autism spectrum disorder. Nat Neurosci. 2017;20:602-611.

11. Takata A, Miyake N, Tsurusaki Y, Fukai R, Miyatake S, Koshimizu E, et al. Integrative Analyses of De Novo Mutations Provide Deeper Biological Insights into Autism Spectrum Disorder. Cell Rep. 2018;22:734-747.

12. Girard SL, Gauthier J, Noreau A, Xiong L, Zhou S, Jouan L, et al. Increased exonic de novo mutation rate in individuals with schizophrenia. Nat Genet. 2011;43:860-863.

13. Xu B, Ionita-Laza I, Roos JL, Boone B, Woodrick S, Sun Y, et al. De novo gene mutations highlight patterns of genetic and neural complexity in schizophrenia. Nat Genet. 2012;44:1365-1369.

14. Gulsuner S, Walsh T, Watts AC, Lee MK, Thornton AM, Casadei S, et al. Spatial and temporal mapping of de novo mutations in schizophrenia to a fetal prefrontal cortical network. Cell. 2013;154:518-529.

15. Fromer M, Pocklington AJ, Kavanagh DH, Williams HJ, Dwyer S, Gormley P, et al. De novo mutations in schizophrenia implicate synaptic networks. Nature. 2014;506:179-184.

16. McCarthy SE, Gillis J, Kramer M, Lihm J, Yoon S, Berstein Y, et al. De novo mutations in schizophrenia implicate chromatin remodeling and support a genetic overlap with autism and intellectual disability. Mol Psychiatry. 2014;19:652-658.

17. Guipponi M, Santoni FA, Setola V, Gehrig C, Rotharmel M, Cuenca M, et al. Exome sequencing in 53 sporadic cases of schizophrenia identifies 18 putative candidate genes. PLoS One. 2014;9:e112745.

18. Ambalavanan A, Girard SL, Ahn K, Zhou S, Dionne-Laporte A, Spiegelman D, et al. De novo variants in sporadic cases of childhood onset schizophrenia. Eur J Hum Genet. 2016;24:944-948.

19. Vissers LE, de Ligt J, Gilissen C, Janssen I, Steehouwer M, de Vries P, et al. A de novo paradigm for mental retardation. Nat Genet. 2010;42:1109-1112.

20. Rauch A, Wieczorek D, Graf E, Wieland T, Endele S, Schwarzmayr T, et al. Range of genetic mutations associated with severe non-syndromic sporadic intellectual disability: an exome sequencing study. Lancet. 2012;380:1674-1682.

21. de Ligt J, Willemsen MH, van Bon BW, Kleefstra T, Yntema HG, Kroes T, et al. Diagnostic exome sequencing in persons with severe intellectual disability. N Engl J Med. 2012;367:1921-1929.

22. Gilissen C, Hehir-Kwa JY, Thung DT, van de Vorst M, van Bon BW, Willemsen MH, et al. Genome sequencing identifies major causes of severe intellectual disability. Nature. 2014;511:344-347.

23. Hamdan FF, Srour M, Capo-Chichi JM, Daoud H, Nassif C, Patry L, et al. De novo mutations in moderate or severe intellectual disability. PLoS Genet. 2014;10:e1004772.

24. Lelieveld SH, Reijnders MR, Pfundt R, Yntema HG, Kamsteeg EJ, de Vries P, et al. Meta-analysis of 2,104 trios provides support for 10 new genes for intellectual disability. Nat Neurosci. 2016;19:1194-1196.

25. Veeramah KR, Johnstone L, Karafet TM, Wolf D, Sprissler R, Salogiannis J, et al. Exome sequencing reveals new causal mutations in children with epileptic encephalopathies. Epilepsia. 2013;54:1270-1281.

26. Epi KC, Epilepsy Phenome/Genome P, Allen AS, Berkovic SF, Cossette P, Delanty N, et al. De novo mutations in epileptic encephalopathies. Nature. 2013;501:217-221.

27. Euro E-RESC, Epilepsy Phenome/Genome P, Epi KC. De novo mutations in synaptic transmission genes including DNM1 cause epileptic encephalopathies. Am J Hum Genet. 2014;95:360-370.

28. Hamdan FF, Myers CT, Cossette P, Lemay P, Spiegelman D, Laporte AD, et al. High Rate of Recurrent De Novo Mutations in Developmental and Epileptic Encephalopathies. Am J Hum Genet. 2017;101:664-685.

29. Helbig KL, Farwell Hagman KD, Shinde DN, Mroske C, Powis Z, Li S, et al. Diagnostic exome sequencing provides a molecular diagnosis for a significant proportion of patients with epilepsy. Genet Med. 2016;18:898-905.

30. Heyne HO, Singh T, Stamberger H, Abou Jamra R, Caglayan H, Craiu D, et al. De novo variants in neurodevelopmental disorders with epilepsy. Nat Genet. 2018;50:1048-1053.

31. Tran Mau-Them F, Guibaud L, Duplomb L, Keren B, Lindstrom K, Marey I, et al. De novo truncating variants in the intronless IRF2BPL are responsible for developmental epileptic encephalopathy. Genet Med. 2019;21:1008-1014.

32. McRae JF CS, Fitzgerald TW, Kaplanis J, Prigmore E, Rajan D et al. Prevalence and architecture of de novo mutations in developmental disorders. Nature. 2017;542:433-438.

33. Genome of the Netherlands C. Whole-genome sequence variation, population structure and demographic history of the Dutch population. Nat Genet. 2014;46:818-825.

34. Kloosterman WP, Francioli LC, Hormozdiari F, Marschall T, Hehir-Kwa JY, Abdellaoui A, et al. Characteristics of de novo structural changes in the human genome. Genome Res. 2015;25:792-801.

35. Rahbari R, Wuster A, Lindsay SJ, Hardwick RJ, Alexandrov LB, Turki SA, et al. Timing, rates and spectra of human germline mutation. Nat Genet. 2016;48:126-133.

36. Goldmann JM, Wong WS, Pinelli M, Farrah T, Bodian D, Stittrich AB, et al. Parent-of-origin-specific signatures of de novo mutations. Nat Genet. 2016;48:935-939.

37. Guo JF, Zhang L, Li K, Mei JP, Xue J, Chen J, et al. Coding mutations in NUS1 contribute to Parkinson's disease. Proc Natl Acad Sci U S A. 2018;115:11567-11572.
